# Supplementary material for: Microwave-Assisted Catalytic Method for a Green Synthesis of Amides Directly from Amines and Carboxylic Acids
Source: Molecules. 2020 Apr 11;25(8):1761. doi: 10.3390/molecules25081761 (PMC7221698; doi:10.3390/molecules25081761)
Supplement: Supplementary file 1 [file molecules-25-01761-s001.pdf]

Supplementary Materials

Microwave-assisted catalytic method for the green synthesis of amides directly from amines and carboxylic acids

Table of contents

General information 3

General procedure 3

Procedure of synthesis of amides with excess acid reactant 3

Procedure for monitoring a progress of reaction by NMR 3

Procedure for a reaction in water (on a smaller scale) 4

Procedure for an optimization of reaction conditions 4

Characterization of products (amides) 5

NMR Spectra of products 17

References 68

General information

All chemicals were purchased from Sigma-Aldrich and Eurisotop (NMR solvents) and used without further purification. ^{1}H NMR and ^{13}C NMR spectra were recorded on an AVANCE III Bruker spectrometer in DMSO-d_6 at frequencies of 500 and 126 MHz, respectively. Spectra were processed using MestReNova Version 9.0.1-13254. NMR data were reported as follows: Chemical shift (δ), multiplicity (recorded as br, broad; s, singlet; d, doublet; t, triplet; q, quadruplet, and m, multiplet), coupling constants (*J* in Hertz, Hz), and integration. The chemical shifts are expressed in parts per million (ppm) and reported in relation to a residual solvent peak (2.50 and 39.5 ppm for ^{1}H and ^{13}C NMR, respectively). High-resolution mass spectrometry (HRMS) was performed on a Q-Exactive Orbitrap mass spectrometer (Thermo Fisher Scientific, Bremen, Germany) equipped with a TriVersa NanoMate robotic nanoflow ESI ion source (Advion BioSciences ltd., Ithaca, USA). The nanoelectrospray chips with a nozzle diameter of 5.5 μm were used in order to obtain a stable ion spray and Chipsoft software (ver. 8.3.1.1018) was applied to control the ESI ion source. Microwave experiments were performed in a commercially available open microwave reactor (Magnum II) from ERTEC-Poland Dr Edward Reszke. The microwave reaction was smoothly regulated in the range from 0 to 750 W at a field frequency of 2.45 GHz. The temperature was monitored by a pyrometer and by monitoring the vapor temperature using a K-type thermocouple. Reactions were carried out at atmospheric pressure in regular glass flasks equipped with a reflux condenser glass topped with a free airflow tube with a drying agent.

General procedure

Amine (4.2 mmol), carboxylic acid (2 mmol), and catalyst (ceric ammonium nitrate) (2 mol%) were added to an empty flask equipped with a reflux condenser, at atmospheric pressure, placed in a microwave set to maintain a constant temperature in the range of 160-165 °C for a given time period (microwave power set up to 480 W but was smoothly and automatically controlled by the software to keep the target temperature constant). After 2 h, the reaction mixture was allowed to cool to room temperature, and subsequently, 25 mL of ethyl acetate were added. The organic phase was washed with 3 x 15 mL of 2 M aqueous HCl, 3 x 15 mL saturated aqueous $NaHCO_3$, and 3 x 15 mL of saturated aqueous NaCl; dried over Na_2SO_4; filtered; and the solvent was removed under reduced pressure to obtain the pure product. The resulting products were characterized by ^{1}H NMR, ^{13}C NMR, and HRMS.

Procedure of synthesis of amides with excess acid reactant

3-(4-hydroxyphenyl)propionic acid (phloretic acid) was used in excess in the case of the reaction with benzylamine, *p*-methylbenzylamine, and putrescine (1,4-diaminobutane). Carboxylic acid (0.698 g; 4.2 mmol, 2.1 eq), amine (1 eq), and ceric ammonium nitrate (0.022 g; 2 mol%) were mixed and the reaction mixture was heated up with microwave radiation for 2 h at a temperature of 160-165 °C in open air. Subsequently, the reaction mixture was allowed to cool to room temperature and then dissolved in 25 mL of ethyl acetate in an ultrasonic bath. The organic phase was washed with 3 x 15 mL of 2 M aqueous HCl x 15 mL saturated aqueous $NaHCO_3$, and 3 x 15 mL of saturated aqueous NaCl; dried over Na_2SO_4; filtered; and the solvent was removed under reduced pressure to obtain the pure product. Products were characterized by ^{1}H NMR, ^{13}C NMR, and HRMS.

Procedure for monitoring a progress of reaction by NMR

Benzylamine (4.2 mmol) and benzoic acid (2 mmol) were mixed and added to an empty flask equipped with a reflux condenser and the flask was transferred to a microwave reactor. After 1 h, an aliquot for ^{1}H NMR was collected, followed by the addition of a catalyst (ceric ammonium nitrate 2 mol%). The mixture was then heated up in a microwave (as in the general procedure) and an aliquot was taken every 30 min and analyzed by ^{1}H NMR (NMR spectra are available in Figure S1).

Procedure for a reaction in water (on a smaller scale)

Toluidine (0.0375 g; 0.35 mmol) and phloretic acid (0.0582 g; 0.35 mmol) were mixed in the presence of 2 mol% (0.0038 g) of a catalyst (ceric ammonium nitrate), with thee total weight of the mixture being 100 mg. The reaction mixture was then heated up with microwave radiation for 30 min, but no increase in the temperature of the mixture could be observed. Subsequently, to this mixture, water was added in two portions (2 x 0.3 mL). After the addition of water, the reaction mixture was heated with microwave radiation to 100 °C (reflux of water). After 2 h, the reaction mixture was allowed to cool to room temperature. Then, the mixture was dissolved in 25 mL of ethyl acetate in an ultrasonic bath. The organic phase was washed with 3 x 15 mL of 2 M aqueous HCl, 3 x 15 mL saturated aqueous $NaHCO_3$, and 3 x 15 mL of saturated aqueous NaCl; dried over Na_2SO_4; filtered; and the solvent was reduced under pressure to obtain a solid in a yield of 0.0813 g (91%).

Procedure for an optimization of reaction conditions

Toluidine (2.1 eq.), carboxylic acid (1 eq.), and catalyst (ceric ammonium nitrate—various quantities) were added to an empty flask equipped with a reflux condenser, placed in a microwave reactor, and then heated up and kept for a given time (from 30 min to 5 h) at different temperature ranges, as presented in Table 1. After 2 h, the reaction mixture was allowed to cool to room temperature and subsequently dissolved in 25 mL of ethyl acetate in an ultrasonic bath. The organic phase was washed with 3 x 15 mL of 2 M aqueous HCl, 3 x 15 mL saturated aqueous $NaHCO_3$, and 3 x 15 mL of saturated aqueous NaCl; dried over Na_2SO_4; filtered; and the solvent was removed under reduced pressure to obtain the pure product. The resulting products were characterized by ^{1}H NMR, and then the isolated yields of the pure products were calculated.

Table S1. Optimization of the reaction conditions for carboxylic acid and *p*-toluidine as the substrate.

Entry	Catalyst	t [h]	T [°C]	Yield [%][a]
Phloretic acid				
1	2 mol%	2	60 – 65	trace
2	2 mol%	2	80 – 85	30
3	2 mol%	2	100 – 105	88
4	2 mol%	2	120 – 125	95
5	2 mol%	2	160 – 165	94
6	2 mol%	0.5	120 – 125	52
7	none	2	120 – 125	71
8	none	5	120 – 125	85
9	0.1 mol%	2	120 – 125	79
10	0.1 mol%	3	120 – 125	87
11	0.1 mol%	5	120 – 125	93
Benzoic acid				
12	2 mol%	2	80 – 85	trace
13	2 mol%	2	100 – 105	trace
14	2 mol%	2	120 – 125	18
15	2 mol%	2	140 – 145	45
16	2 mol%	2	160 – 165	75
17	2 mol%	5	160 – 165	95
18	none	2	160 – 165	trace
19	0.1 mol%	2	160 – 165	trace

Phenylacetic acid				
20	none	2	120 – 125	73
21	none	2	160 – 165	87
22	2 mol%	2	120 – 125	71
23	2 mol%	2	160 – 165	98

a) Isolated yields from 2 independent repeats (average of these attempts)

Characterization of products (amides)

N-phenylbenzamide (1)

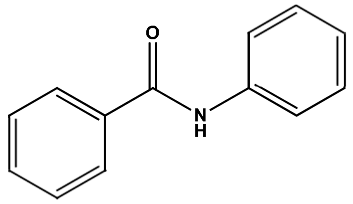

The compound was isolated following the general procedure described above and obtained as a solid (yield = 69–71% - 2 independent repeats). ^{1}H NMR (500 MHz, DMSO-d$_6$) δ = 10.32 (s, 1H), 7.98 (d, J = 7.0 Hz, 2H), 7.81 (d, J = 7.3 Hz, 2H), 7.59 (t, J = 7.3 Hz, 1H), 7.53 (t, J = 7.5 Hz, 2H), 7.39 – 7.32 (m, 2H), 7.10 (t, J = 7.4 Hz, 1H). ^{13}C NMR (126 MHz, DMSO-d$_6$) δ = 166.0, 139.7, 135.4, 132.0, 129.0, 128.8, 128.2, 124.1, 120.9. HRMS[2M+Na]$^+$ calcd for $C_{13}H_{11}NO$: 417.1574, found: 417.1524.

This compound was previously described in the literature.[1]

3-(4-hydroxyphenyl)-*N*-phenylpropanamide (2)

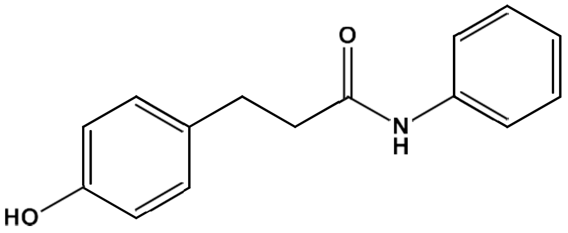

The compound was isolated following the general procedure described above and obtained as a solid (yield = 94% – 3 independent repeats). ^{1}H NMR (500 MHz, DMSO-d$_6$) δ = 9.86 (s, 1H), 9.15 (s, 1H), 7.62–7.54 (m, 2H), 7.28 (t, J = 7.9 Hz, 2H), 7.06–6.99 (m, 3H), 6.70–6.62 (m, 2H), 2.80 (t, J = 7.7 Hz, 2H), 2.56 (t, J = 7.7 Hz, 2H). ^{13}C NMR (126 MHz, DMSO-d$_6$) δ = 171.0, 155.9, 139.7, 131.7, 129.5, 129.1, 123.4, 119.5, 115.5, 38.9, 30.6. HRMS[M+H]$^+$ calcd for $C_{15}H_{15}NO_2$: 242.1175, found: 242.1154.

N-*p*-tolylbenzamide (3)

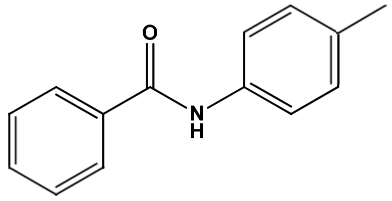

The compound was isolated following the general procedure described above and obtained as a solid (yield = 72–78% - 2 independent repeats). ^{1}H NMR (500 MHz, DMSO-d$_6$) δ = 10.17 (s, 1H), 7.99–7.93 (m, 2H), 7.67 (d, J = 8.5 Hz, 2H), 7.61–7.50 (m, 3H), 7.16 (d, J = 8.2 Hz, 2H), 2.29 (s, 3H). ^{13}C NMR (126 MHz, DMSO-d$_6$) δ = 165.8, 137.1, 135.5, 133.0, 131.9, 129.4, 128.8, 128.0, 120.8, 21.0. HRMS[M+H]$^+$ calcd for $C_{14}H_{13}NO$: 212.1069, found: 212.1060.

This compound was previously reported in the literature.[1]

3-(4-hydroxyphenyl)-*N*-(*p*-tolyl)propanamide (4)

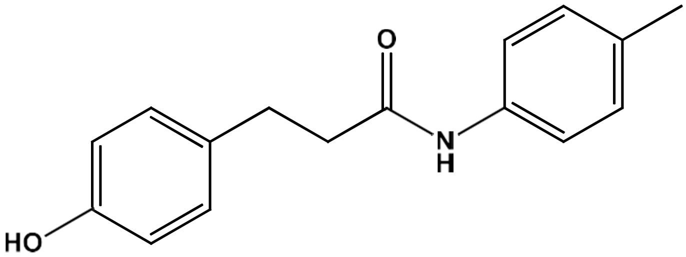

The compound was isolated following the general procedure described above and obtained as a solid (yield = 89–95% - 2 independent repeats). ^{1}H NMR (500 MHz; DMSO-d$_6$) δ = 9.77 (s, 1H), 9.15 (s, 1H), 7.46 (d, *J* = 8.2 Hz, 2H), 7.08 (d, *J* = 8.2 Hz, 2H), 7.03 (d, *J* = 8.2 Hz, 2H), 6.67 (d, *J* = 8.4 Hz, 2H), 2.79(t, *J* = 8.7, 6.7 Hz, 2H), 2.53 (t, *J* = 8.7, 6.8 Hz, 2H), 2.23 (s, 3H). ^{13}C NMR (126 MHz, DMSO-d$_6$) δ = 170.8, 155.9, 137.2, 132.3, 131.7, 129.5, 129.5, 119.6, 115.5, 38.9, 30.6, 20.9. HRMS[M+H]$^+$ calcd for $C_{16}H_{17}NO_2$: 256.1321, found: 256.1332.

N-(4-methoxyphenyl)benzamide (5)

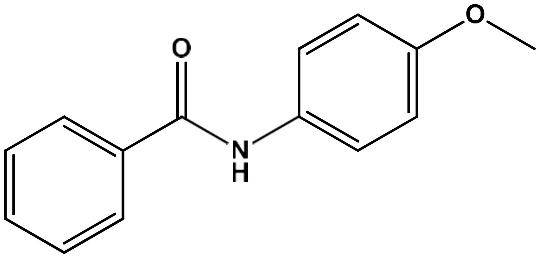

The compound was isolated following the general procedure described above and obtained as a solid (yield = 52–54% - 2 independent repeats). ^{1}H NMR (500 MHz, DMSO-d$_6$) δ = 10.14 (s, 1H), 7.98–7.93 (m, 2H), 7.71–7.68 (m, 2H), 7.60–7.49 (m, 3H), 6.96–6.90 (m, 2H), 3.75 (s, 3H). ^{13}C NMR (126 MHz, DMSO-d$_6$) δ = 165.6, 156.0, 135.5, 132.7, 131.8, 128.8, 128.0, 122.4, 114.2, 55.6. HRMS[M+H]$^+$ calcd for $C_{14}H_{13}NO_2$: 228.1018, found: 242.1005.
This compound was previously described in the literature.[1]

3-(4-hydroxyphenyl)-*N*-(4-methoxyphenyl)propanamide (6)

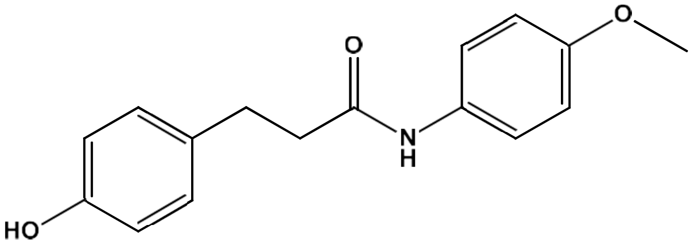

The compound was isolated following the general procedure described above and obtained as a solid (yield = 98% - 3 independent repeats). ^{1}H NMR (500 MHz; DMSO-d$_6$) δ = 9.72 (s, 1H), 9.15 (s, 1H), 7.51–7.45 (m, 2H), 7.03 (d, *J* = 8.4 Hz, 2H), 6.89–6.83 (m, 2H), 6.70–6.65 (m, 2H), 3.71 (s, 3H), 2.79 (t, *J* = 8.7, 6.8 Hz, 2H), 2.52 (t, 2H). ^{13}C NMR (126 MHz, DMSO-d$_6$) δ = 170.5, 155.9, 155.5, 132.9, 131.7, 129.5, 121.1, 115.5, 114.2, 55.6, 38.8, 30.7. HRMS[M+H]$^+$ calcd for $C_{16}H_{17}NO_3$: 272.1281, found: 272.1266.

N-(4-fluorophenyl)benzamide (7)

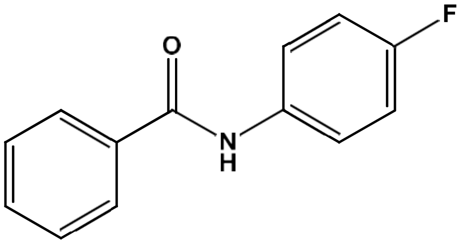

The compound was isolated following the general procedure described above and obtained as a solid (yield = 75% - 3 independent repeats). ^{1}H NMR (500 MHz, DMSO-d_6) δ = 10.31 (s, 1H), 7.96 (d, J = 7.6 Hz, 2H), 7.81 (dd, J = 8.8, 5.1 Hz, 2H), 7.57 (dt, J = 30.7, 7.4 Hz, 3H), 7.20 (t, J = 8.7 Hz, 2H). ^{13}C NMR (126 MHz, DMSO-d_6) δ = 165.9, 159.7, 157.8, 136.0, 136.0, 135.3, 132.1, 128.9, 128.1, 122.7, 122.6, 115.7, 115.5. HRMS[M+H]$^+$ calcd for $C_{13}H_{10}FNO$: 216.0818, found: 216.0806.
This compound was previously described in the literature.[2]

3-(4-hydroxyphenyl)-*N*-(4-fluorophenyl)propanamide (8)

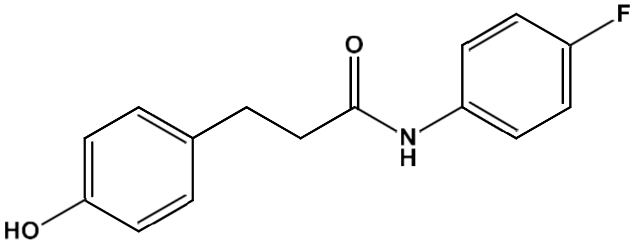

The compound was isolated following the general procedure described above and obtained as a solid (yield = 89–92% - 2 independent repeats). ^{1}H NMR (500 MHz, DMSO-d_6) δ = 9.93 (s, 1H), 9.16 (s, 1H), 7.64 – 7.56 (m, 2H), 7.14 – 7.08 (m, 2H), 7.06 – 7.01 (m, 2H), 6.71 – 6.64 (m, 2H), 2.81 (t, 2H), 2.55 (t, J = 7.7 Hz, 2H). ^{13}C NMR (126 MHz, DMSO-d_6) δ = 170.9, 159.3, 157.3, 156.0, 136.1, 136.1, 131.6, 129.5, 121.3, 121.2, 115.7, 115.5, 49.1, 38.9, 30.6. HRMS[M+H]$^+$ calcd for $C_{15}H_{14}FNO_2$: 260.1081, found: 260.1067.

N-benzylbenzamide (9)

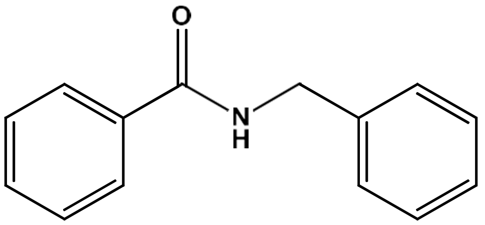

The compound was isolated following the general procedure described above and obtained as a solid (yield = 71–77% - 2 independent repeats).^{1}H NMR (500 MHz, DMSO-d_6) δ = 9.09 (t, J = 6.1 Hz, 1H), 7.96 –7.93 (m, 2H), 7.56–7.46 (m, 3H), 7.37–7.22 (m, 5H), 4.53 (d, J = 6.0 Hz, 2H).^{13}C NMR (126 MHz, DMSO-d_6) δ = 166.7, 140.2, 134.8, 131.7, 128.8, 128.7, 127.7, 127.7, 127.2, 43.1.HRMS low resolution.
This compound was previously described in the literature.[3]

3-(4-hydroxyphenyl)-*N*-(phenylmethyl)propanamide (10)

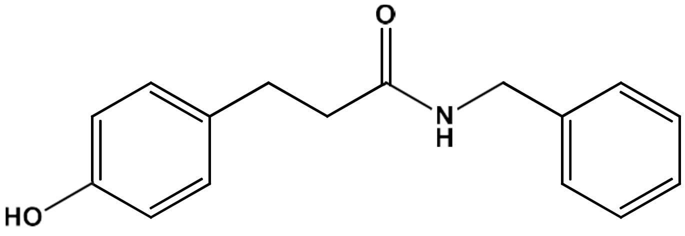

The compound was isolated following the general procedure described above and obtained as a solid (yield = 74–88% - 2 independent repeats). ^{1}H NMR (500 MHz, DMSO-d$_6$) δ = 9.17 (s, 1H), 8.30 (t, J = 6.0 Hz, 1H), 7.30 – 7.18 (m, 3H), 7.14–7.10 (m, 2H), 7.04–6.96 (m, 2H), 6.68–6.64 (m, 2H), 4.24 (d, J = 5.9 Hz, 2H), 2.73 (t, J = 7.6 Hz, 2H), 2.39 (t, J = 7.6 Hz, 2H). ^{13}C NMR (126 MHz, DMSO-d$_6$) δ = 171.4, 155.5, 139.5, 131.3, 129.2, 128.2, 127.1, 126.6, 115.0, 41.9, 37.5, 30.4. HRMS[M+H]$^+$ calcd for $C_{16}H_{17}NO_2$: 256.1331, found: 256.1312.
This compound was previously described in the literature.[4]

N-(4-methylbenzyl)benzamide (11)

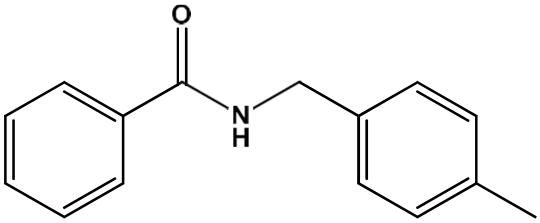

The compound was isolated following the general procedure described above and obtained as a solid (yield = 70% - 2 independent repeats). ^{1}H NMR (500 MHz, DMSO-d$_6$) δ = 9.01 (t, J = 6.1 Hz, 1H), 7.93–7.88 (m, 2H), 7.56–7.45 (m, 3H), 7.22 (d, J = 8.0 Hz, 2H), 7.13 (d, J = 7.9 Hz, 2H), 4.45 (d, J = 6.0 Hz, 2H), 2.28 (s, 3H). ^{13}C NMR (126 MHz, DMSO-d$_6$) δ = 166.6, 137.1, 136.2, 134.9, 131.6, 129.3, 129.2, 128.8, 128.5, 127.7, 42.8, 21.1. HRMS[M+H]$^+$ calcd for $C_{15}H_{15}NO$: 226.1226, found: 226.1573.
This compound was previously described in the literature.[5]

3-(4-hydroxyphenyl)-*N*-[(4-methylphenyl)methyl]propanamide (12)

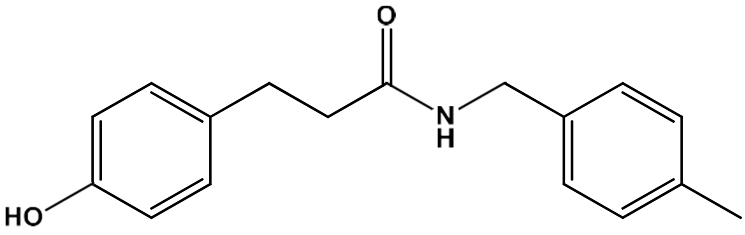

The compound was isolated following the general procedure described above and obtained as a solid (yield = 70% - 2 independent repeats). ^{1}H NMR (500 MHz, DMSO-d$_6$) δ = 9.17 (s, 1H), 8.25 (t, J = 5.9 Hz, 1H), 7.08 (d, J = 7.8 Hz, 2H), 7.03–6.96 (m, 4H), 6.68–6.63 (m, 2H), 4.19 (d, J = 5.9 Hz, 2H), 2.72 (dd, J = 8.4, 6.9 Hz, 2H), 2.37 (dd, J = 8.5, 6.9 Hz, 2H), 2.26 (s, 3H). ^{13}C NMR (126 MHz, DMSO-d$_6$) δ = 171.4, 155.5, 136.5, 135.6, 131.3, 129.2, 128.7, 127.1, 115.0, 41.7, 37.5, 30.4, 20.7. HRMS[M+H]$^+$ calcd for $C_{17}H_{19}NO_2$: 270.1488, found: 270.1476.

N-(4-fluorobenzyl)benzamide (13)

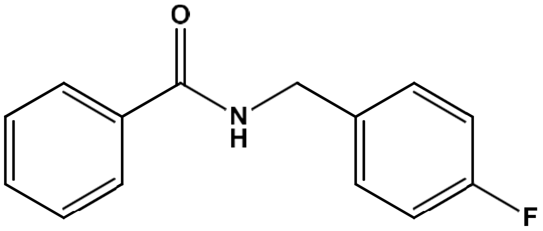

The compound was isolated following the general procedure described above and obtained as a solid (yield = 80–87% - 2 independent repeats). ^{1}H NMR (500 MHz, DMSO-d_6) δ = 9.06 (t, *J* = 6.1 Hz, 1H), 7.93–7.88 (m, 2H), 7.56–7.44 (m, 3H), 7.41–7.34 (m, 2H), 7.19–7.12 (m, 2H), 4.48 (d, *J* = 5.9 Hz, 2H). ^{13}C NMR (126 MHz, DMSO-d_6) δ = 166.2, 162.1, 160.2, 135.9, 135.8, 134.2, 131.2, 129.2, 129.1, 128.3, 127.2, 115.0, 114.9, 41.9. HRMS[M+NH$_4$]$^+$ calcd for $C_{14}H_{12}FNO$: 247.1241, found: 247.1030. This compound was previously described in the literature.[6]

3-(4-hydroxyphenyl)-*N*-(4-fluorobenzyl)propanamide (14)

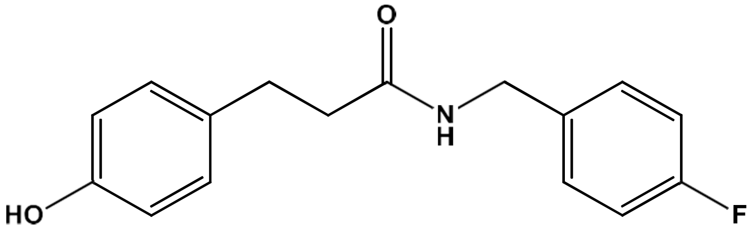

The compound was isolated following the general procedure described above and obtained as a solid (yield = 92–94% - 2 independent repeats). ^{1}H NMR (500 MHz, DMSO-d_6) δ = 9.17 (s, 1H), 8.30 (t, *J* = 6.0 Hz, 1H), 7.18–7.06 (m, 4H), 7.02–6.98 (m, 2H), 6.70–6.66 (m, 2H), 4.24 (d, *J* = 6.0 Hz, 2H), 2.75 (t, *J* = 7.6 Hz, 2H), 2.40 (t, *J* = 7.6 Hz, 2H). ^{13}C NMR (126 MHz, DMSO-d_6) δ = 171.9, 162.5, 160.6, 156.0, 136.2, 131.7, 129.6, 129.5, 129.4, 115.5, 115.4, 115.2, 41.7, 37.9, 30.8. HRMS[M+H]$^+$ calcd for $C_{16}H_{16}FNO_2$: 274.1237, found: 274.1215.

N-(furan-2-ylmethyl)benzamide (15)

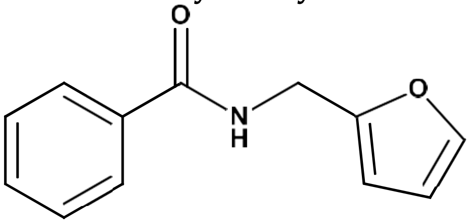

The compound was isolated following the general procedure described above and obtained as a solid (yield = 84% - 2 independent repeats). ^{1}H NMR (500 MHz, DMSO-d_6) δ = 8.97 (t, *J* = 5.8 Hz, 1H), 7.90–7.86 (m, 2H), 7.58 (dd, *J* = 1.8, 0.9 Hz, 1H), 7.55–7.44 (m, 3H), 6.40 (dd, *J* = 3.2, 1.9 Hz, 1H), 6.28 (dd, *J* = 3.2, 0.9 Hz, 1H), 4.48 (d, *J* = 5.7 Hz, 2H). ^{13}C NMR (126 MHz, DMSO-d_6) δ = 166.5, 152.9, 142.4, 134.6, 131.8, 128.8, 127.7, 110.9, 107.3, 36.5. HRMS[2M+Na]$^+$ calcd for $C_{12}H_{11}NO_2$: 425.1472, found: 425.1438. This compound was previously described in the literature.[7]

3-(4-hydroxyphenyl)-*N*-(furan-2-ylmethyl)propanamide (16)

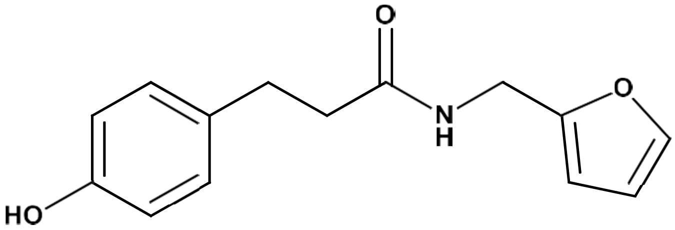

The compound was isolated following the general procedure described above and obtained as a solid (yield = 91% - 3 independent repeats). ^{1}H NMR (500 MHz, DMSO-d_6) δ = 9.13 (s, 1H), 8.26 (t, *J* = 5.7 Hz, 1H), 7.57–7.54 (m, 1H), 7.00–6.95 (m, 2H), 6.67–6.62 (m, 2H), 6.37 (dd, *J* = 3.2, 1.9 Hz, 1H), 6.16 (d, *J* = 3.1 Hz, 1H), 4.24 (d, *J* = 5.6 Hz, 2H), 2.70 (dd, *J* = 8.7, 6.9 Hz, 2H), 2.34 (dd, *J* = 8.8, 6.8 Hz, 2H). ^{13}C NMR (126 MHz, DMSO-d_6) δ = 171.8, 155.9, 152.9, 142.4, 131.8, 129.5, 115.5, 110.9, 107.1, 37.8, 35.8, 30.7. HRMS$[M+H]^+$ calcd for $C_{14}H_{15}NO_3$: 246.1124, found: 246.1105.

***N*-hexylbenzamide (17)**

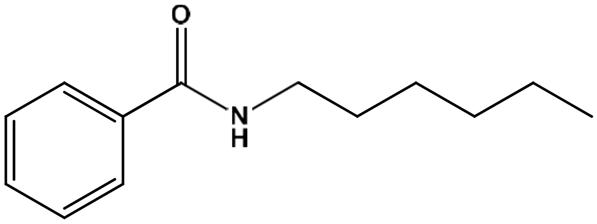

The compound was isolated following the general procedure described above and obtained as a solid (yield = 57–61% - 2 independent repeats). ^{1}H NMR (500 MHz, DMSO-d_6) δ = 8.43 (t, *J* = 5.7 Hz, 1H), 7.87–7.81 (m, 2H), 7.54–7.41 (m, 3H), 3.25 (q, *J* = 7.2, 5.7 Hz, 2H), 1.56 – 1.48 (m, 2H), 1.29 (qt, *J* = 7.4, 3.1 Hz, 6H), 0.92–0.81 (m, 3H). ^{13}C NMR (126 MHz, DMSO-d_6) δ = 166.5, 135.2, 131.4, 128.6, 127.6, 39.7, 31.5, 29.6, 26.7, 22.5, 14.4. HRMS$[M+ACN+Na]^+$ calcd for $C_{13}H_{19}NO$: 269.1624, found: 269.2933.
This compound was previously described in the literature.[8]

3-(4-hydroxyphenyl)-*N*-hexylpropanamide (18)

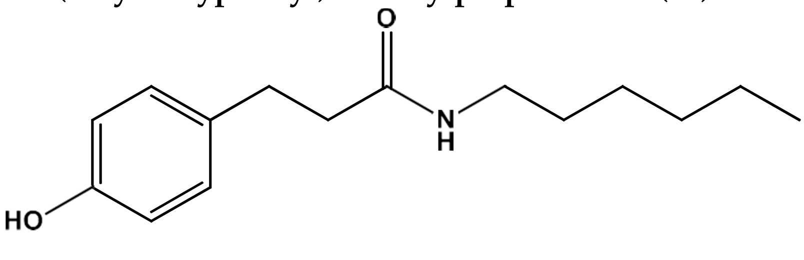

The compound was isolated following the general procedure described above and obtained as a solid (yield = 88% - 2 independent repeats). ^{1}H NMR (500 MHz, DMSO-d_6) δ = 9.12 (s, 1H), 7.73 (t, *J* = 5.7 Hz, 1H), 6.99–6.94 (m, 2H), 6.67–6.62 (m, 2H), 3.01 (q, *J* = 6.6 Hz, 2H), 2.68 (dd, *J* = 8.6, 6.9 Hz, 2H), 2.28 (dd, *J* = 8.6, 6.9 Hz, 2H), 1.38–1.20 (m, 11H), 0.86 (t, *J* = 7.0 Hz, 3H). ^{13}C NMR (126 MHz, DMSO-d_6) δ = 171.7, 155.9, 131.9, 129.4, 115.4, 38.9, 38.0, 31.5, 30.9, 29.6, 26.5, 22.5, 14.5. HRMS low resolution.

Uvariadiamide (19)

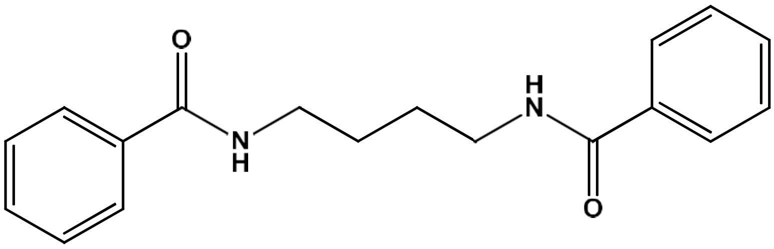

The compound was isolated following the general procedure described above and obtained as a solid (yield = 78% - 3 independent repeats). ^{1}H NMR (500 MHz, DMSO-d$_6$) δ = 8.48 (t, *J* = 5.7 Hz, 2H), 7.88–7.82 (m, 4H), 7.54–7.42 (m, 6H), 3.30 (dd, *J* = 7.0, 4.0 Hz, 4H), 1.63–1.54 (m, *J* = 4.1, 3.7 Hz, 4H). ^{13}C NMR (126 MHz, DMSO-d$_6$) δ = 166.6, 135.2, 131.4, 128.7, 127.6, 39.4, 27.2. HRMS[2M+Na]$^+$ calcd for $C_{18}H_{20}N_2O_2$: 615.2942, found: 615.2896.
This compound was previously described in the literature.[9]

N,N′-(butane-1,4-diyl)bis(3-(4-hydroxyphenyl)propanamide) (20)

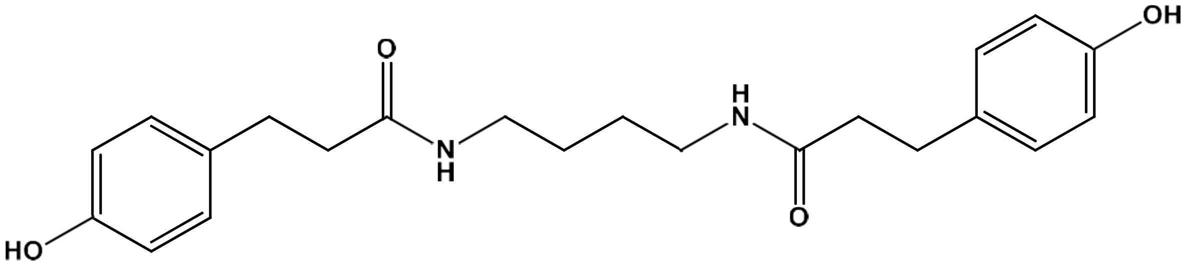

The compound was isolated following the general procedure described above and obtained as a solid (yield = 70–74% - 3 independent repeats). ^{1}H NMR (500 MHz, DMSO-d$_6$) δ = 9.12 (s, 2H), 7.74 (t, *J* = 5.6 Hz, 2H), 6.99–6.95 (m, 4H), 6.67–6.63 (m, 4H), 3.00 (q, *J* = 5.6 Hz, 4H), 2.72–2.65 (m, 4H), 2.32–2.25 (m, 4H), 1.31 (p, *J* = 3.2 Hz, 4H). ^{13}C NMR (126 MHz, DMSO-d$_6$) δ = 171.7, 155.9, 131.9, 129.5, 115.5, 38.6, 38.0, 30.9, 27.0. HRMS[M+H]$^+$ calcd for $C_{22}H_{28}N_2O_4$: 385.2121, found: 385.2086.

N-(4-methoxyphenyl)-2-phenylacetamide (21)

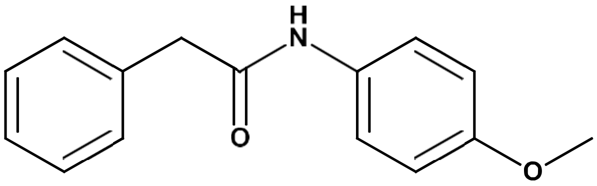

The compound was isolated following the general procedure described above and isolated as a solid (yield = 85% - 2 independent repeats). ^{1}H NMR (500 MHz, DMSO-d$_6$) δ = 10.12 (s, 1H), 7.54 (d, *J* = 9.1 Hz, 2H), 7.37–7.29 (m, 4H), 7.26–7.21 (m, 1H), 6.87 (d, *J* = 9.0 Hz, 2H), 3.70 (s, 3H), 3.62 (s, 2H). ^{13}C NMR (126 MHz, DMSO-d$_6$) δ = 168.6, 155.1, 136.2, 132.4, 129.1, 128.3, 126.5, 120.6, 113.8, 55.1, 43.2. HRMS[M+Na]$^+$ calcd for $C_{15}H_{15}NO_2$: 264.0995, found: 264.0993.
This compound was previously described in the literature.[10]

N-(4'-methoxyphenyl)-4-methylpentanamide (22)

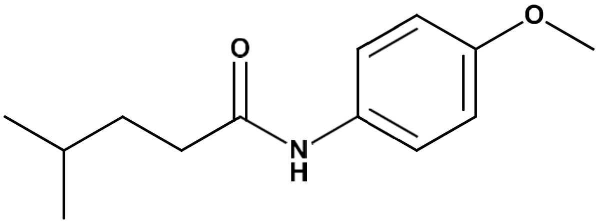

The compound was isolated following the general procedure described above and obtained as a solid (yield = 98% - 2 independent repeats). ^{1}H NMR (500 MHz, DMSO-d_6) δ = 9.75 (s, 1H), 7.50 (d, J = 9.0 Hz, 2H), 6.85 (d, J = 9.0 Hz, 2H), 3.70 (s, 3H), 2.29–2.24 (m, 2H), 1.54 (dp, J = 12.6, 6.4 Hz, 1H), 1.50–1.44 (m, 2H), 0.88 (d, J = 6.5 Hz, 6H). ^{13}C NMR (126 MHz, DMSO-d_6) δ = 170.9, 155.0, 132.6, 120.5, 113.7, 55.1, 34.4, 34.2, 27.3, 22.3. HRMS[M+Na]$^+$ calcd for $C_{13}H_{19}NO_2$: 244.1308, found: 244.1308.

N-(4-fluorobenzyl)-2-phenylacetamide (23)

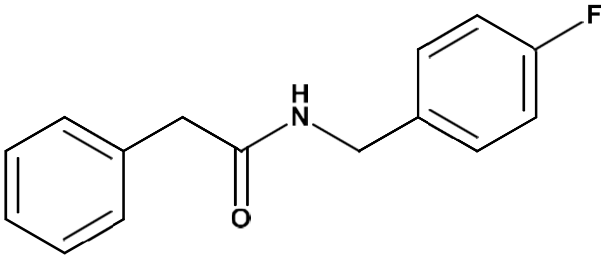

The compound was isolated following the general procedure described above and obtained as a solid (yield = 96% - 2 independent repeats). ^{1}H NMR (500 MHz, DMSO-d_6) δ = 8.59 (t, J = 6.0 Hz, 1H), 7.33–7.20 (m, 7H), 7.16–7.09 (m, 2H), 4.25 (d, J = 5.9 Hz, 2H), 3.48 (s, 2H). ^{13}C NMR (126 MHz, DMSO-d_6) δ = 170.2, 162.1, 160.2, 136.4, 135.7, 129.2, 129.2, 129.0, 128.2, 126.4, 115.1, 114.9, 42.4, 41.5. HRMS[M+Na]$^+$ calcd for $C_{15}H_{14}FNO$: 266.0951, found: 266.0921.
This compound was previously described in the literature.[11]

N-(4-fluorobenzyl)-4-methylpentanamide (24)

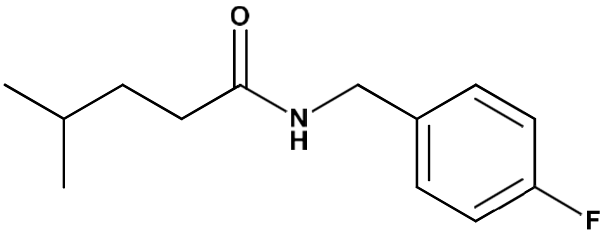

The compound was isolated following the general procedure described above and obtained as a solid (yield = 88% - 2 independent repeats). ^{1}H NMR (500 MHz, DMSO-d_6) δ = 8.34 (t, J = 6.0 Hz, 1H), 7.32–7.23 (m, 2H), 7.19–7.09 (m, 2H), 4.23 (d, J = 6.0 Hz, 2H), 2.17–2.10 (m, 2H), 1.49 (dt, J = 13.2, 6.6 Hz, 1H), 1.44–1.38 (m, 2H), 0.85 (d, J = 6.6 Hz, 6H). ^{13}C NMR (126 MHz, DMSO-d_6) δ = 172.3, 162.1, 160.1, 136.0, 136.0, 129.1, 129.1, 115.0, 114.9, 41.3, 34.3, 33.4, 27.2, 22.3. HRMS[M+H]$^+$ calcd for $C_{13}H_{18}FNO$: 224.1444, found: 224.1442.

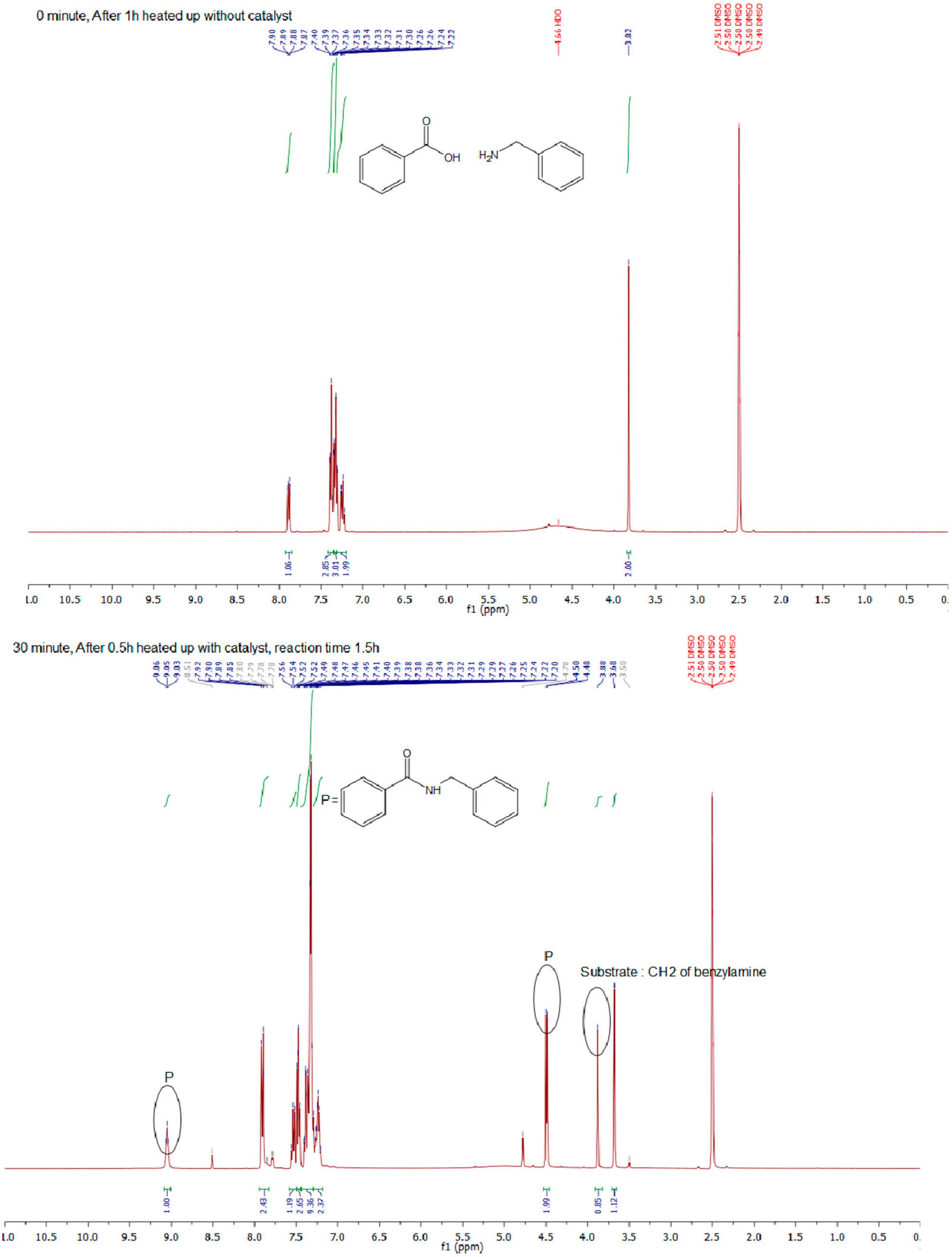

Figure S1. ^{1}H NMR Spectra for the analysis of products measured at different times, compound 9.

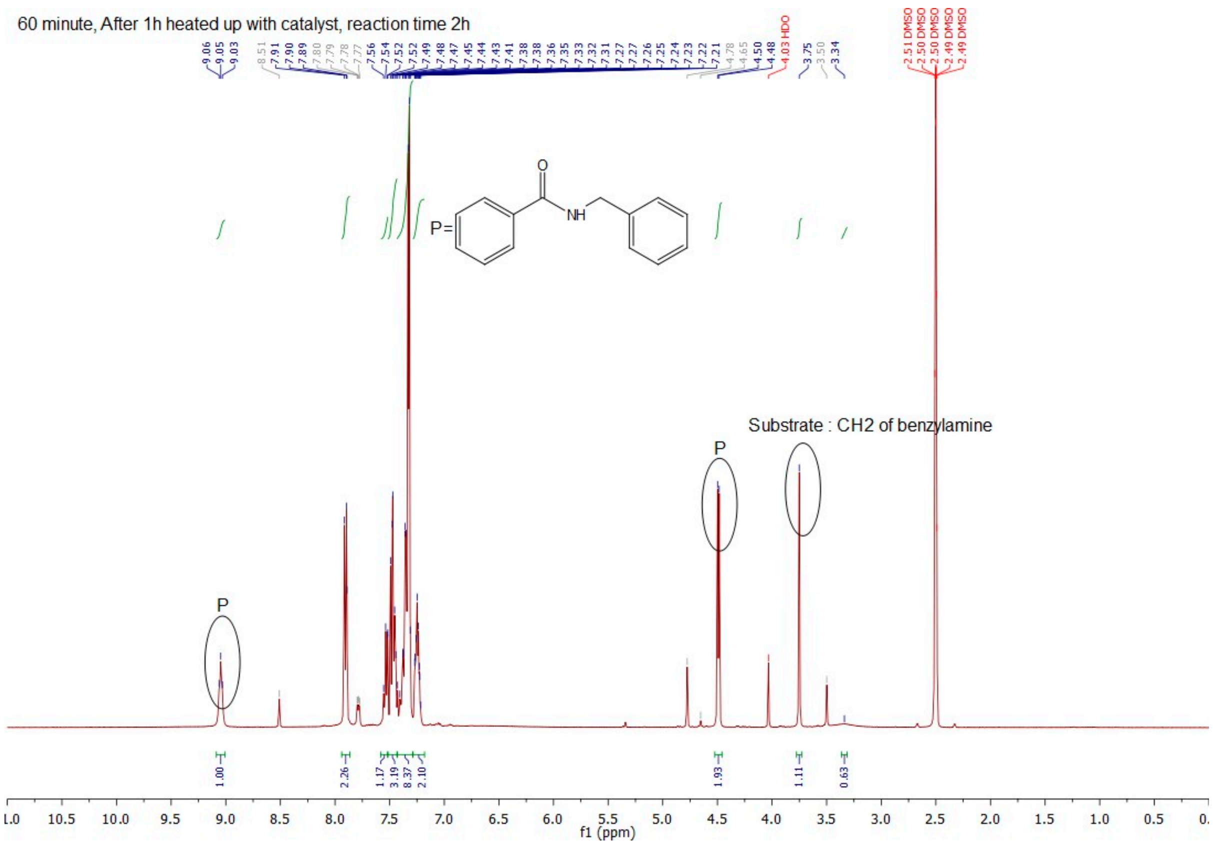

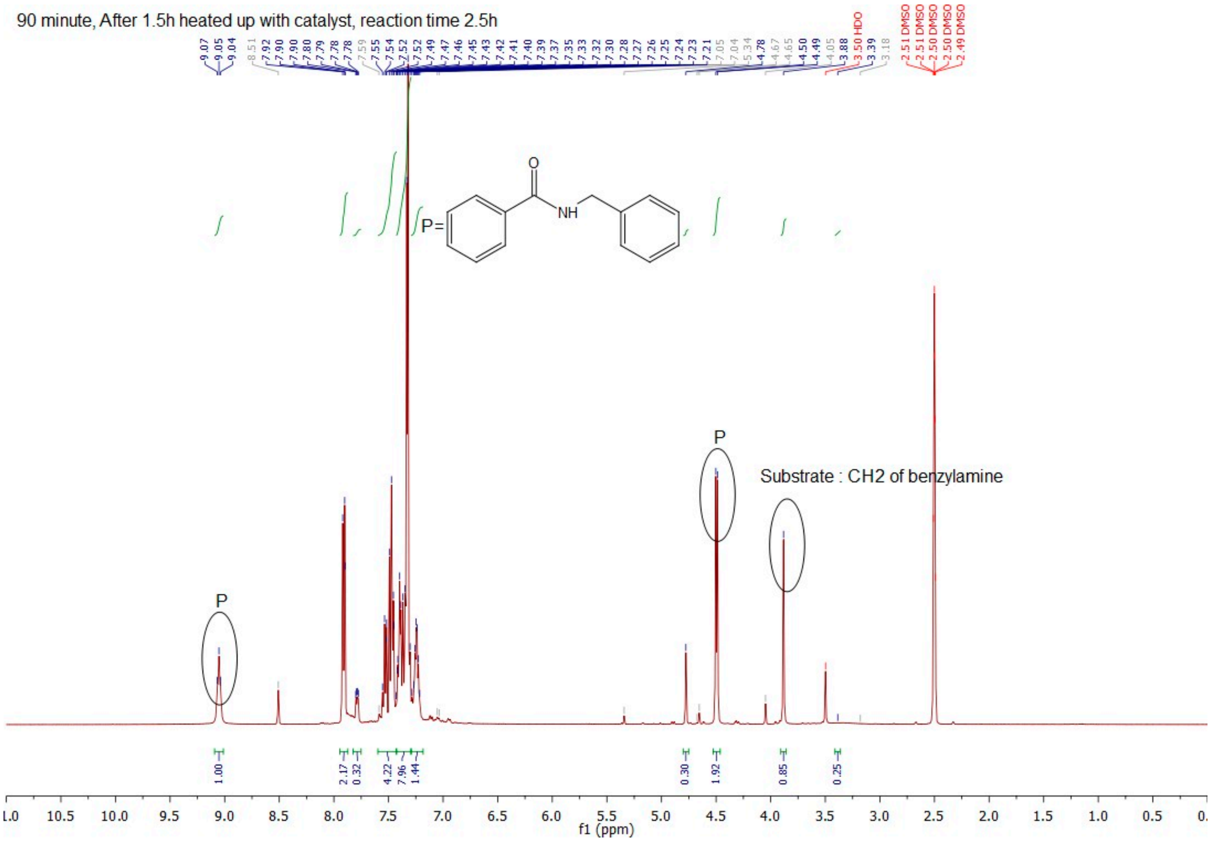

Figure S1. ^{1}H NMR Spectra for the analysis of products measured at different times, compound 9, continued.

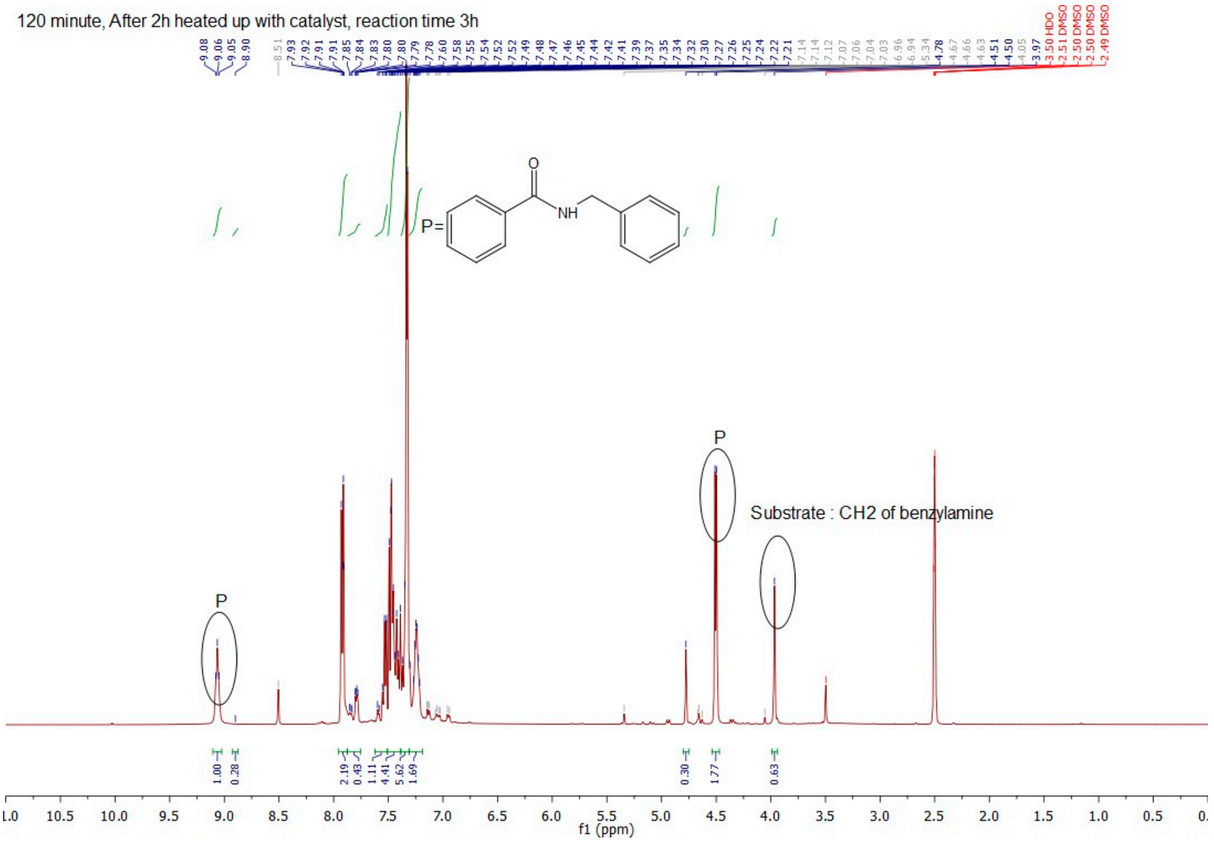

Figure S1. **^{1}H NMR Spectra for the analysis of products measured at different times, compound 9, continued.**

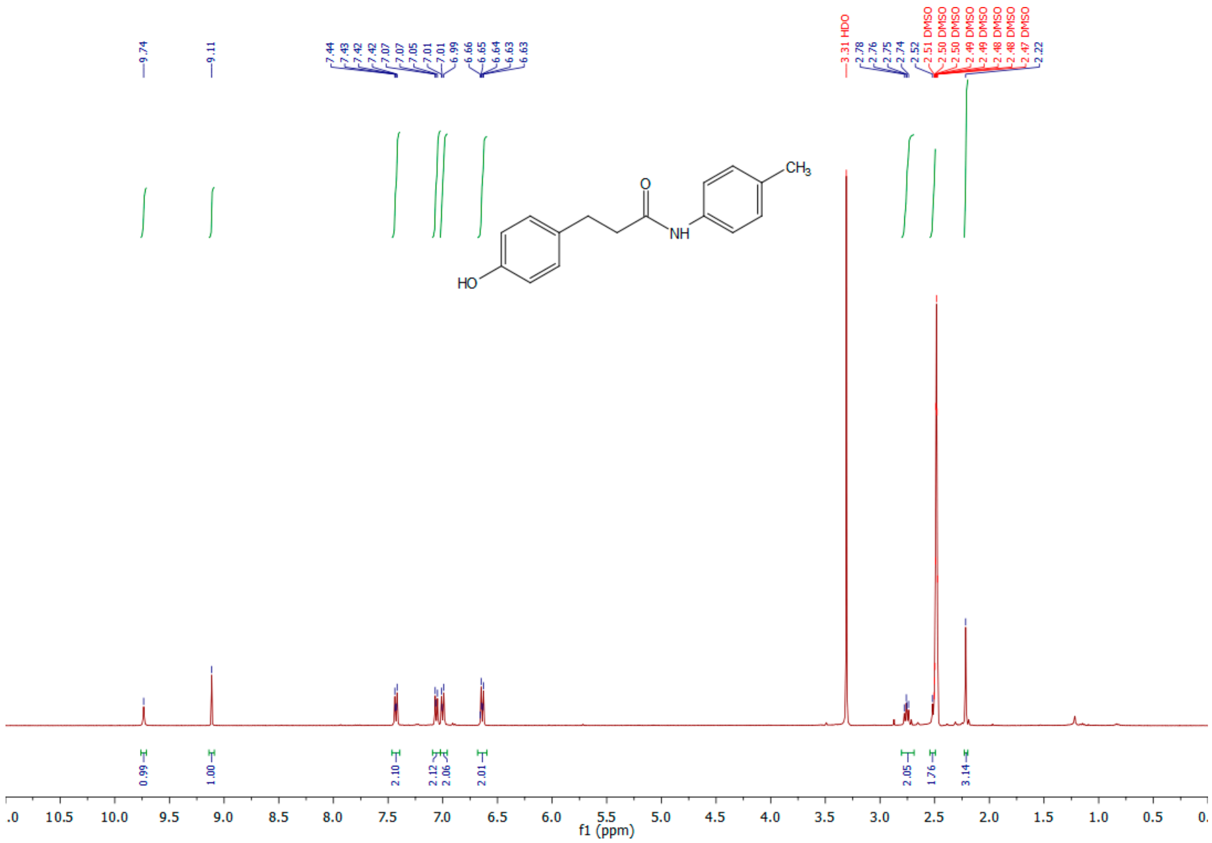

Figure S2. **^{1}H NMR Spectra from the product obtained from the procedure for a reaction in water (on a smaller scale).**

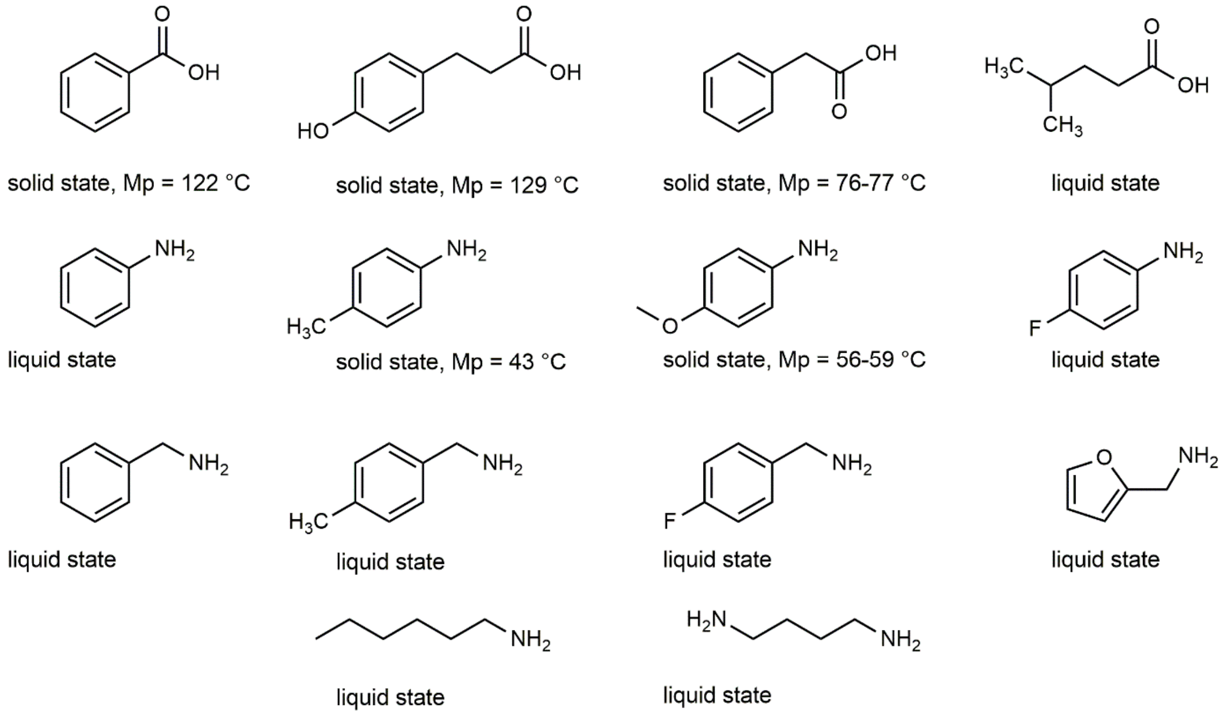

Figure S3. Physical state of substrates at RT.

NMR Spectra of products

Solvents are marked gray on the NMR spectra:

Ethyl acetate: CH_3CO – s, 1.99 ppm
$C\mathit{H}_2CH_3$ –q, 4.03 ppm
$CH_2C\mathit{H}_3$ – t, 1.17 ppm

Dichloromethane : CH_2 – s, 5.76 ppm

Methanol: CH_3 – s, 3.18 ppm
OH – s, 4.01 ppm

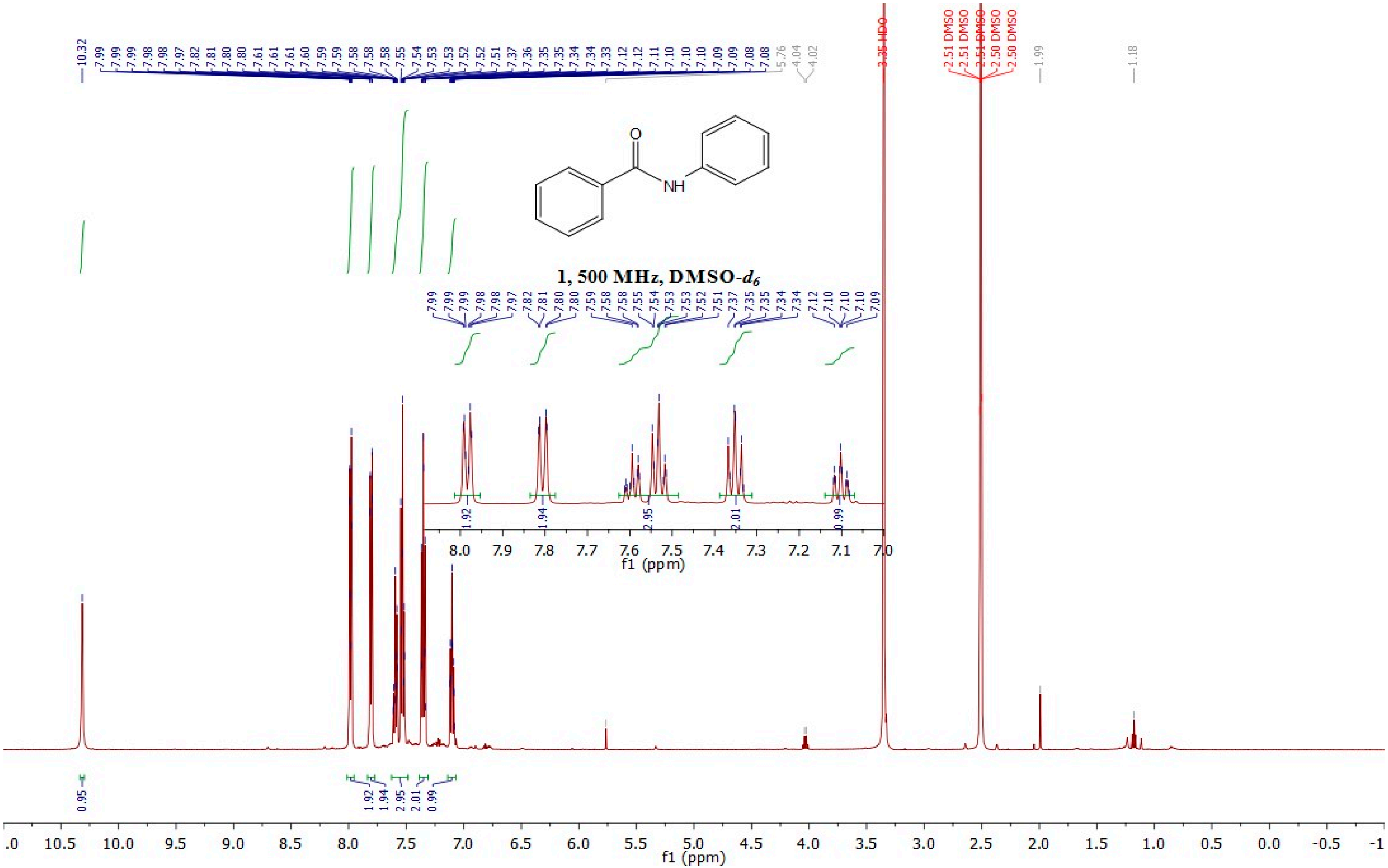
1, 500 MHz, DMSO-d6
O
NH
f1 (ppm)
3.35 HDO
2.51 DMSO
2.51 DMSO
2.51 DMSO
2.50 DMSO
2.50 DMSO

Figure S4.

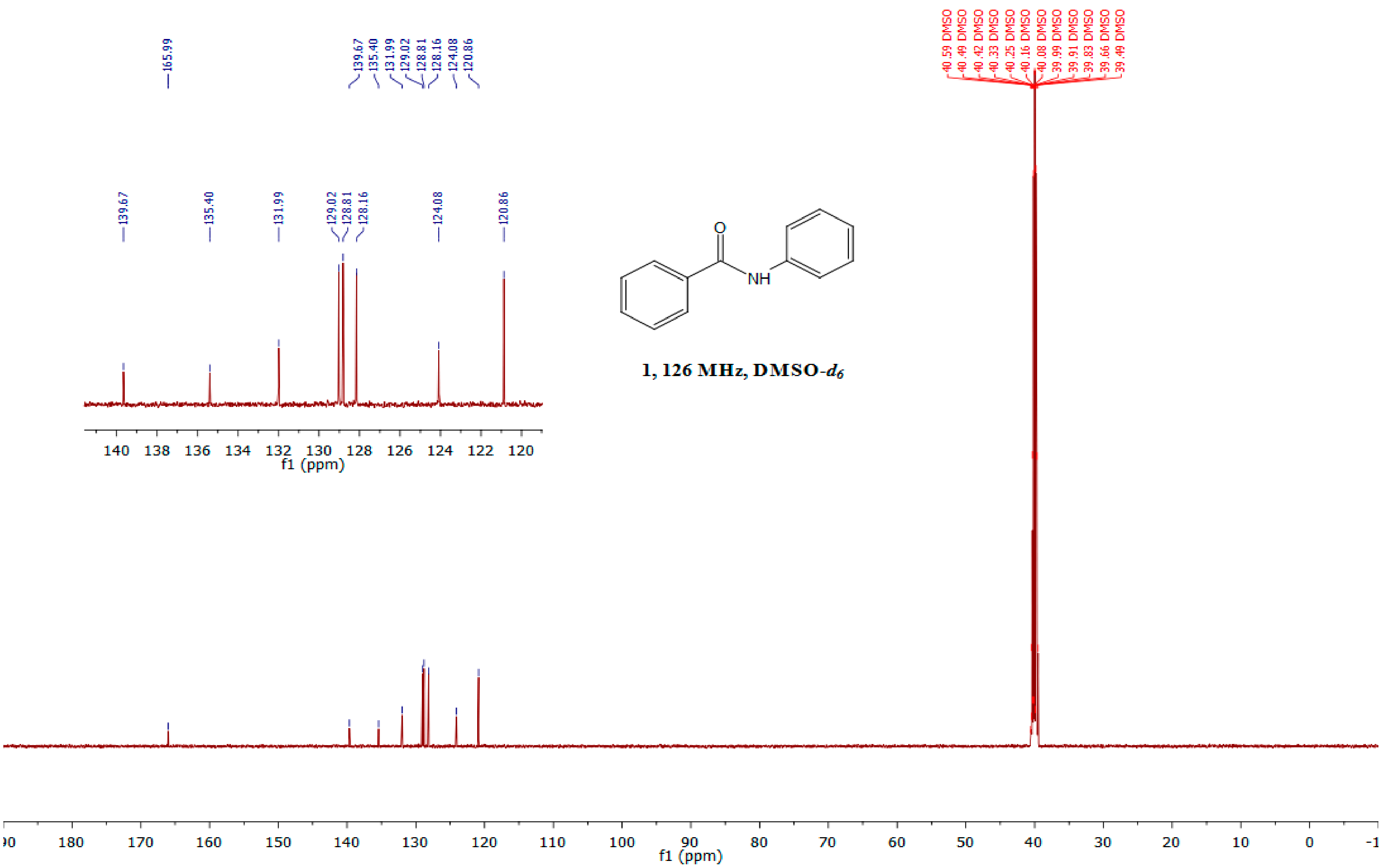
165.99
139.67
135.40
131.99
129.02
128.81
128.16
124.08
120.86
40.59 DMSO
40.49 DMSO
40.42 DMSO
40.33 DMSO
40.25 DMSO
40.16 DMSO
40.08 DMSO
39.99 DMSO
39.91 DMSO
39.83 DMSO
39.66 DMSO
39.49 DMSO
O
NH
1, 126 MHz, DMSO-d_6
f1 (ppm)

Figure S5.

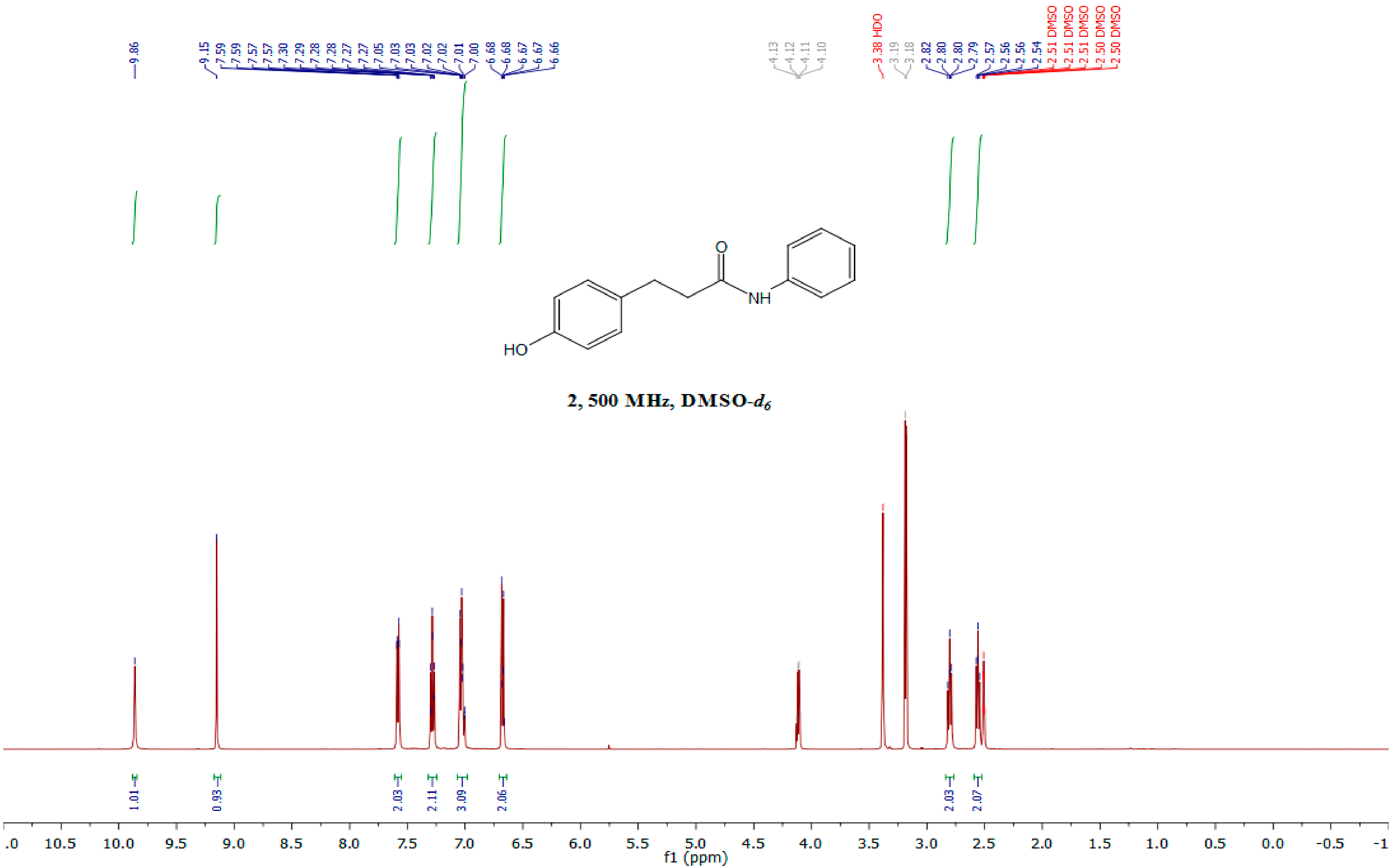

Figure S6.

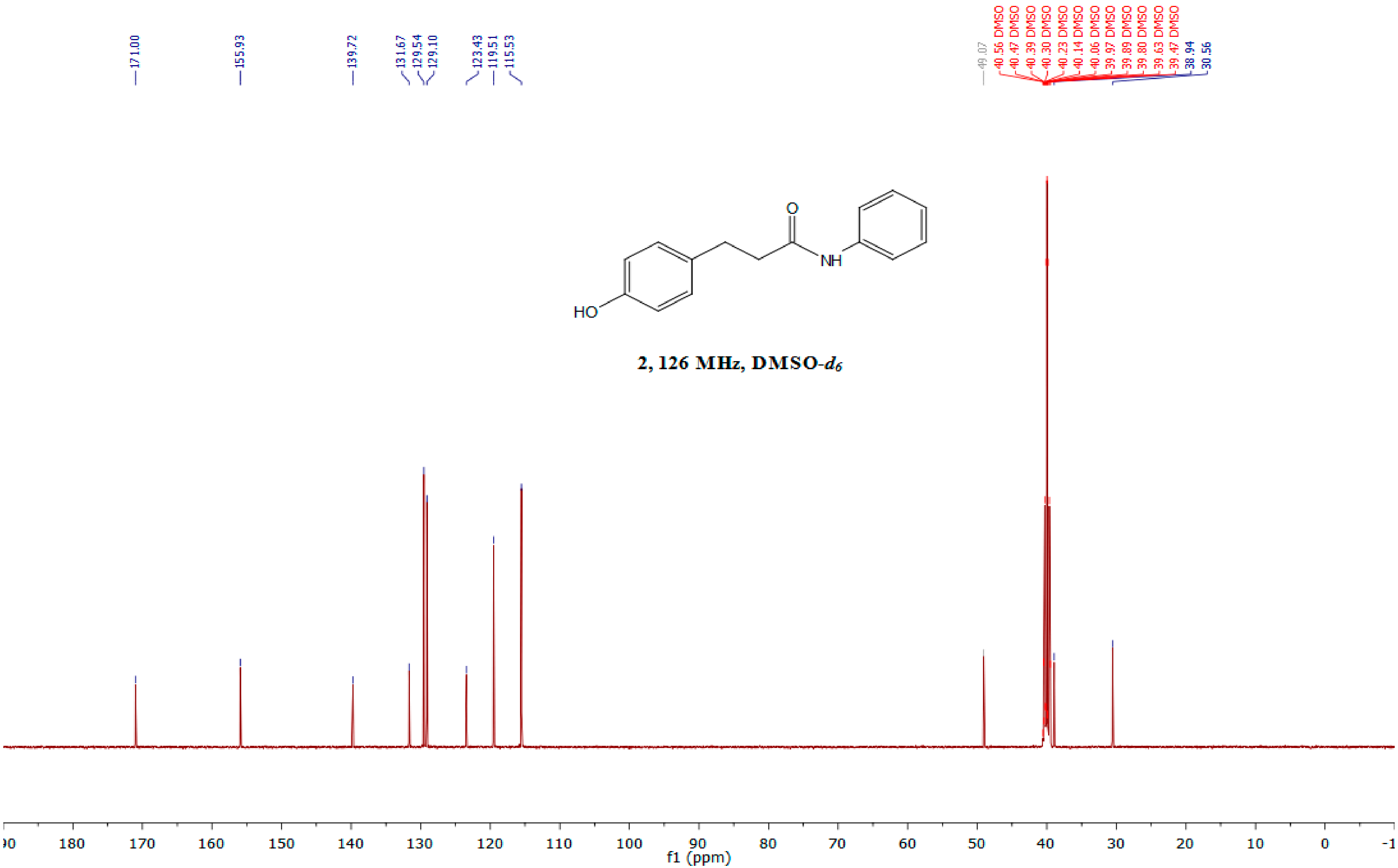
171.00
155.93
139.72
131.67
129.54
129.10
123.43
119.51
115.53
49.07
40.56 DMSO
40.47 DMSO
40.39 DMSO
40.30 DMSO
40.23 DMSO
40.14 DMSO
40.06 DMSO
39.97 DMSO
39.89 DMSO
39.80 DMSO
39.63 DMSO
39.47 DMSO
38.94
30.56
O
NH
HO
2, 126 MHz, DMSO-d6
f1 (ppm)

Figure S7.

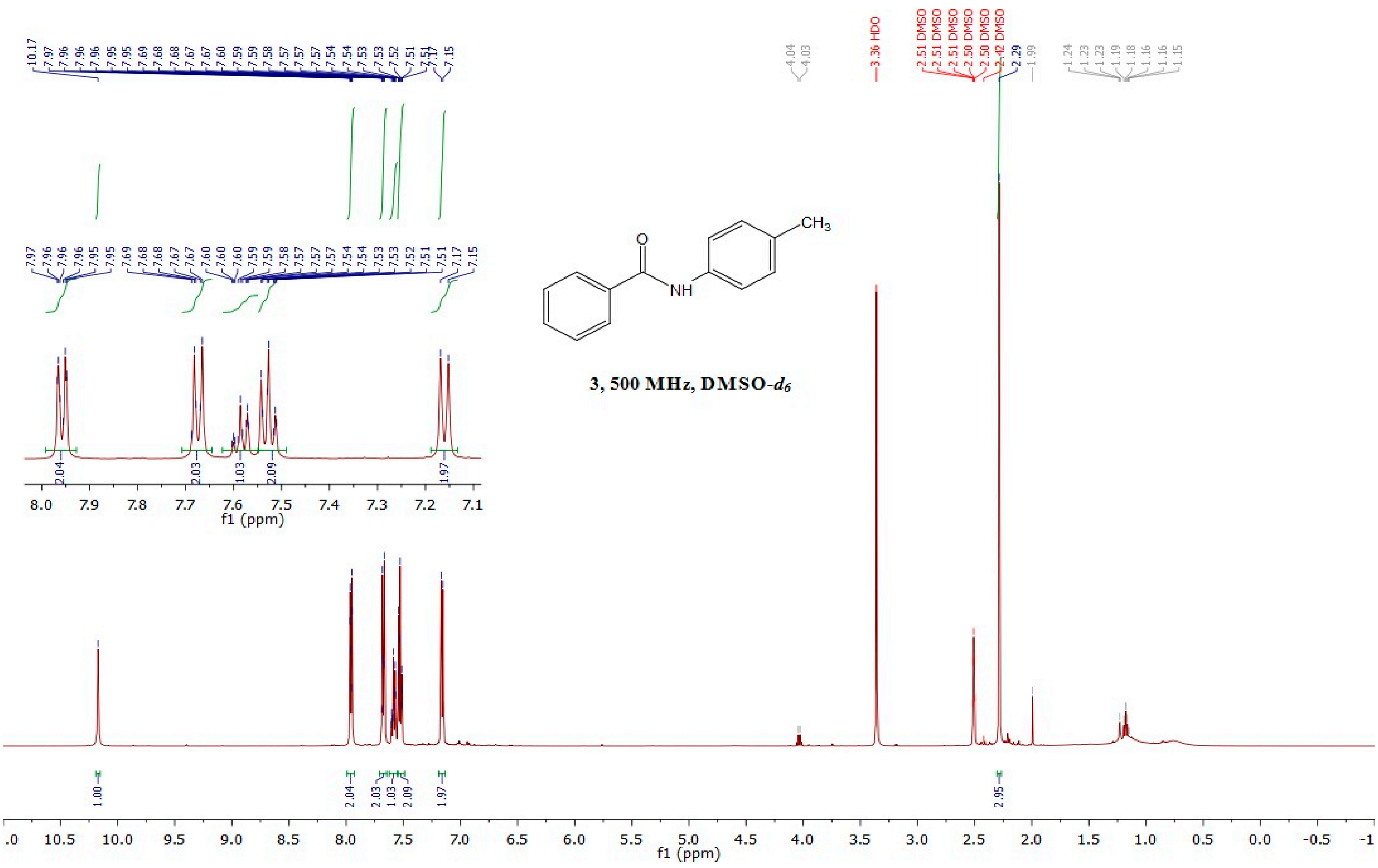

CH3
O
NH
3, 500 MHz, DMSO-d6
3.36 HDO
2.51 DMSO
2.51 DMSO
2.51 DMSO
2.50 DMSO
2.50 DMSO
2.42 DMSO
f1 (ppm)

Figure S8.

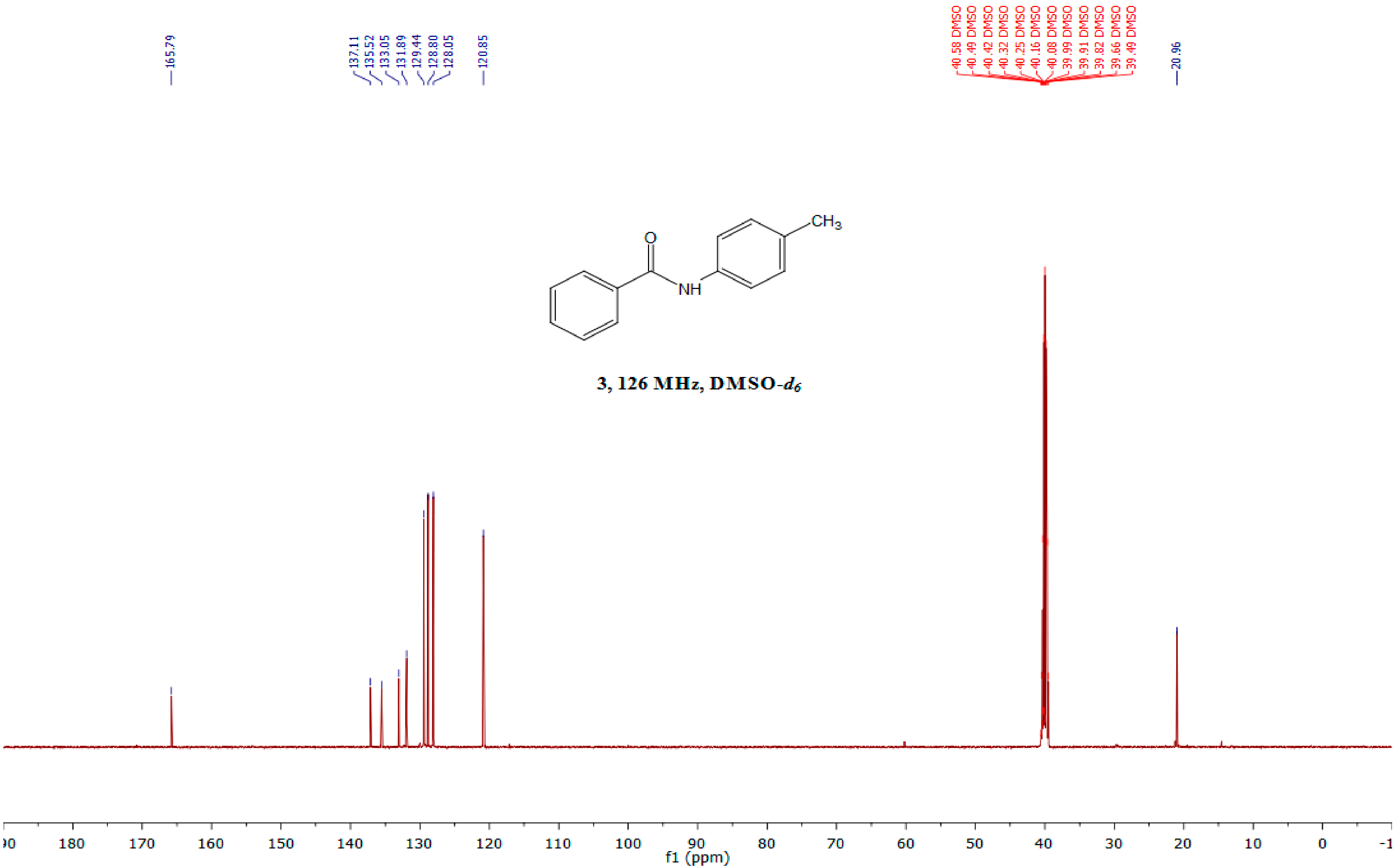
165.79
137.11
135.52
133.05
131.89
129.44
128.80
128.05
120.85
40.58 DMSO
40.49 DMSO
40.42 DMSO
40.32 DMSO
40.25 DMSO
40.16 DMSO
40.08 DMSO
39.99 DMSO
39.91 DMSO
39.82 DMSO
39.66 DMSO
39.49 DMSO
20.96
O
NH
CH3
3, 126 MHz, DMSO-d6
180
170
160
150
140
130
120
110
100
90
80
70
60
50
40
30
20
10
0
f1 (ppm)

Figure S9.

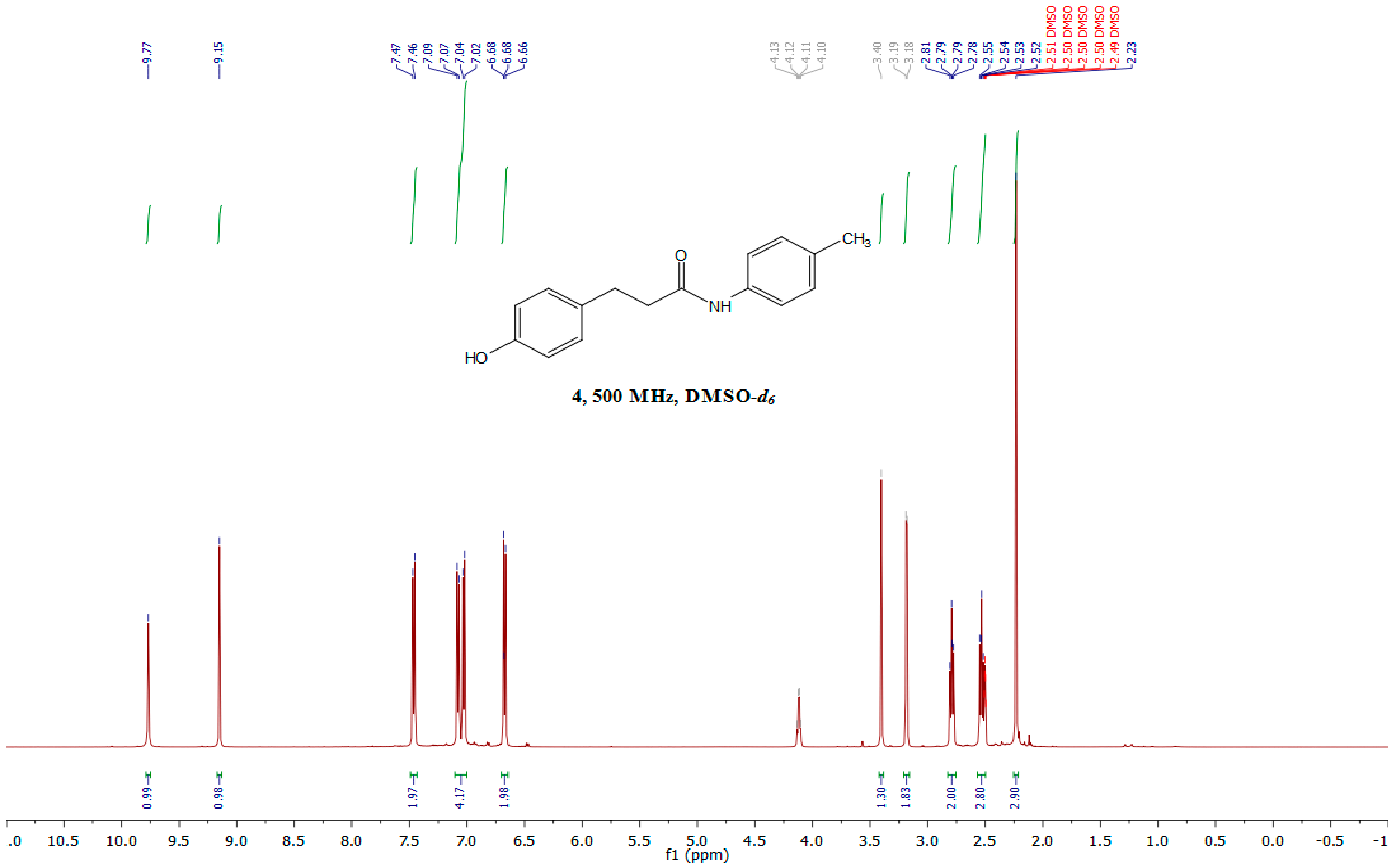

9.77
9.15
7.47
7.46
7.09
7.07
7.04
7.02
6.68
6.68
6.66
4.13
4.12
4.11
4.10
3.40
3.19
3.18
2.81
2.79
2.79
2.78
2.55
2.54
2.53
2.52
2.51 DMSO
2.50 DMSO
2.50 DMSO
2.50 DMSO
2.49 DMSO
2.23
CH3
O
NH
HO
4, 500 MHz, DMSO-d6
0.99
0.98
1.97
4.17
1.98
1.30
1.83
2.00
2.80
2.90
f1 (ppm)

Figure S10.

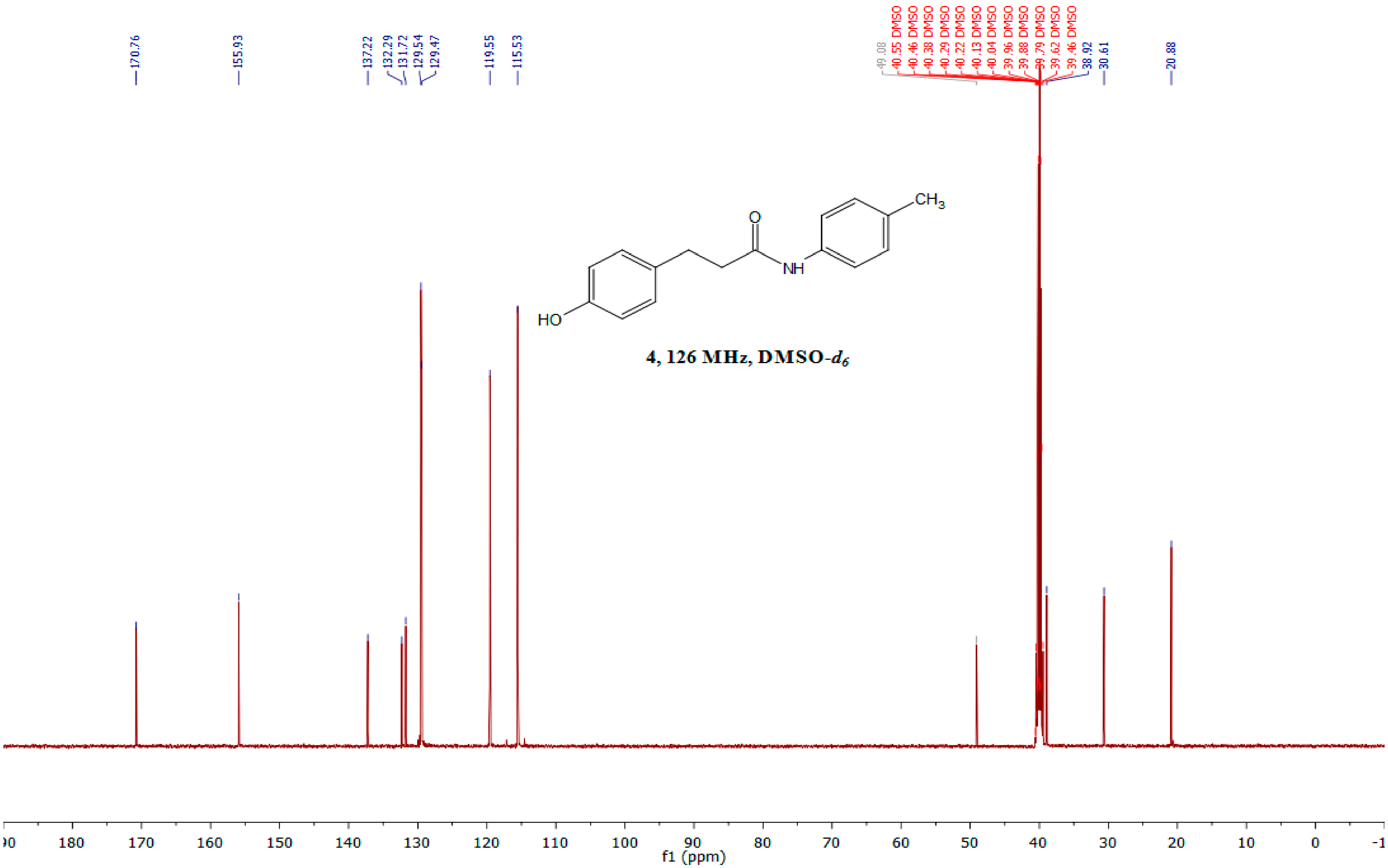
170.76
155.93
137.22
132.29
131.72
129.54
129.47
119.55
115.53
49.08
40.55 DMSO
40.46 DMSO
40.38 DMSO
40.29 DMSO
40.22 DMSO
40.13 DMSO
40.04 DMSO
39.96 DMSO
39.88 DMSO
39.79 DMSO
39.62 DMSO
39.46 DMSO
38.92
30.61
20.88
HO
O
NH
CH3
4, 126 MHz, DMSO-d6
f1 (ppm)

Figure S11.

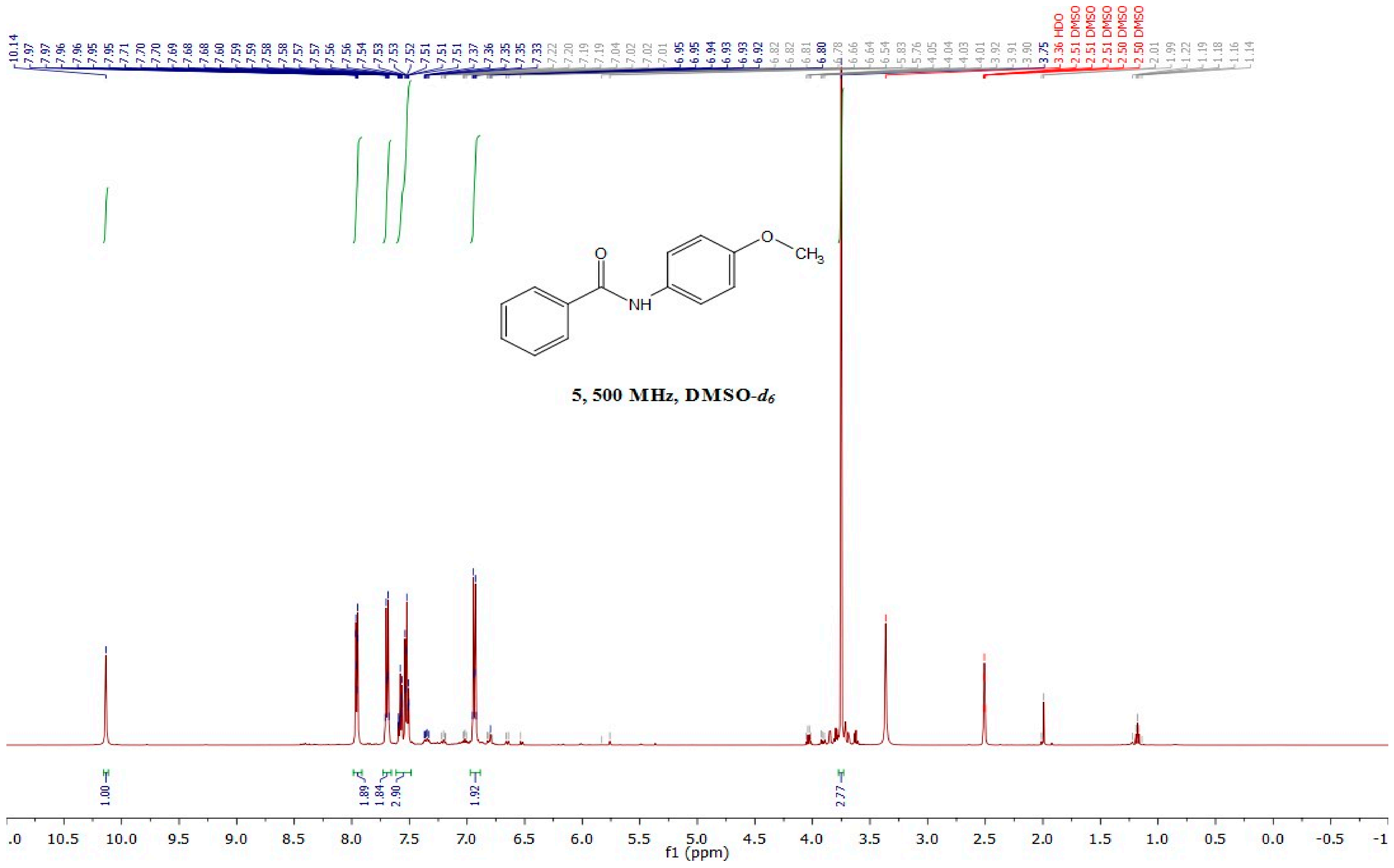

Figure S12.

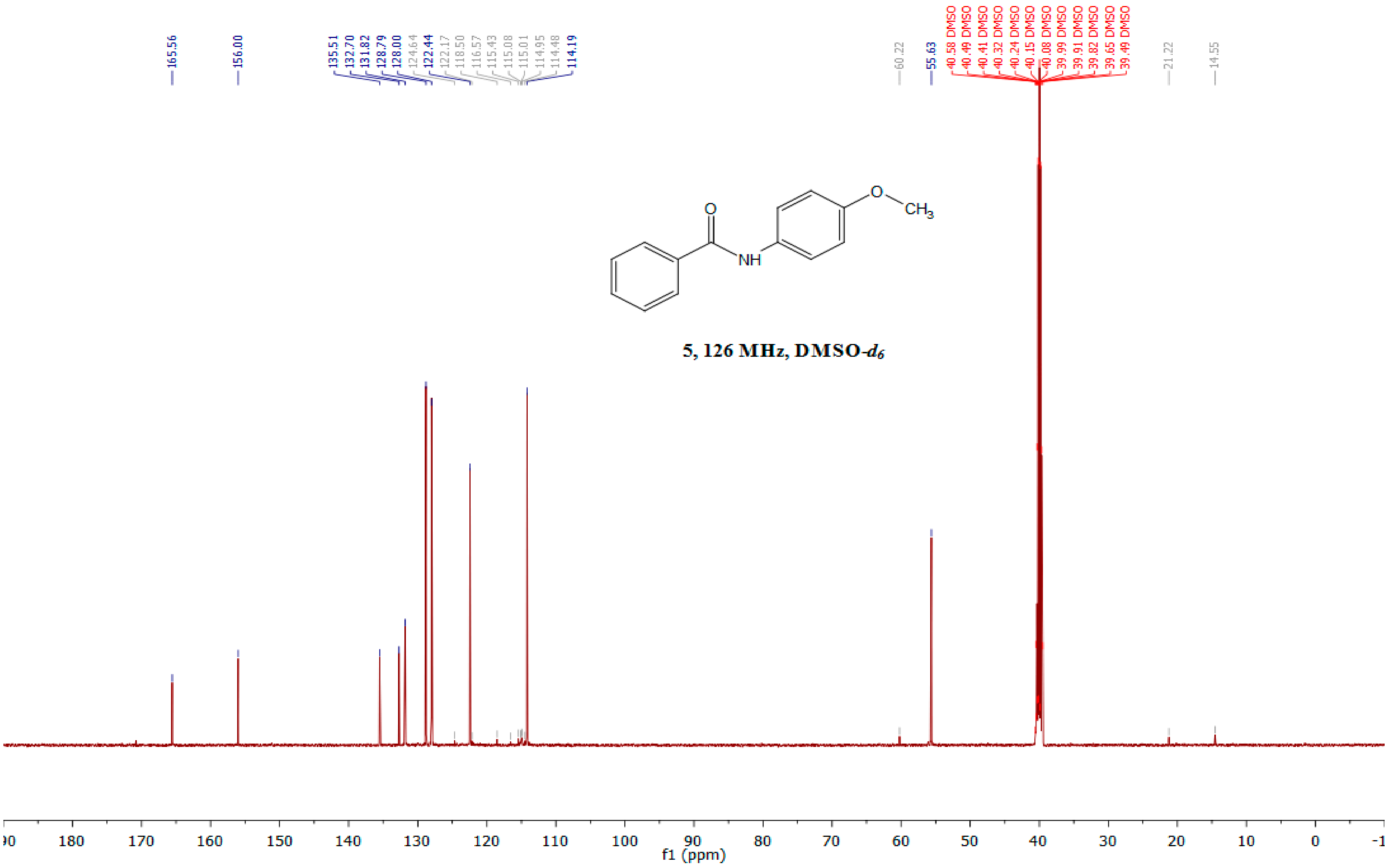

165.56
156.00
135.51
132.70
131.82
128.79
128.00
124.64
122.44
122.17
118.50
116.57
115.43
115.08
115.01
114.95
114.48
114.19
60.22
55.63
40.58 DMSO
40.49 DMSO
40.41 DMSO
40.32 DMSO
40.24 DMSO
40.15 DMSO
40.08 DMSO
39.99 DMSO
39.91 DMSO
39.82 DMSO
39.65 DMSO
39.49 DMSO
21.22
14.55
O
NH
O
CH3
5, 126 MHz, DMSO-d6
f1 (ppm)

Figure S13.

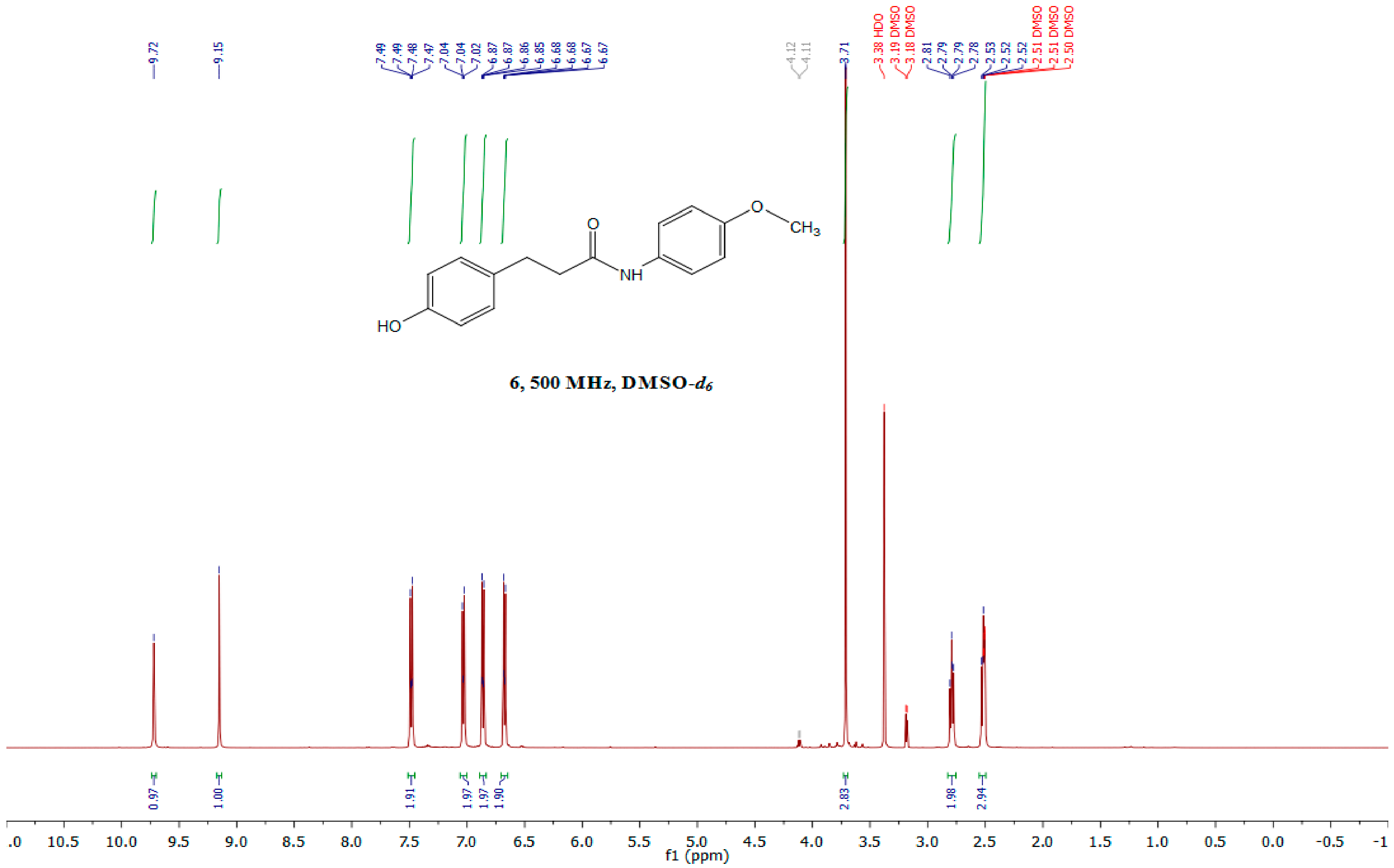

9.72
9.15
7.49
7.49
7.48
7.47
7.04
7.04
7.02
6.87
6.87
6.86
6.85
6.68
6.68
6.67
6.67
4.12
4.11
3.71
3.38 HDO
3.19 DMSO
3.18 DMSO
2.81
2.79
2.79
2.78
2.53
2.52
2.52
2.51 DMSO
2.51 DMSO
2.50 DMSO
O
NH
O
CH3
HO
6, 500 MHz, DMSO-d6
0.97
1.00
1.91
1.97
1.97
1.90
2.83
1.98
2.94
f1 (ppm)

Figure S14.

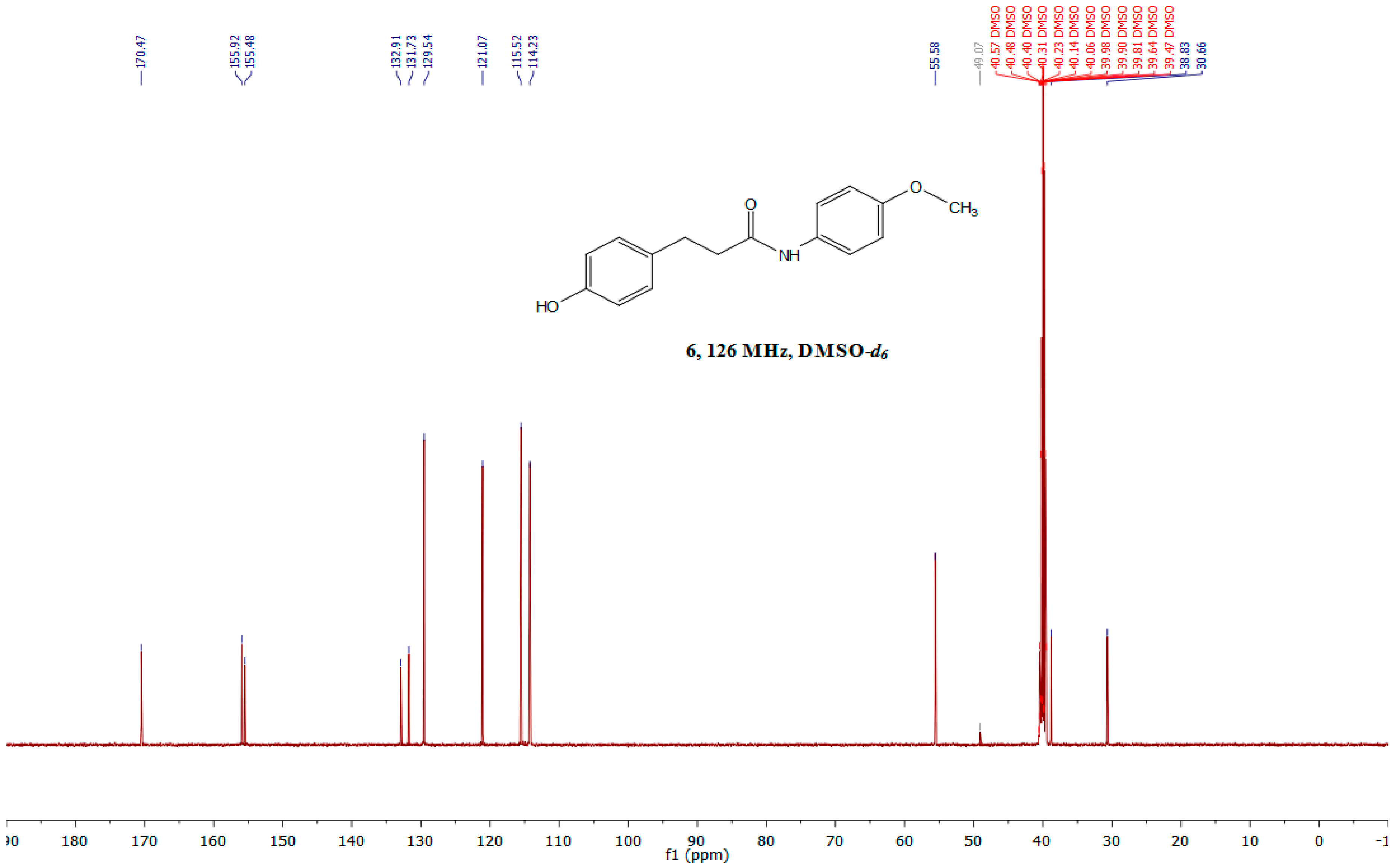
170.47
155.92
155.48
132.91
131.73
129.54
121.07
115.52
114.23
55.58
49.07
40.57 DMSO
40.48 DMSO
40.40 DMSO
40.31 DMSO
40.23 DMSO
40.14 DMSO
40.06 DMSO
39.98 DMSO
39.90 DMSO
39.81 DMSO
39.64 DMSO
39.47 DMSO
38.83
30.66
O
NH
O
CH3
HO
6, 126 MHz, DMSO-d6
f1 (ppm)

Figure S15.

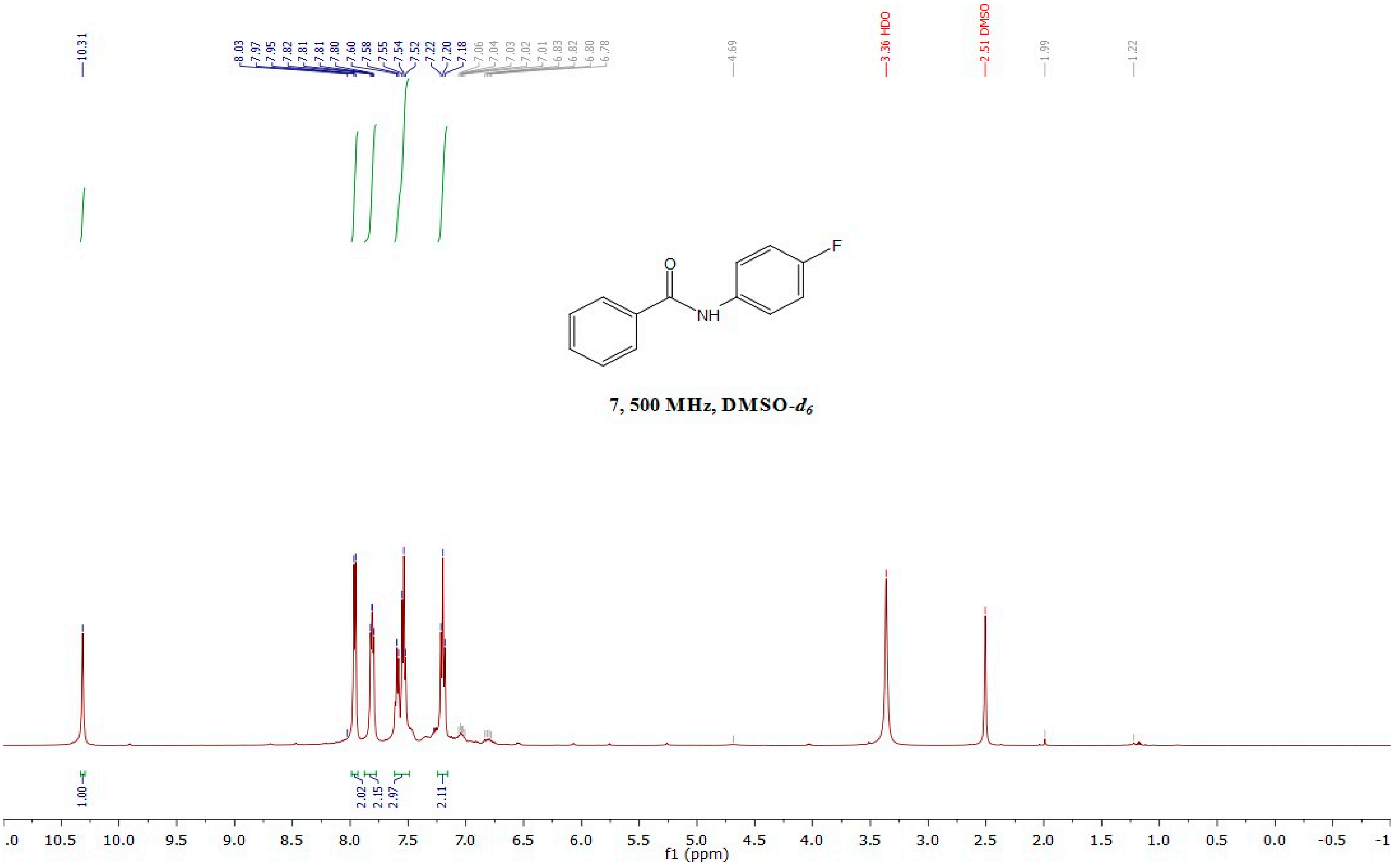

7, 500 MHz, DMSO-d_6

Figure S16.

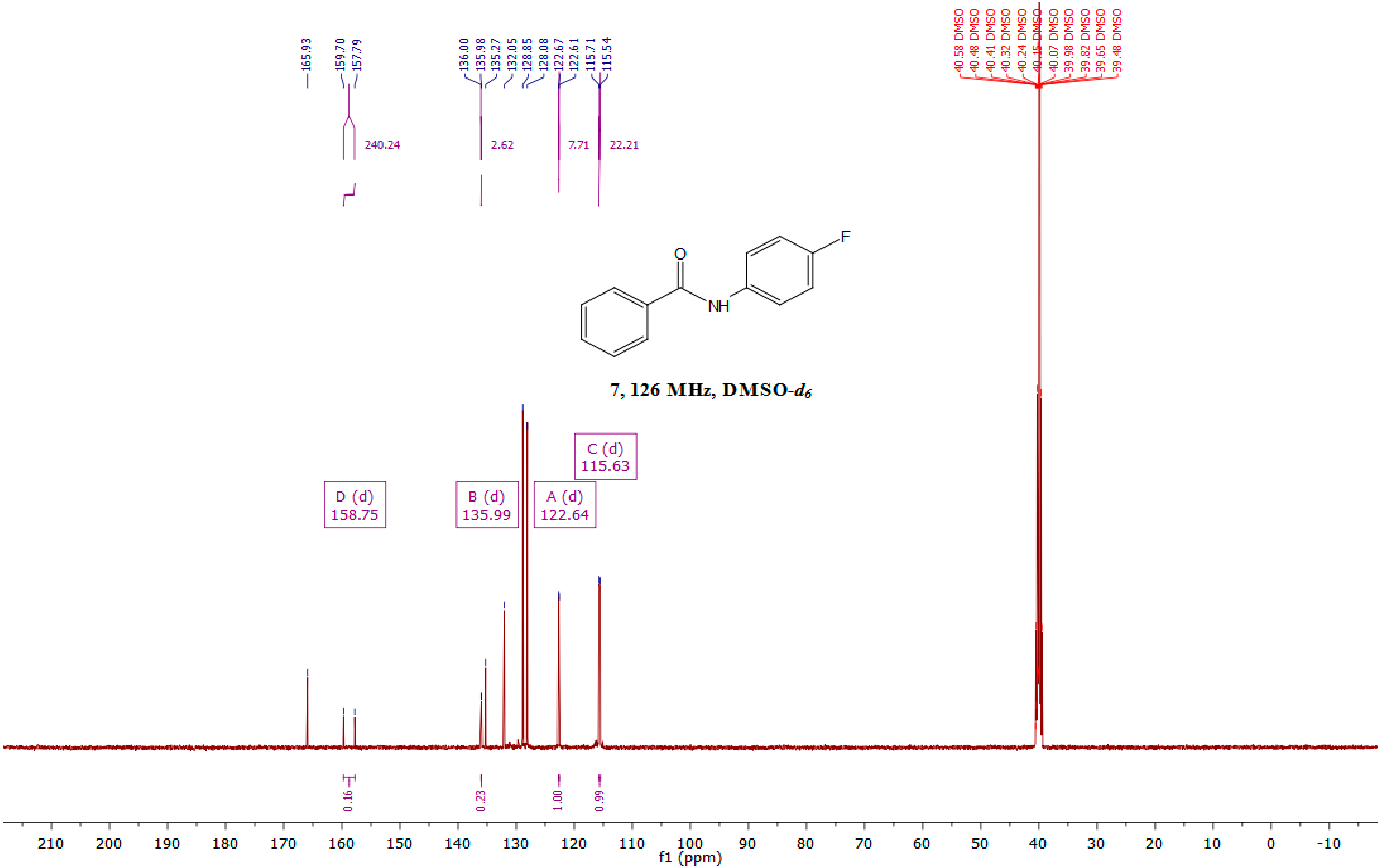

7, 126 MHz, DMSO-*d*$_6$

Figure S17.

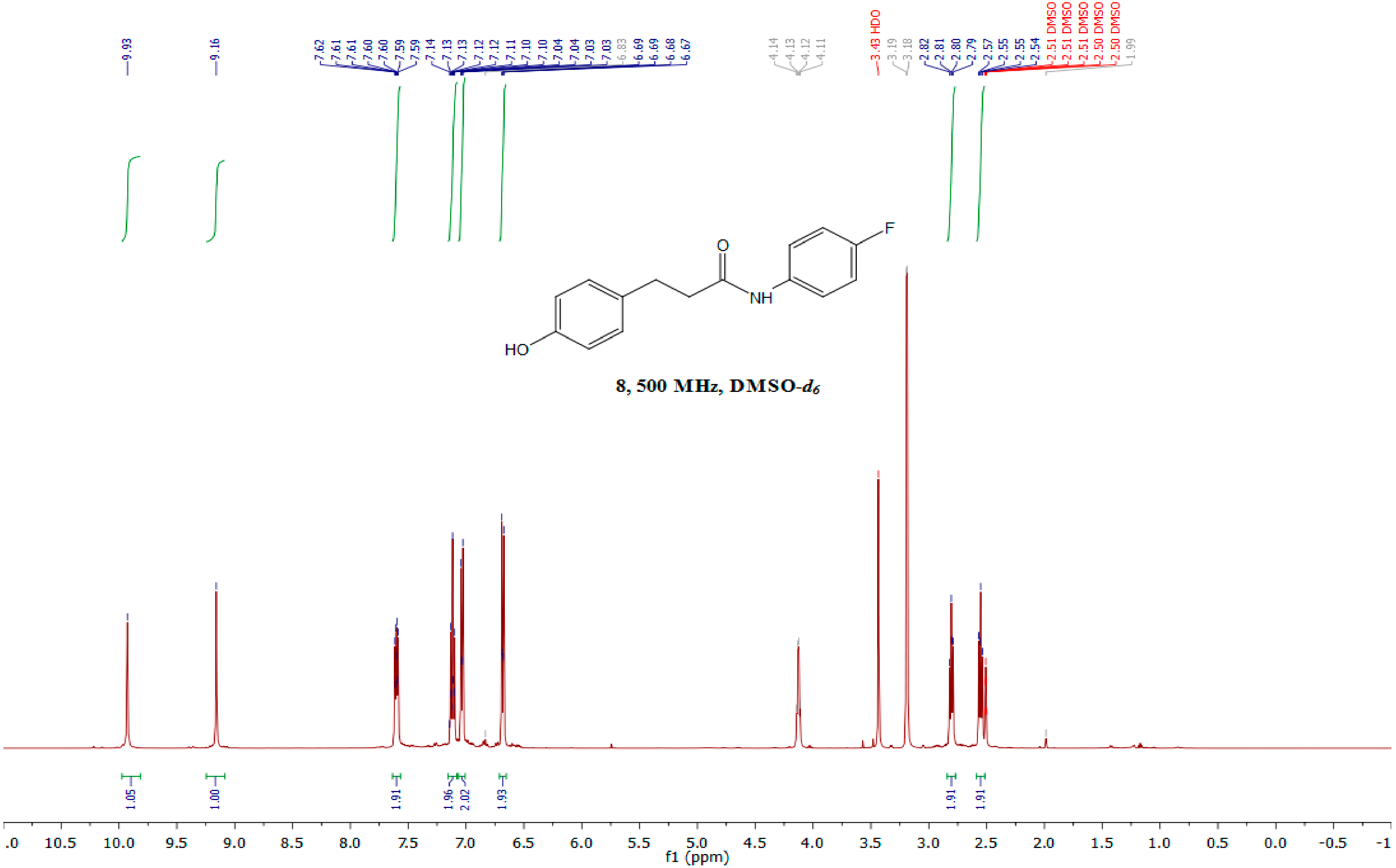

Figure S18.

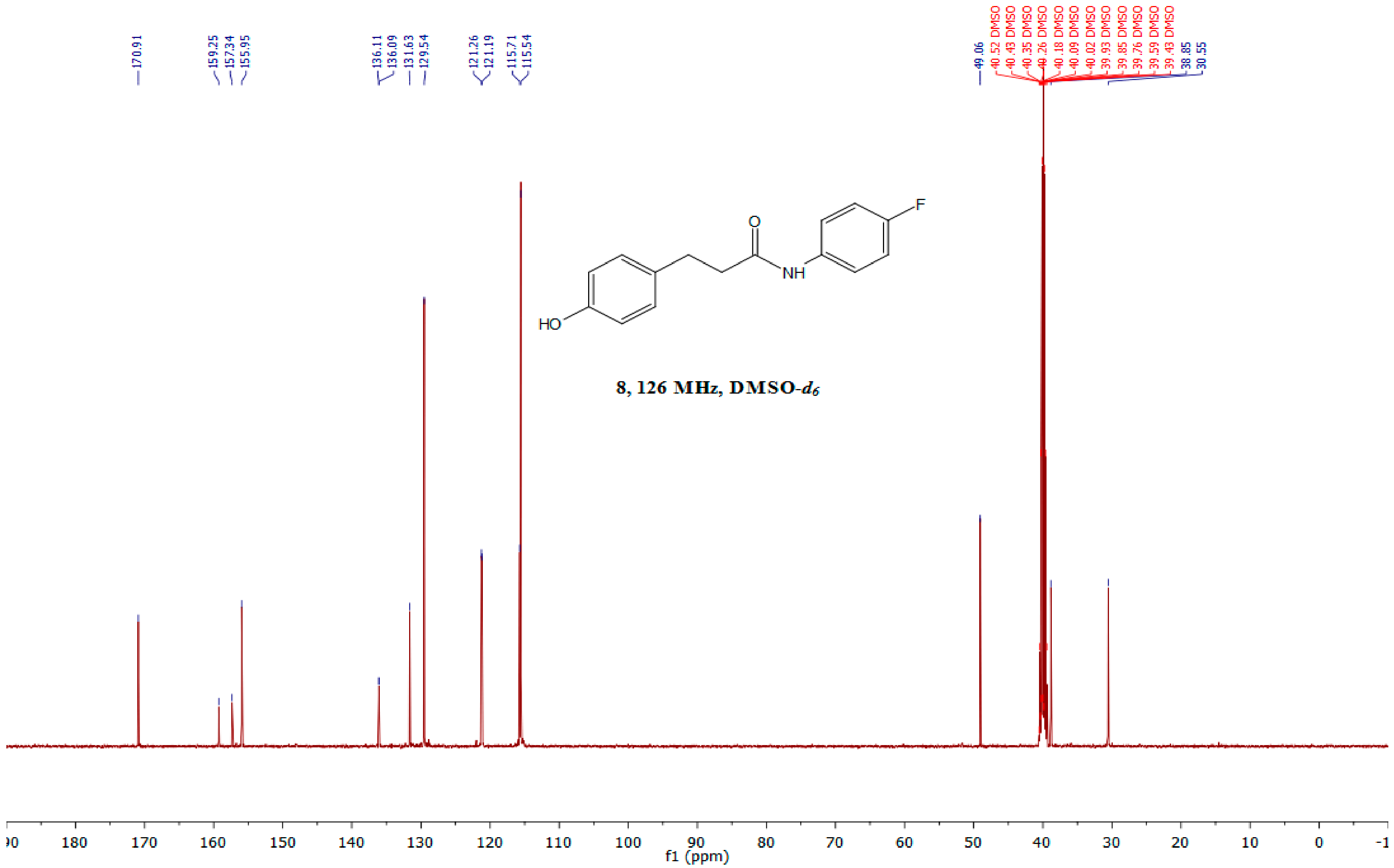

170.91
159.25
157.34
155.95
136.11
136.09
131.63
129.54
121.26
121.19
115.71
115.54
49.06
40.52 DMSO
40.43 DMSO
40.35 DMSO
40.26 DMSO
40.18 DMSO
40.09 DMSO
40.02 DMSO
39.93 DMSO
39.85 DMSO
39.76 DMSO
39.59 DMSO
39.43 DMSO
38.85
30.55
O
F
NH
HO
8, 126 MHz, DMSO-d_6
180
170
160
150
140
130
120
110
100
90
80
70
60
50
40
30
20
10
0
f1 (ppm)

Figure S19.

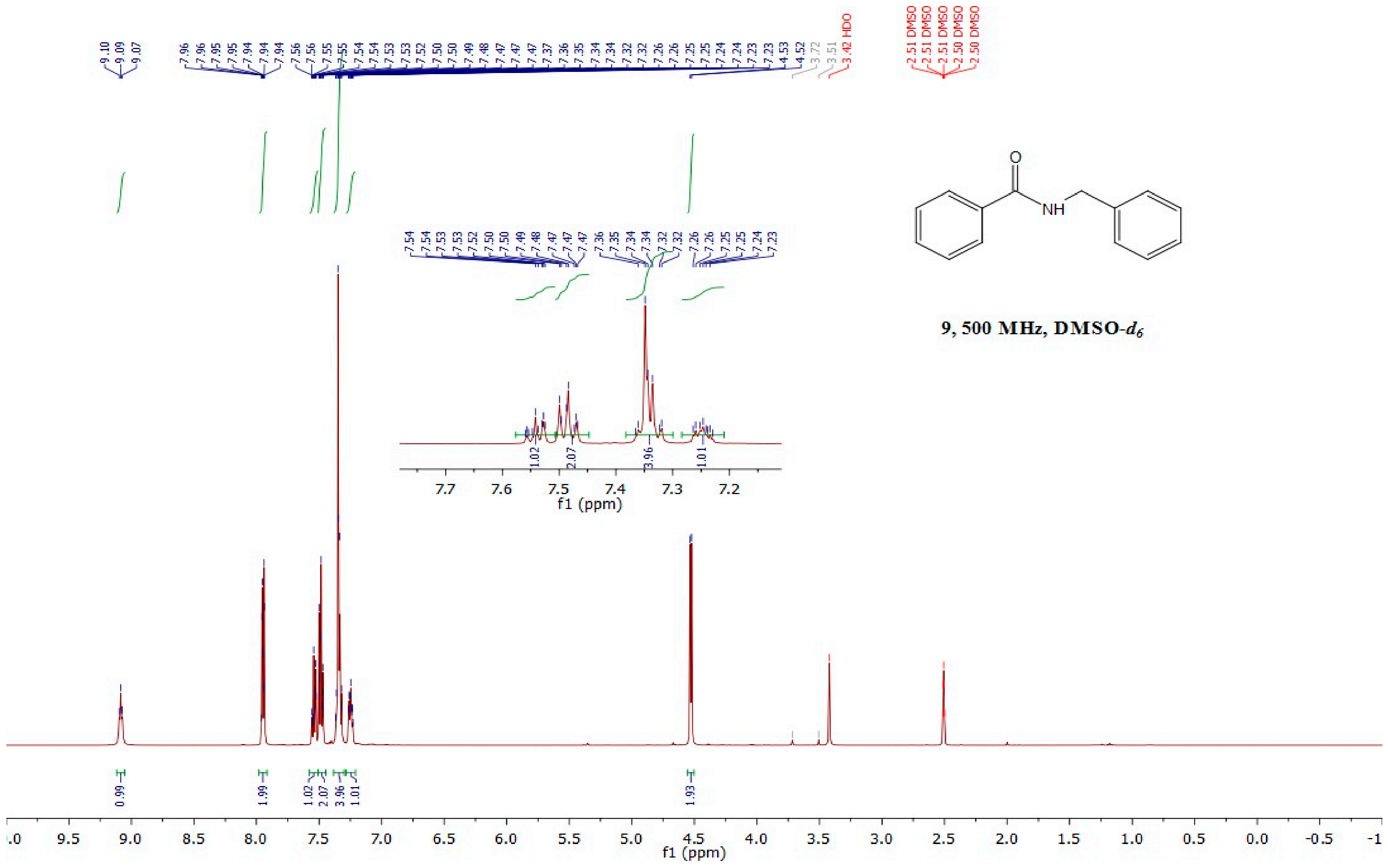

Figure S20.

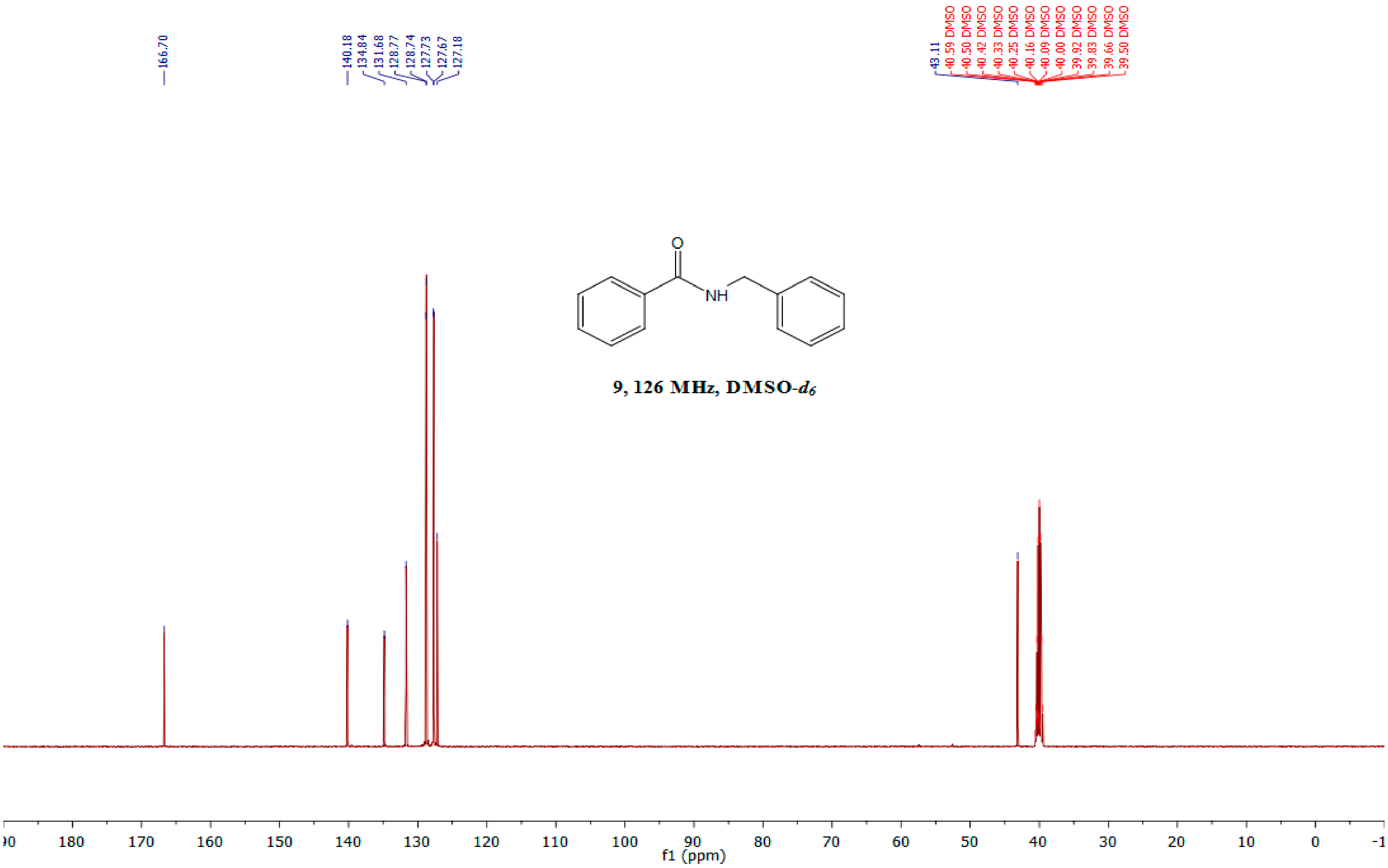
166.70
140.18
134.84
131.68
128.77
128.74
127.73
127.67
127.18
43.11
40.59 DMSO
40.50 DMSO
40.42 DMSO
40.33 DMSO
40.25 DMSO
40.16 DMSO
40.09 DMSO
40.00 DMSO
39.92 DMSO
39.83 DMSO
39.66 DMSO
39.50 DMSO
O
NH
9, 126 MHz, DMSO-d_6
f1 (ppm)

Figure S21.

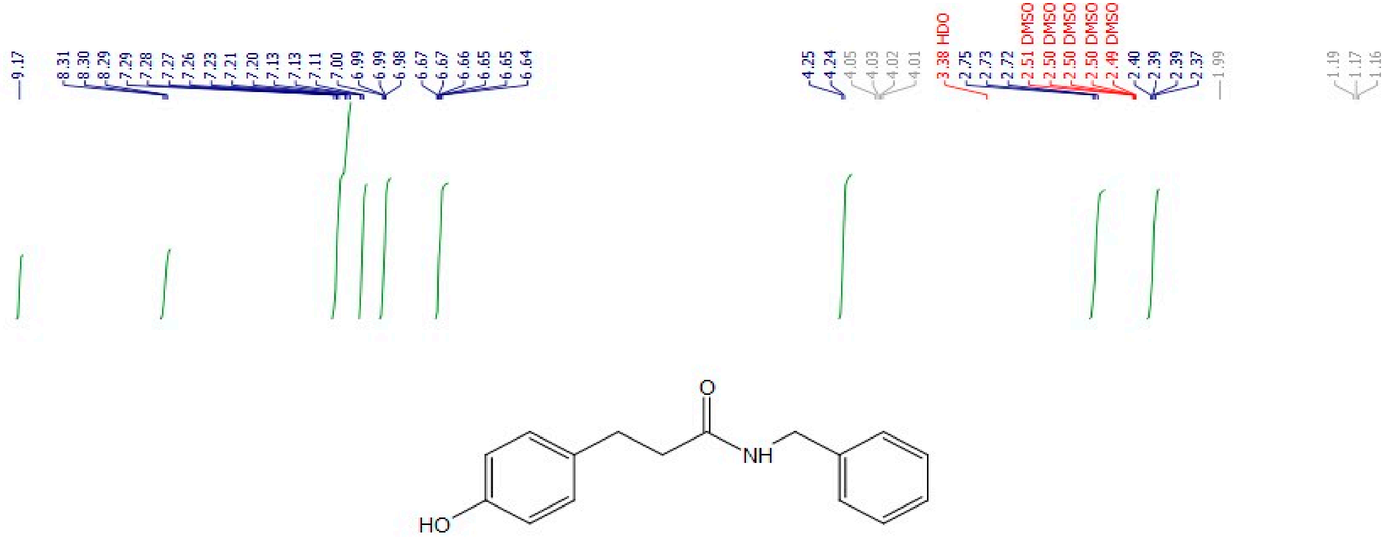

10, 500 MHz, DMSO-d_6

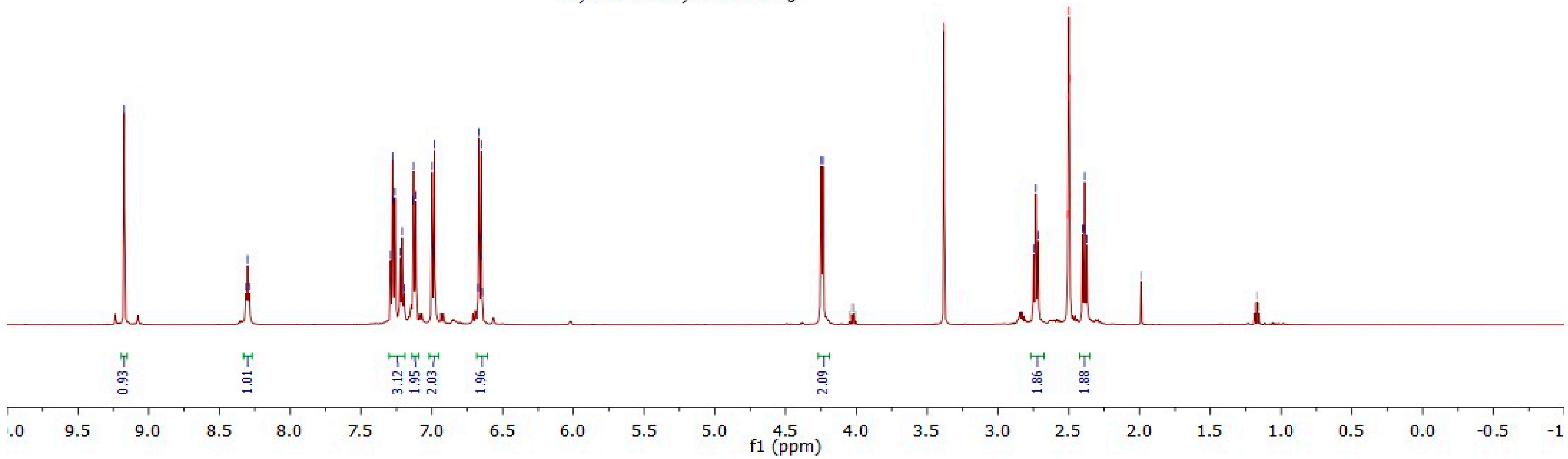

Figure S22.

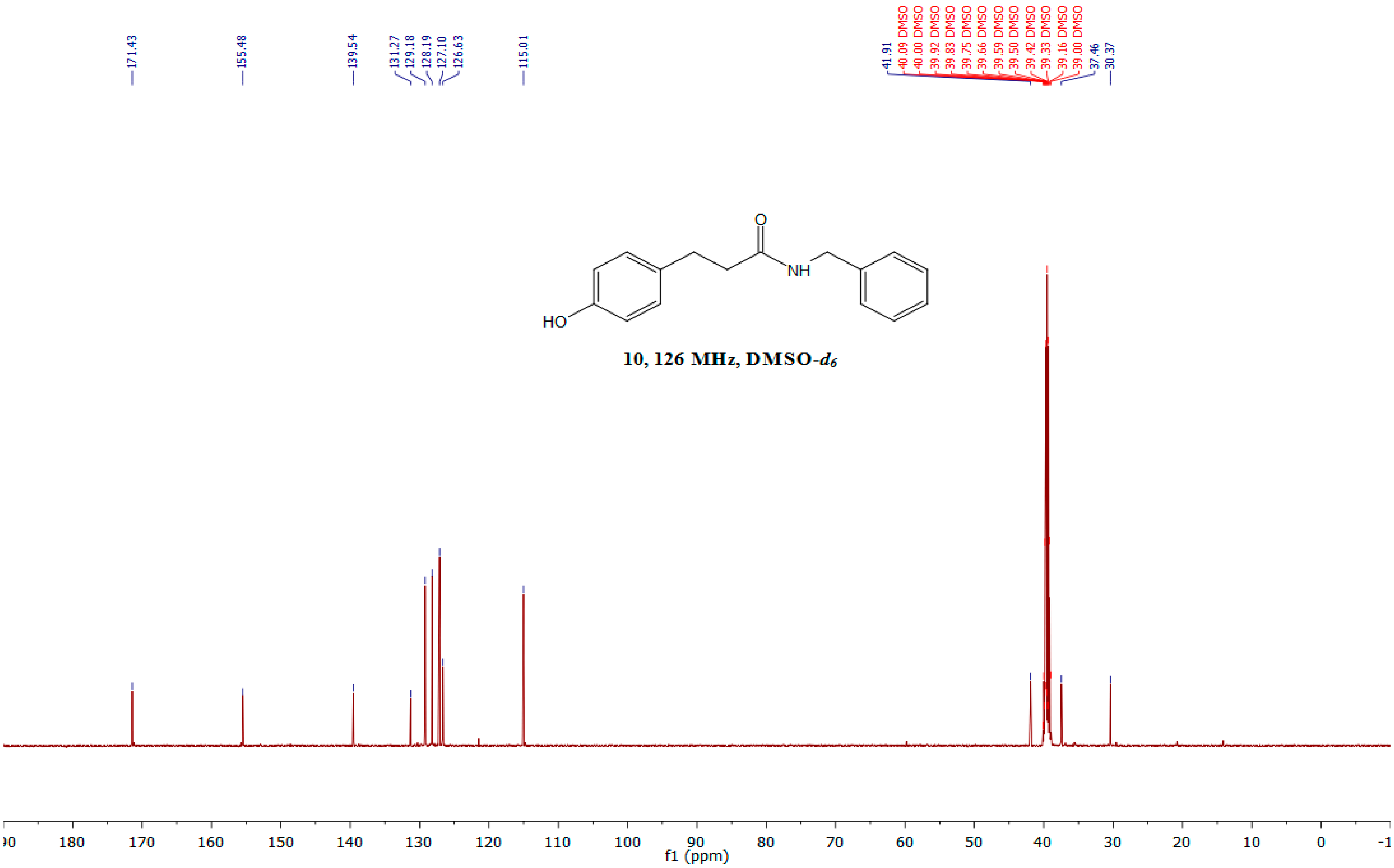
171.43
155.48
139.54
131.27
129.18
128.19
127.10
126.63
115.01
41.91
40.09 DMSO
40.00 DMSO
39.92 DMSO
39.83 DMSO
39.75 DMSO
39.66 DMSO
39.59 DMSO
39.50 DMSO
39.42 DMSO
39.33 DMSO
39.16 DMSO
39.00 DMSO
37.46
30.37
O
NH
HO
10, 126 MHz, DMSO-d6
f1 (ppm)

Figure S23.

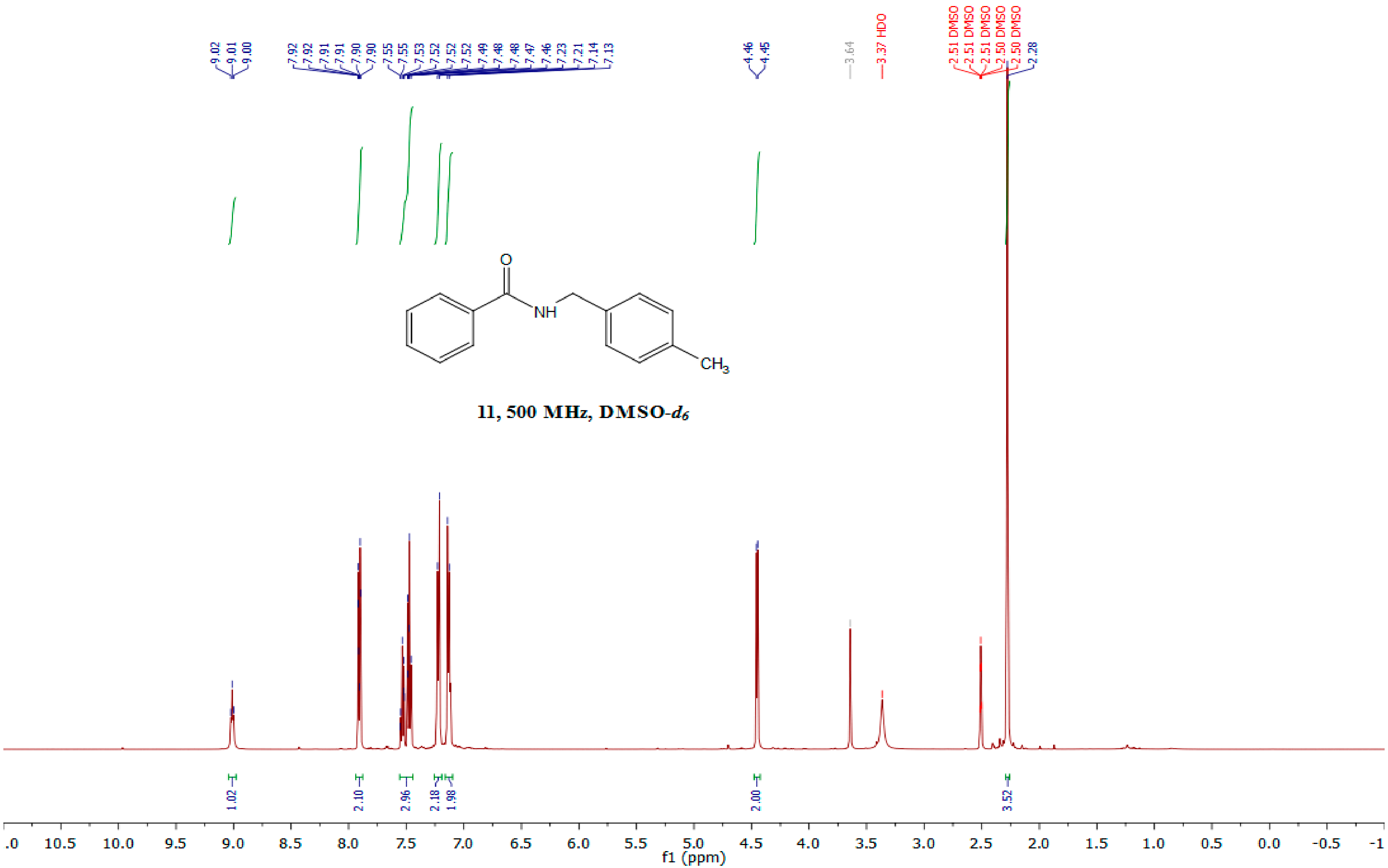
9.02
9.01
9.00
7.92
7.92
7.91
7.91
7.90
7.90
7.55
7.55
7.53
7.52
7.52
7.52
7.49
7.48
7.48
7.47
7.46
7.23
7.21
7.14
7.13
4.46
4.45
3.64
3.37 HDO
2.51 DMSO
2.51 DMSO
2.51 DMSO
2.50 DMSO
2.50 DMSO
2.28
O
NH
CH3
11, 500 MHz, DMSO-d6
1.02
2.10
2.96
2.18
1.98
2.00
3.52
f1 (ppm)

Figure S24.

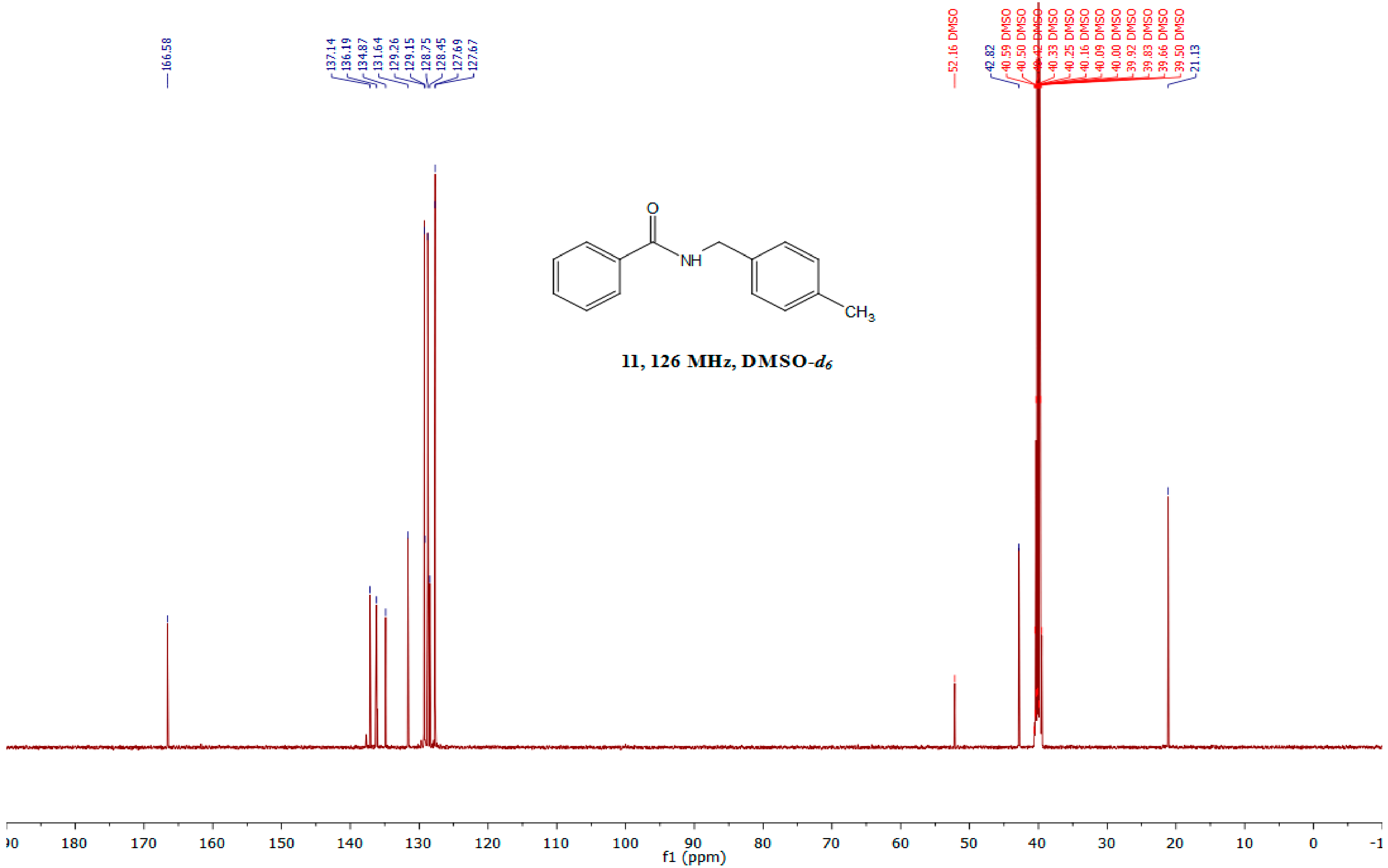

166.58
137.14
136.19
134.87
131.64
129.26
129.15
128.75
128.45
127.69
127.67
52.16 DMSO
42.82
40.59 DMSO
40.50 DMSO
40.42 DMSO
40.33 DMSO
40.25 DMSO
40.16 DMSO
40.09 DMSO
40.00 DMSO
39.92 DMSO
39.83 DMSO
39.66 DMSO
39.50 DMSO
21.13
O
NH
CH_3
11, 126 MHz, DMSO-d_6
f1 (ppm)

Figure S25.

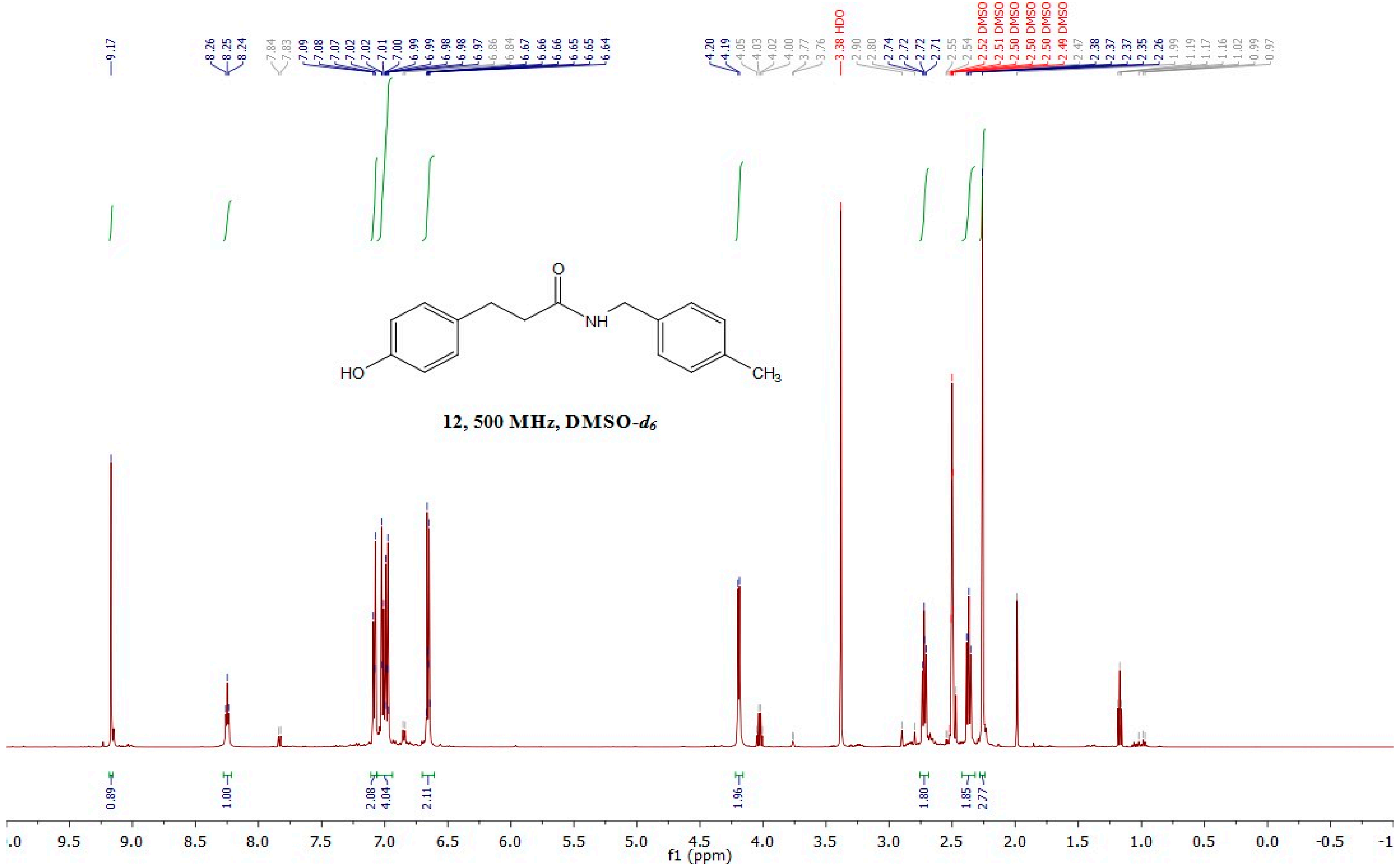
12, 500 MHz, DMSO-d6
HO
NH
O
CH3
f1 (ppm)
9.17
8.26
8.25
8.24
7.84
7.83
7.09
7.08
7.07
7.02
7.02
7.01
7.00
6.99
6.99
6.98
6.98
6.97
6.86
6.84
6.67
6.66
6.66
6.65
6.65
6.64
4.20
4.19
4.05
4.03
4.02
4.00
3.77
3.76
3.38 HDO
2.90
2.80
2.74
2.72
2.72
2.71
2.55
2.54
2.52 DMSO
2.51 DMSO
2.50 DMSO
2.50 DMSO
2.50 DMSO
2.49 DMSO
2.47
2.38
2.37
2.37
2.35
2.26
1.99
1.19
1.17
1.16
1.02
0.99
0.97
0.89
1.00
2.08
4.04
2.11
1.96
1.80
1.85
2.77

Figure S26.

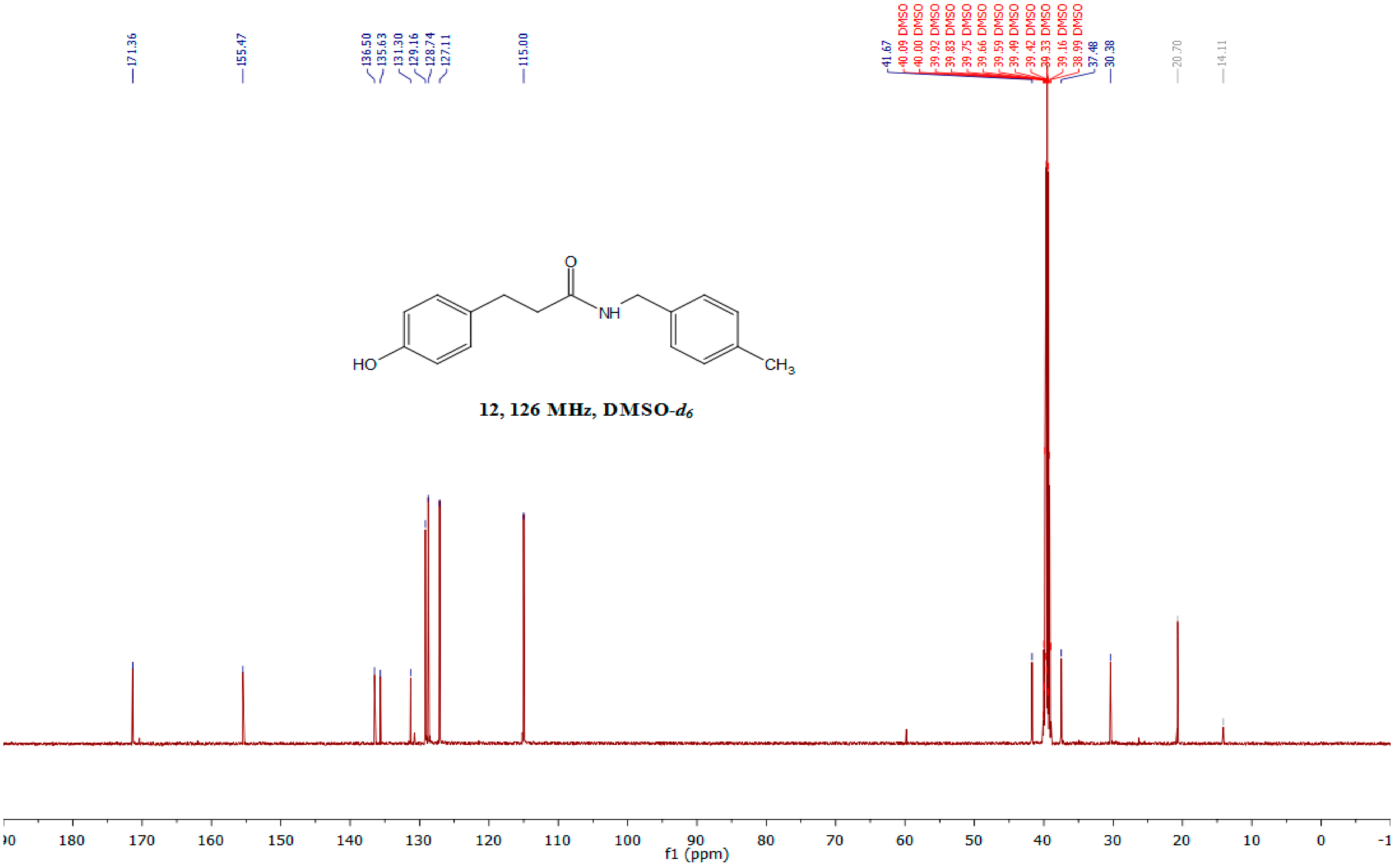

12, 126 MHz, DMSO-d_6

Figure S27.

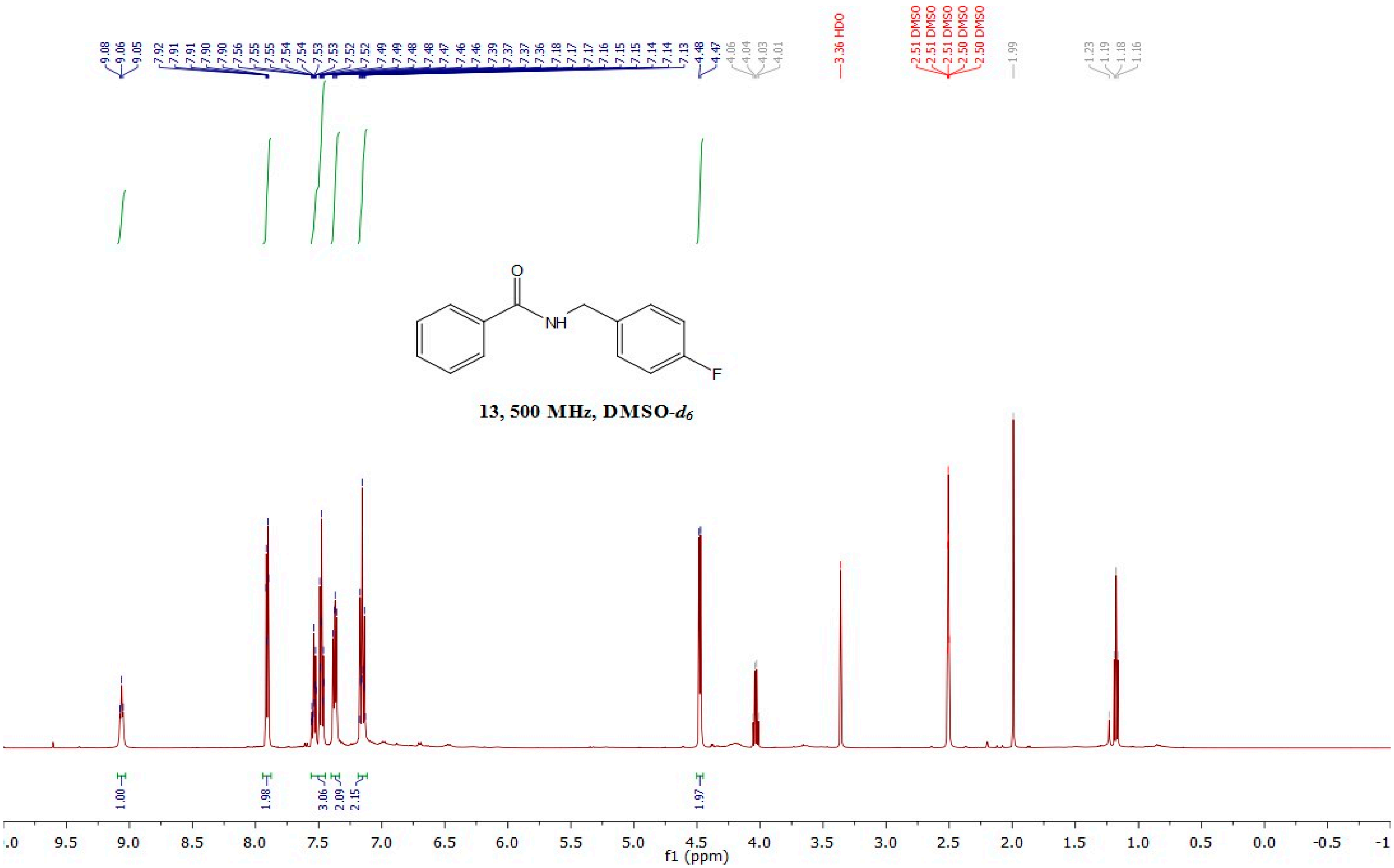

13, 500 MHz, DMSO-d_6

Figure S28.

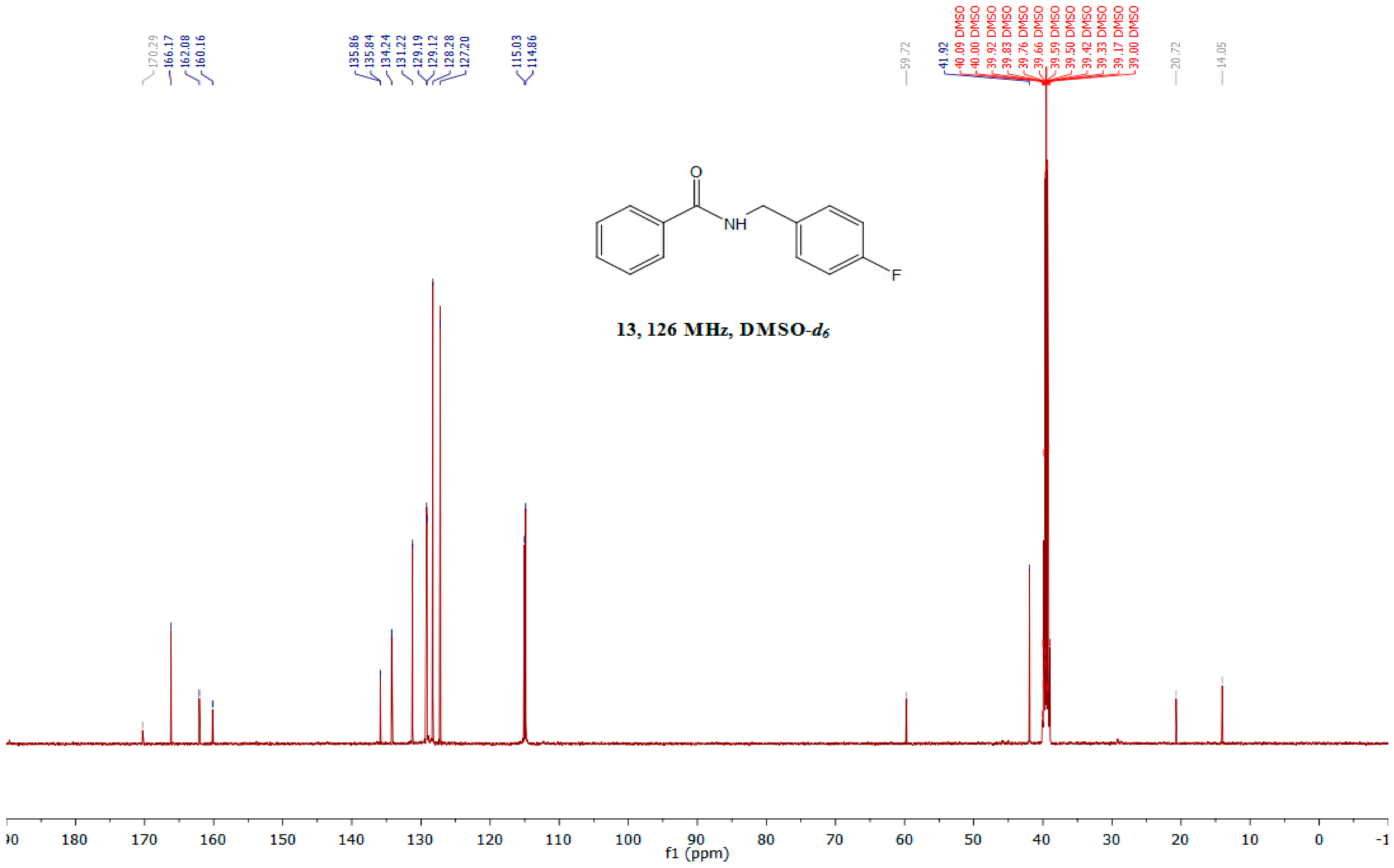
170.29
166.17
162.08
160.16
135.86
135.84
134.24
131.22
129.19
129.12
128.28
127.20
115.03
114.86
59.72
41.92
40.09 DMSO
40.00 DMSO
39.92 DMSO
39.83 DMSO
39.76 DMSO
39.66 DMSO
39.59 DMSO
39.50 DMSO
39.42 DMSO
39.33 DMSO
39.17 DMSO
39.00 DMSO
20.72
14.05
O
NH
F
13, 126 MHz, DMSO-d6
f1 (ppm)

Figure S29.

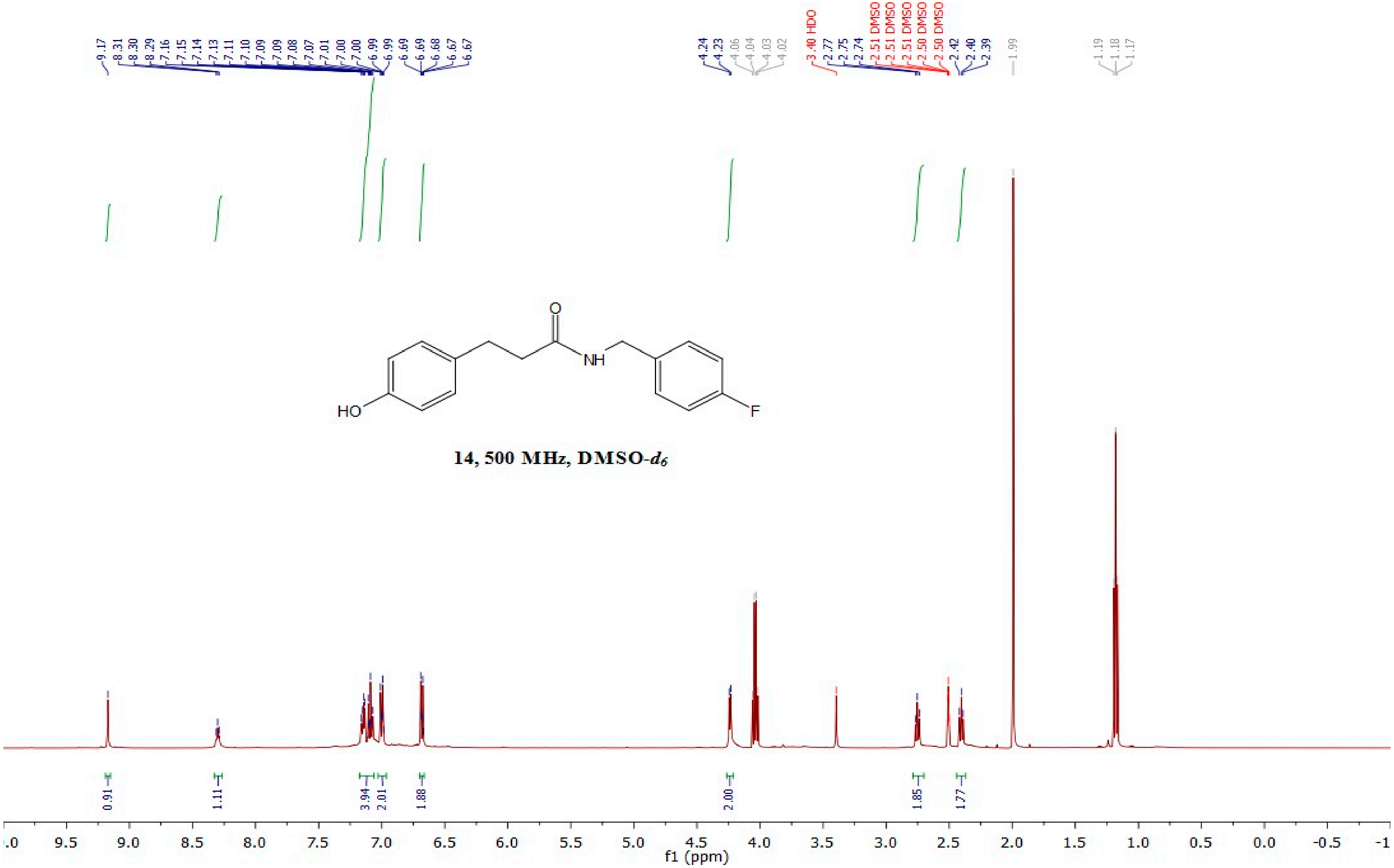

14, 500 MHz, DMSO-d_6

Figure S30.

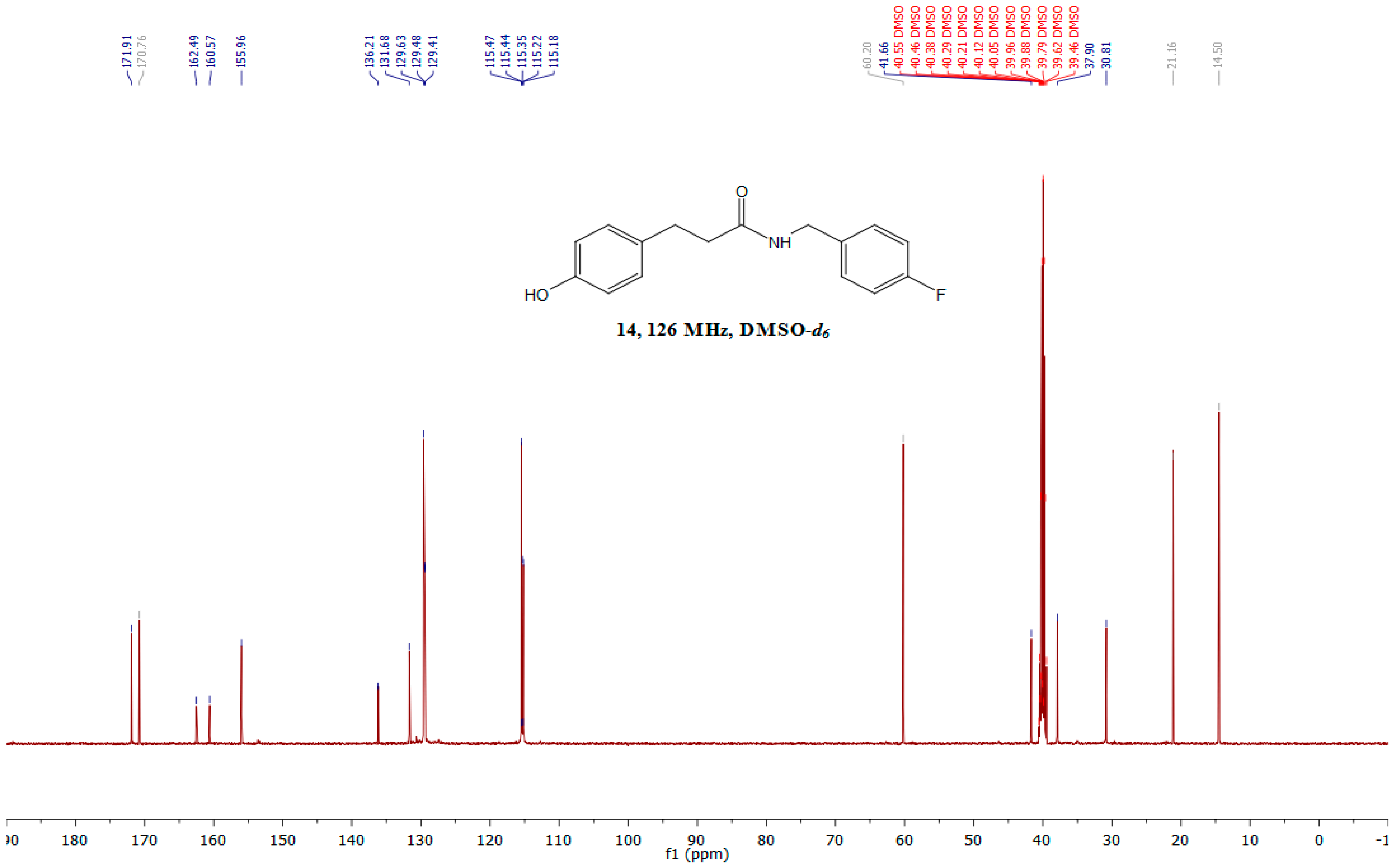
171.91
170.76
162.49
160.57
155.96
136.21
131.68
129.63
129.48
129.41
115.47
115.44
115.35
115.22
115.18
60.20
41.66
40.55 DMSO
40.46 DMSO
40.38 DMSO
40.29 DMSO
40.21 DMSO
40.12 DMSO
40.05 DMSO
39.96 DMSO
39.88 DMSO
39.79 DMSO
39.62 DMSO
39.46 DMSO
37.90
30.81
21.16
14.50
O
NH
HO
F
14, 126 MHz, DMSO-d_6
f1 (ppm)

Figure S31.

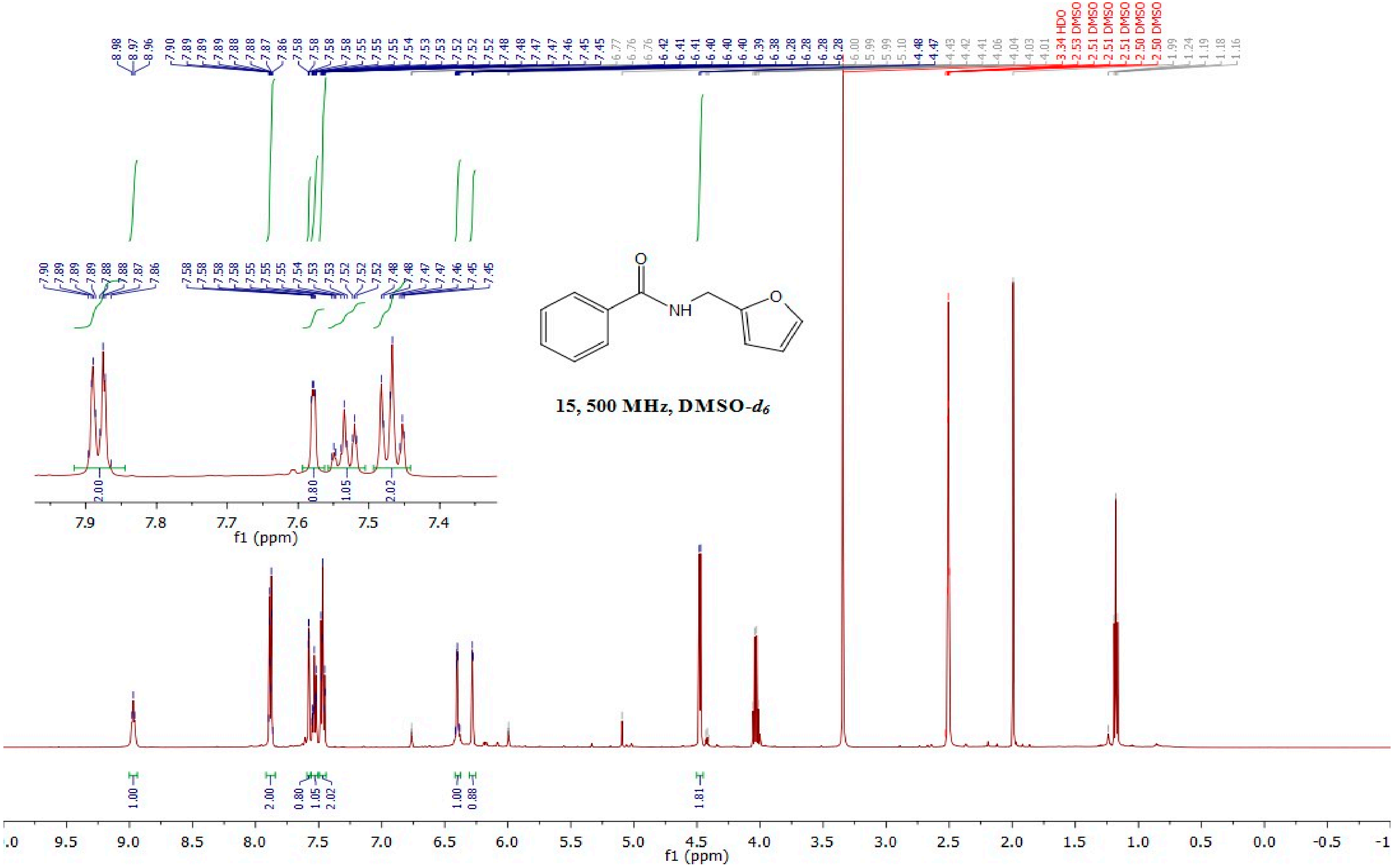

Figure S32.

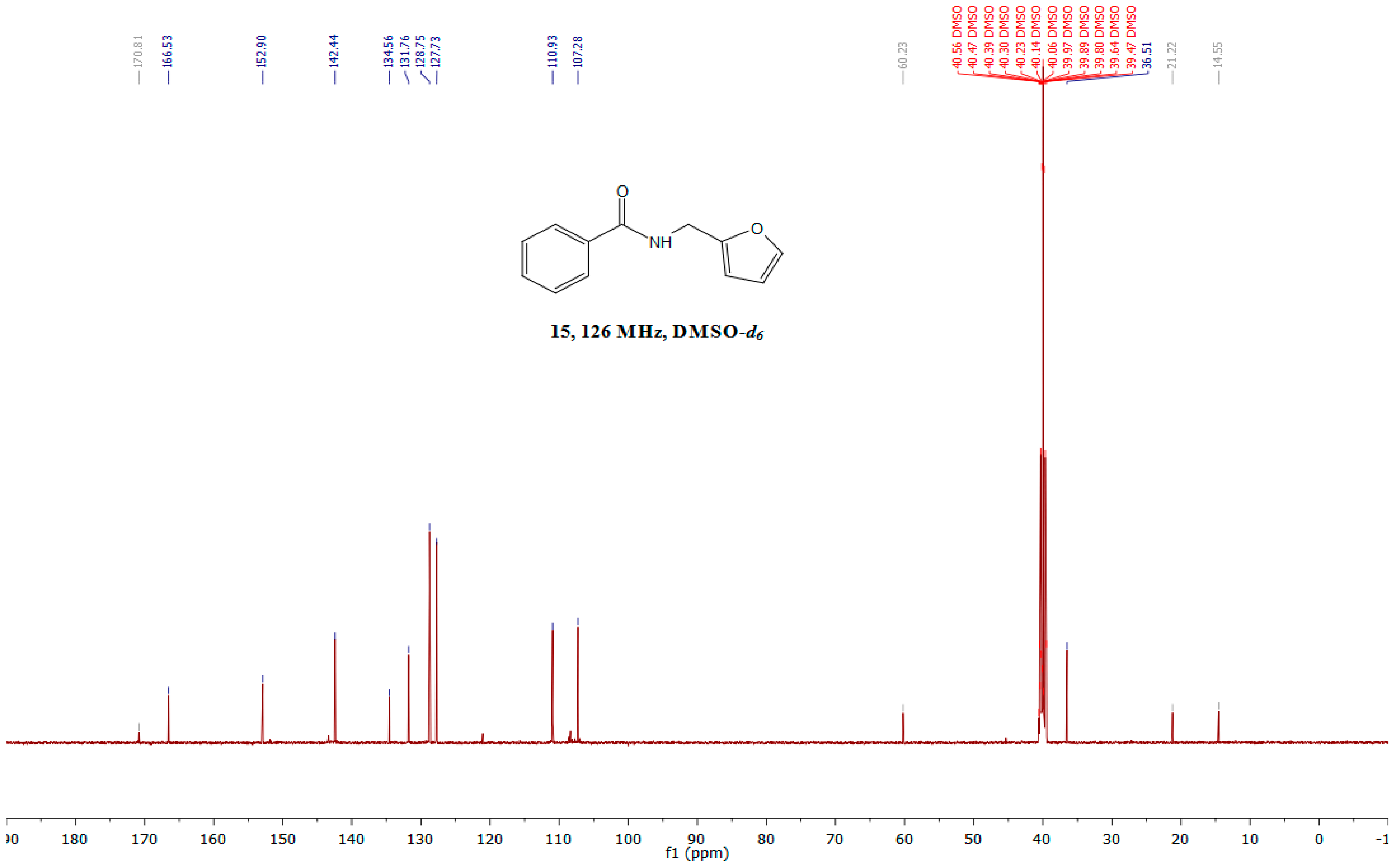
170.81
166.53
152.90
142.44
134.56
131.76
128.75
127.73
110.93
107.28
60.23
40.56 DMSO
40.47 DMSO
40.39 DMSO
40.30 DMSO
40.23 DMSO
40.14 DMSO
40.06 DMSO
39.97 DMSO
39.89 DMSO
39.80 DMSO
39.64 DMSO
39.47 DMSO
36.51
21.22
14.55
O
NH
O
15, 126 MHz, DMSO-d_6
f1 (ppm)

Figure S33.

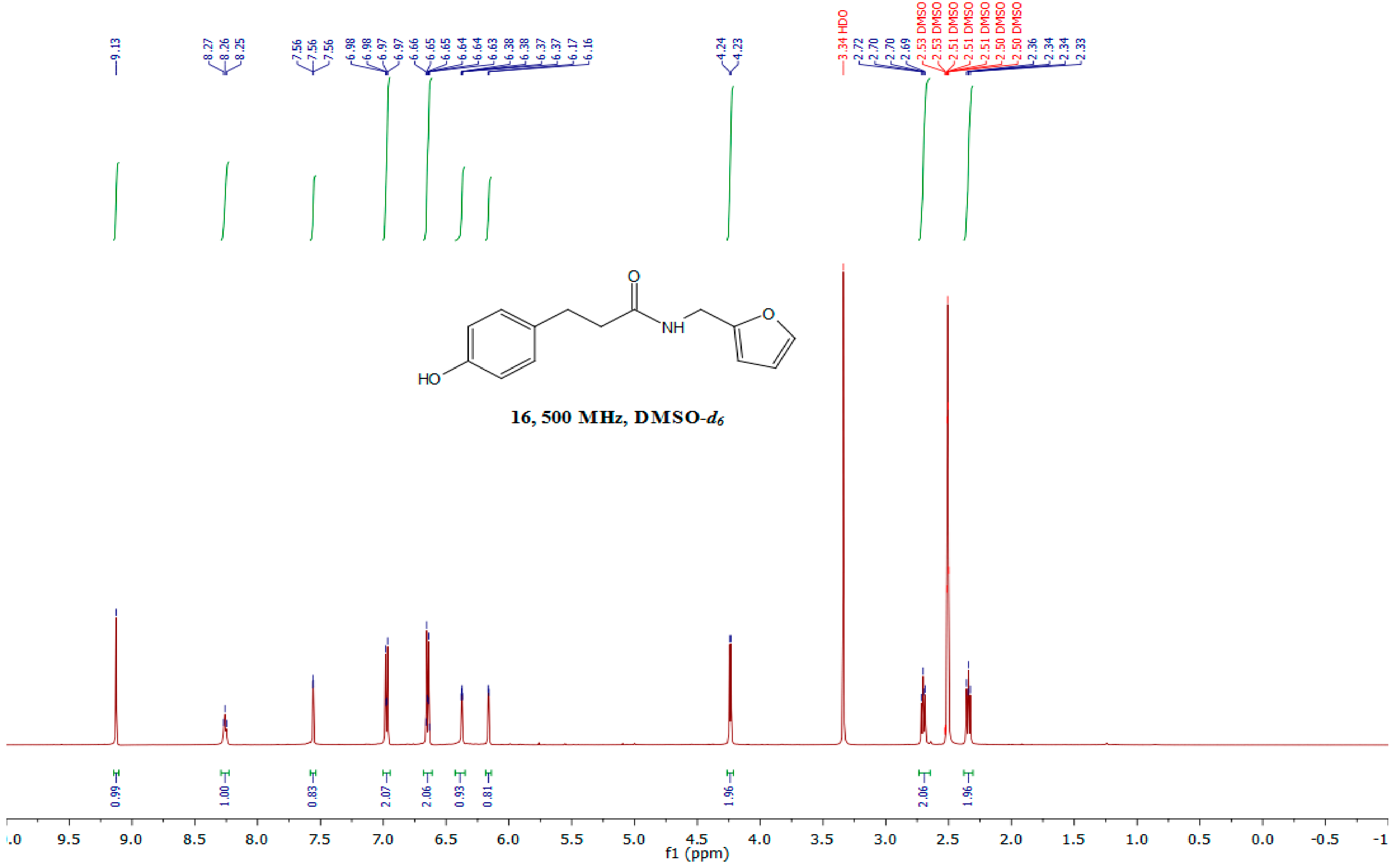

Figure S34.

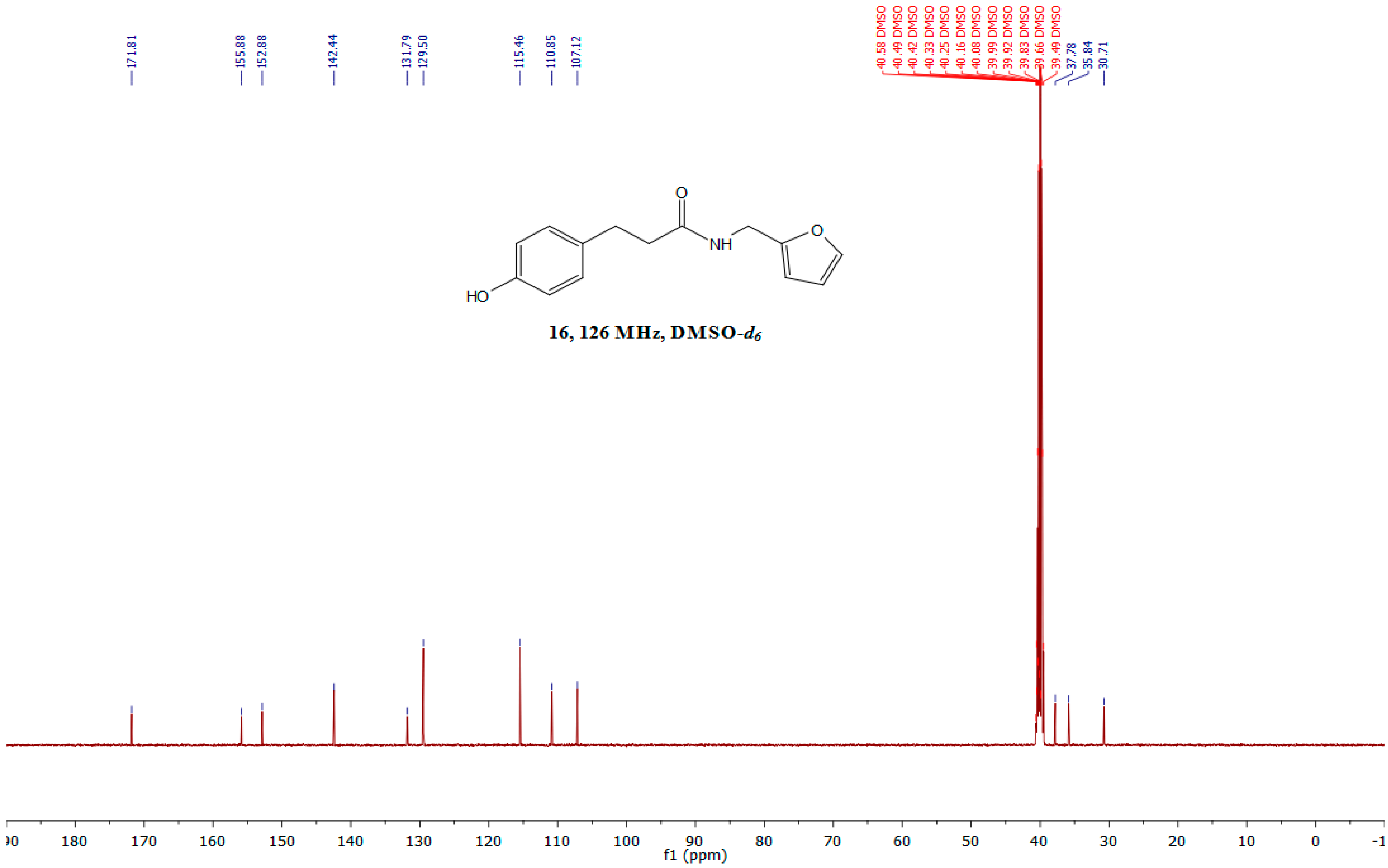
171.81
155.88
152.88
142.44
131.79
129.50
115.46
110.85
107.12
40.58 DMSO
40.49 DMSO
40.42 DMSO
40.33 DMSO
40.25 DMSO
40.16 DMSO
40.08 DMSO
39.99 DMSO
39.92 DMSO
39.83 DMSO
39.66 DMSO
39.49 DMSO
37.78
35.84
30.71
O
NH
O
HO
16, 126 MHz, DMSO-d_6
180
170
160
150
140
130
120
110
100
90
80
70
60
50
40
30
20
10
0
f1 (ppm)

Figure S35.

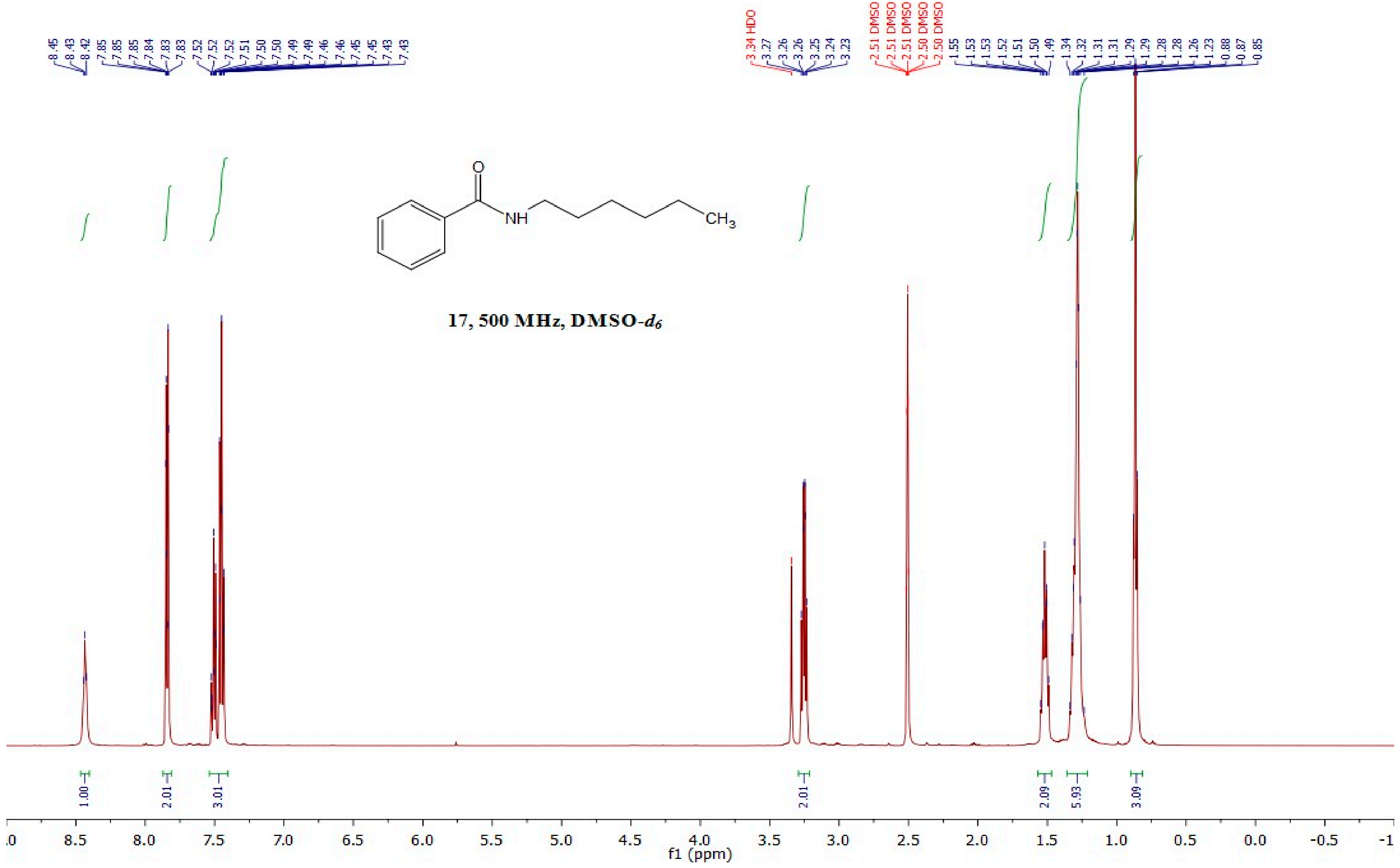

Figure S36.

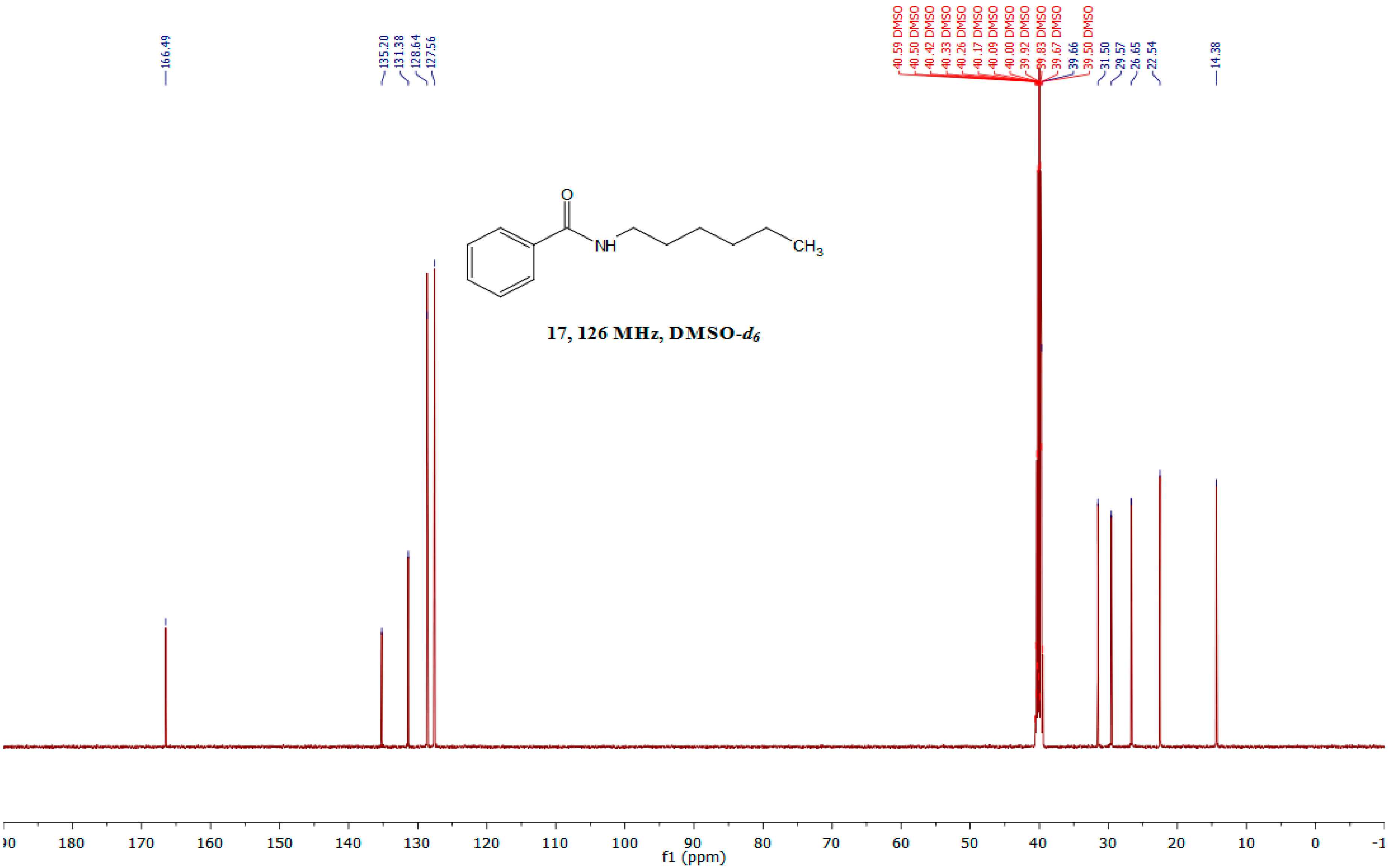
166.49
135.20
131.38
128.64
127.56
40.59 DMSO
40.50 DMSO
40.42 DMSO
40.33 DMSO
40.26 DMSO
40.17 DMSO
40.09 DMSO
40.00 DMSO
39.92 DMSO
39.83 DMSO
39.67 DMSO
39.66
39.50 DMSO
31.50
29.57
26.65
22.54
14.38
O
NH
CH3
17, 126 MHz, DMSO-d6
f1 (ppm)

Figure S37.

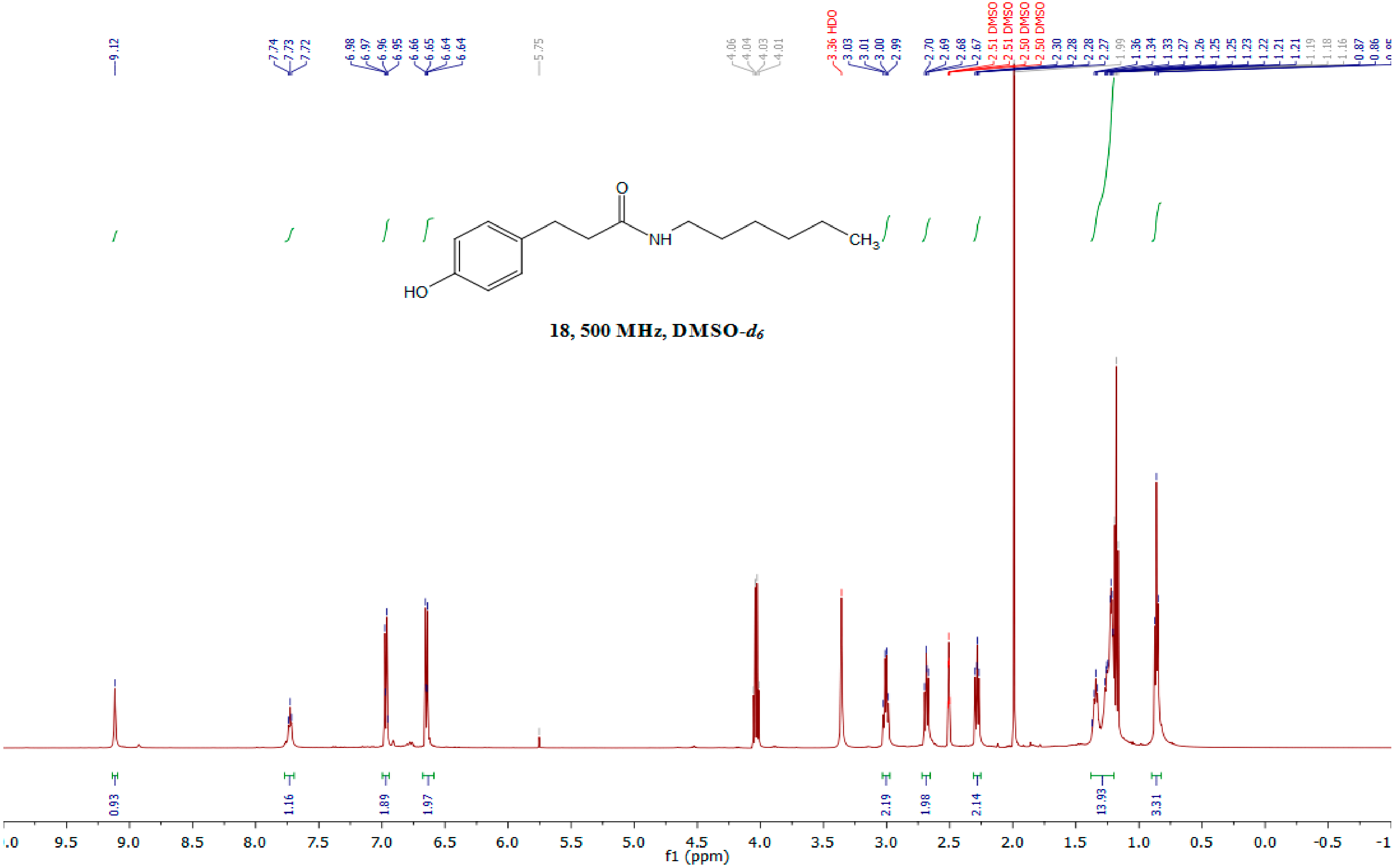

Figure S38.

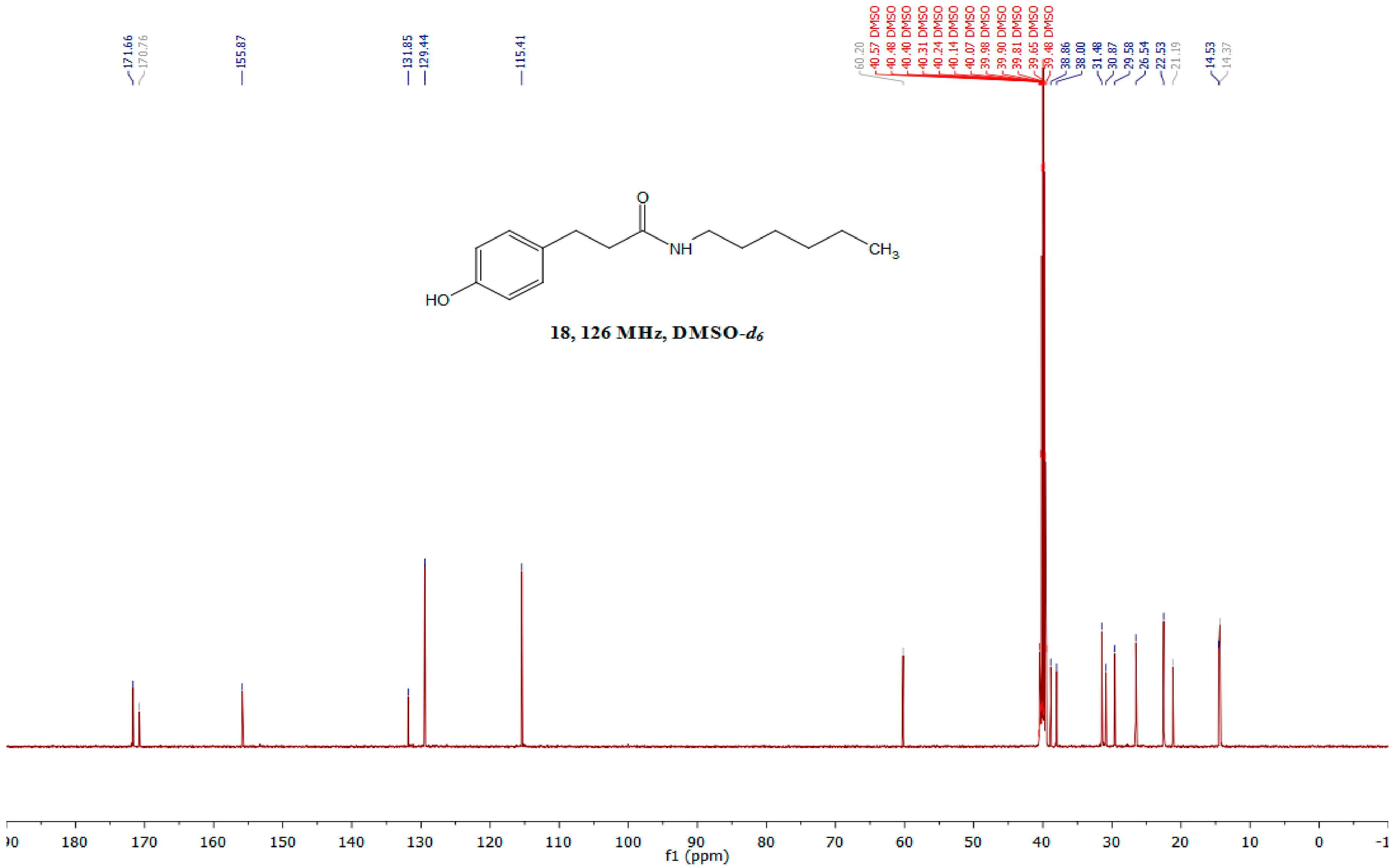
171.66
170.76
155.87
131.85
129.44
115.41
60.20
40.57 DMSO
40.48 DMSO
40.40 DMSO
40.31 DMSO
40.24 DMSO
40.14 DMSO
40.07 DMSO
39.98 DMSO
39.90 DMSO
39.81 DMSO
39.65 DMSO
39.48 DMSO
38.86
38.00
31.48
30.87
29.58
26.54
22.53
21.19
14.53
14.37
O
NH
CH3
HO
18, 126 MHz, DMSO-d6
180
170
160
150
140
130
120
110
100
90
80
70
60
50
40
30
20
10
0
f1 (ppm)

Figure S39.

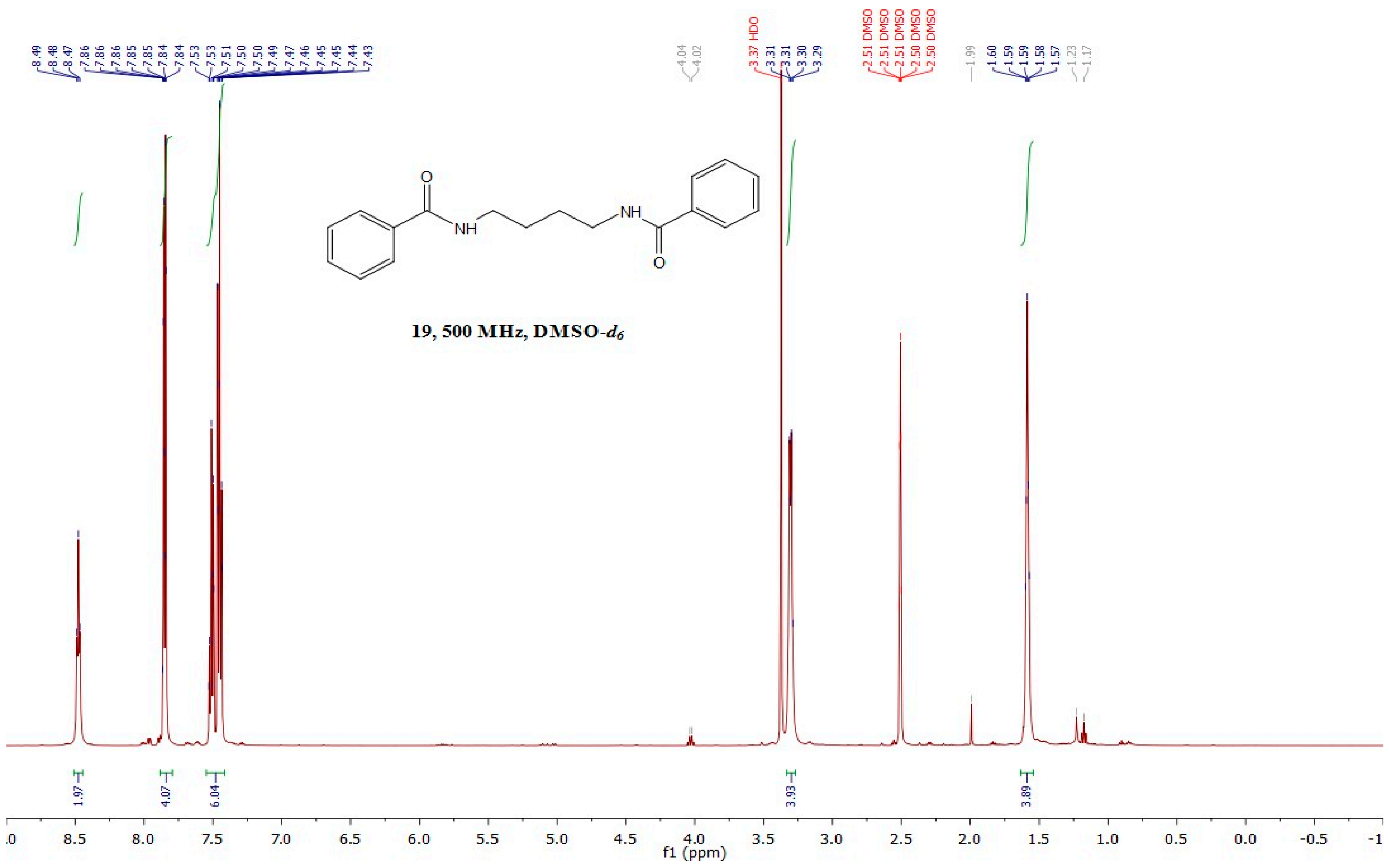

8.49
8.48
8.47
7.86
7.86
7.86
7.85
7.85
7.84
7.84
7.53
7.53
7.51
7.50
7.50
7.49
7.47
7.46
7.45
7.45
7.44
7.43
4.04
4.02
3.37 HDO
3.31
3.31
3.30
3.29
2.51 DMSO
2.51 DMSO
2.51 DMSO
2.50 DMSO
2.50 DMSO
1.99
1.60
1.59
1.59
1.58
1.57
1.23
1.17
O
NH
NH
O
19, 500 MHz, DMSO-d_6
1.97
4.07
6.04
3.93
3.89
f1 (ppm)

Figure S40.

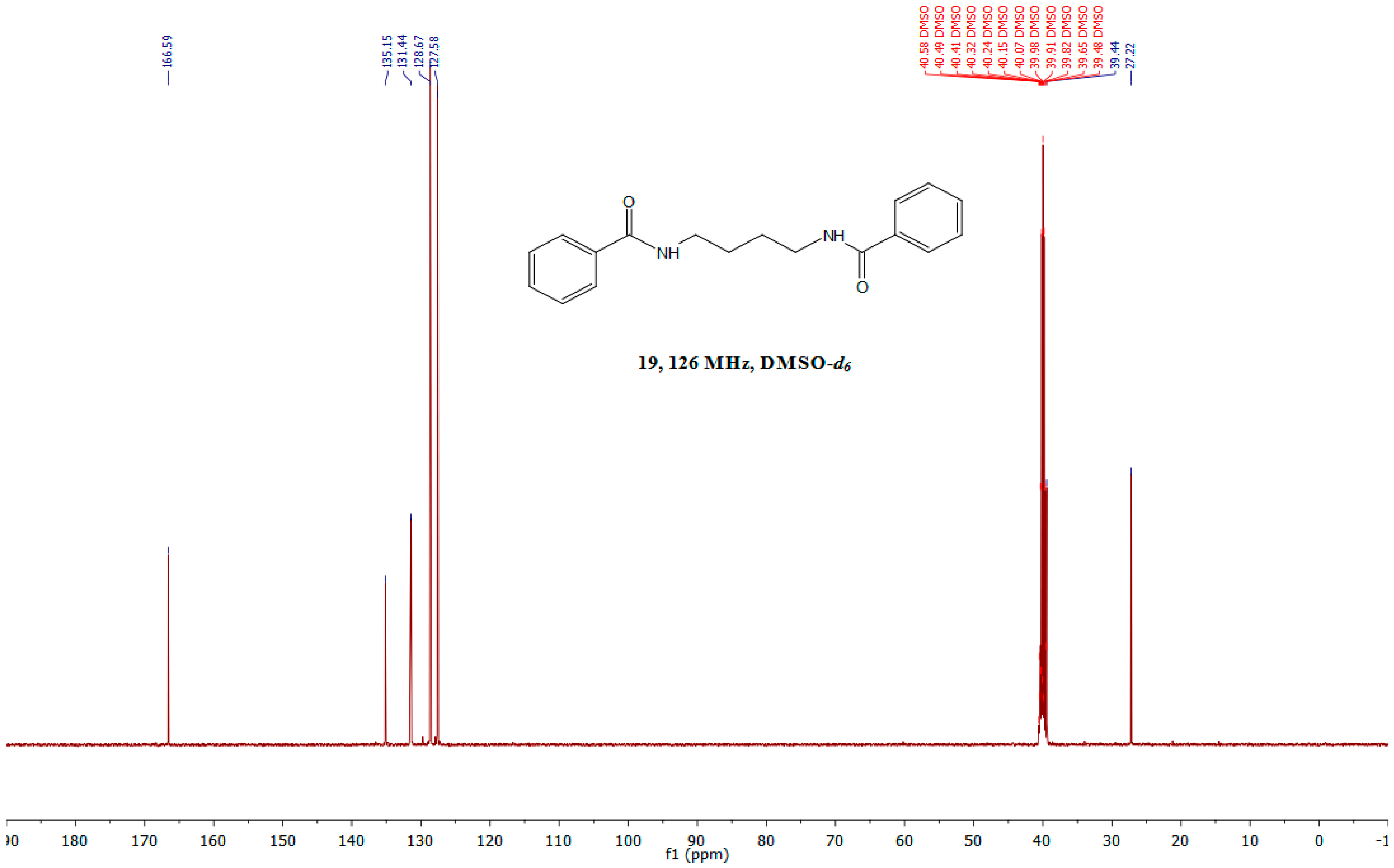
166.59
135.15
131.44
128.67
127.58
40.58 DMSO
40.49 DMSO
40.41 DMSO
40.32 DMSO
40.24 DMSO
40.15 DMSO
40.07 DMSO
39.98 DMSO
39.91 DMSO
39.82 DMSO
39.65 DMSO
39.48 DMSO
39.44
27.22
O
NH
NH
O
19, 126 MHz, DMSO-d_6
180
170
160
150
140
130
120
110
100
90
80
70
60
50
40
30
20
10
0
f1 (ppm)

Figure S41.

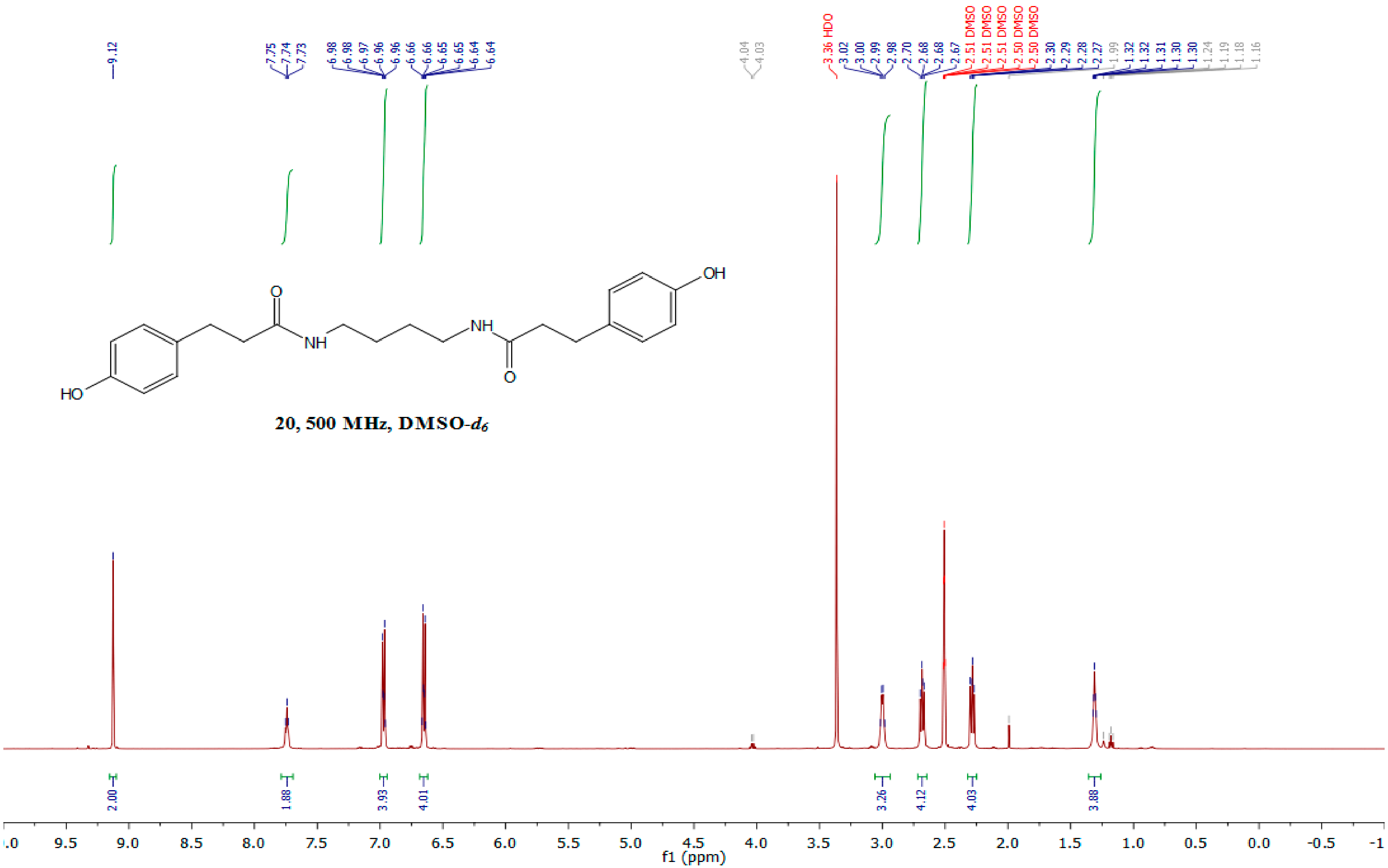

Figure S42.

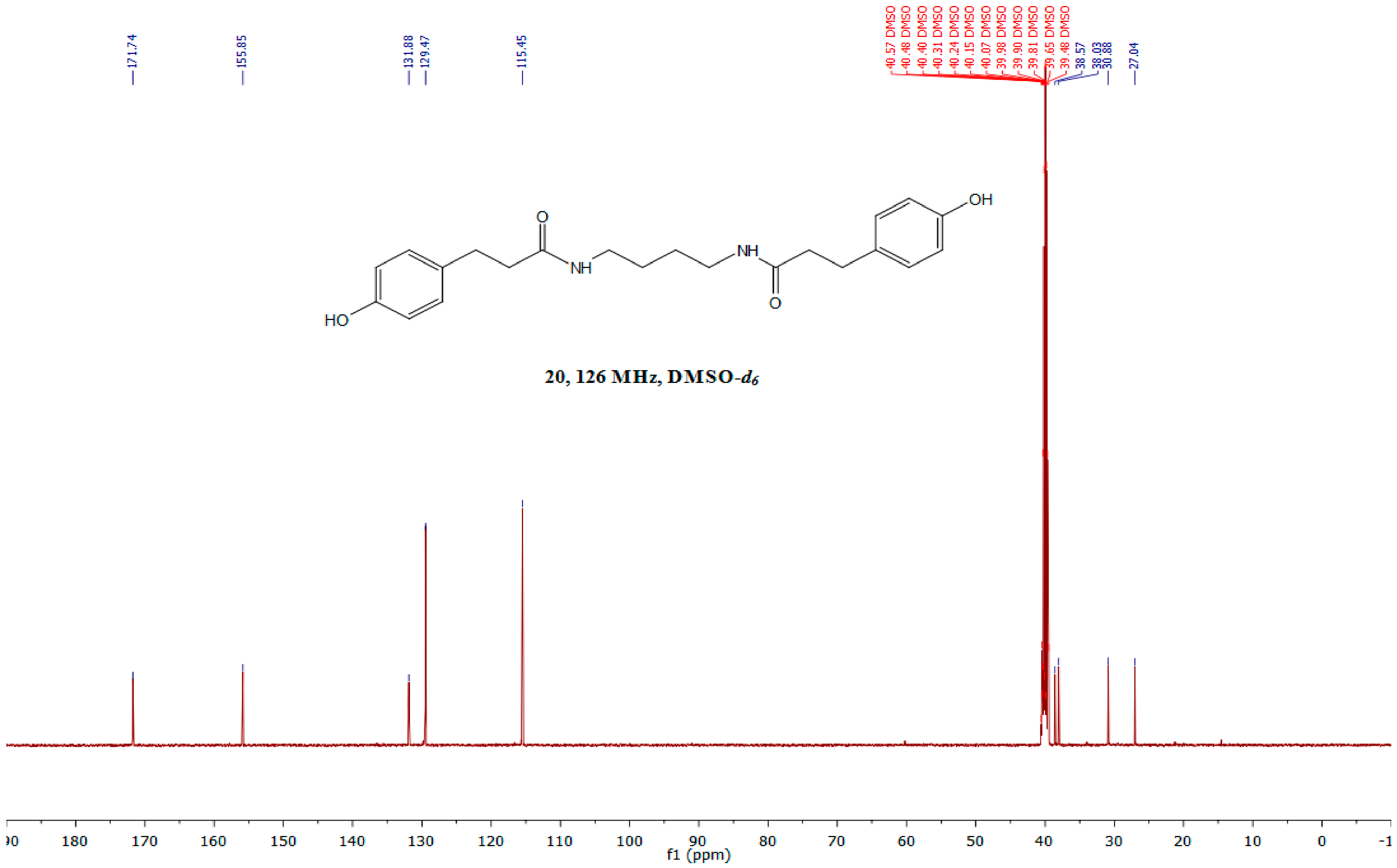
171.74
155.85
131.88
129.47
115.45
40.57 DMSO
40.48 DMSO
40.40 DMSO
40.31 DMSO
40.24 DMSO
40.15 DMSO
40.07 DMSO
39.98 DMSO
39.90 DMSO
39.81 DMSO
39.65 DMSO
39.48 DMSO
38.57
38.03
30.88
27.04
OH
O
NH
NH
O
HO
20, 126 MHz, DMSO-d_6
f1 (ppm)

Figure S43.

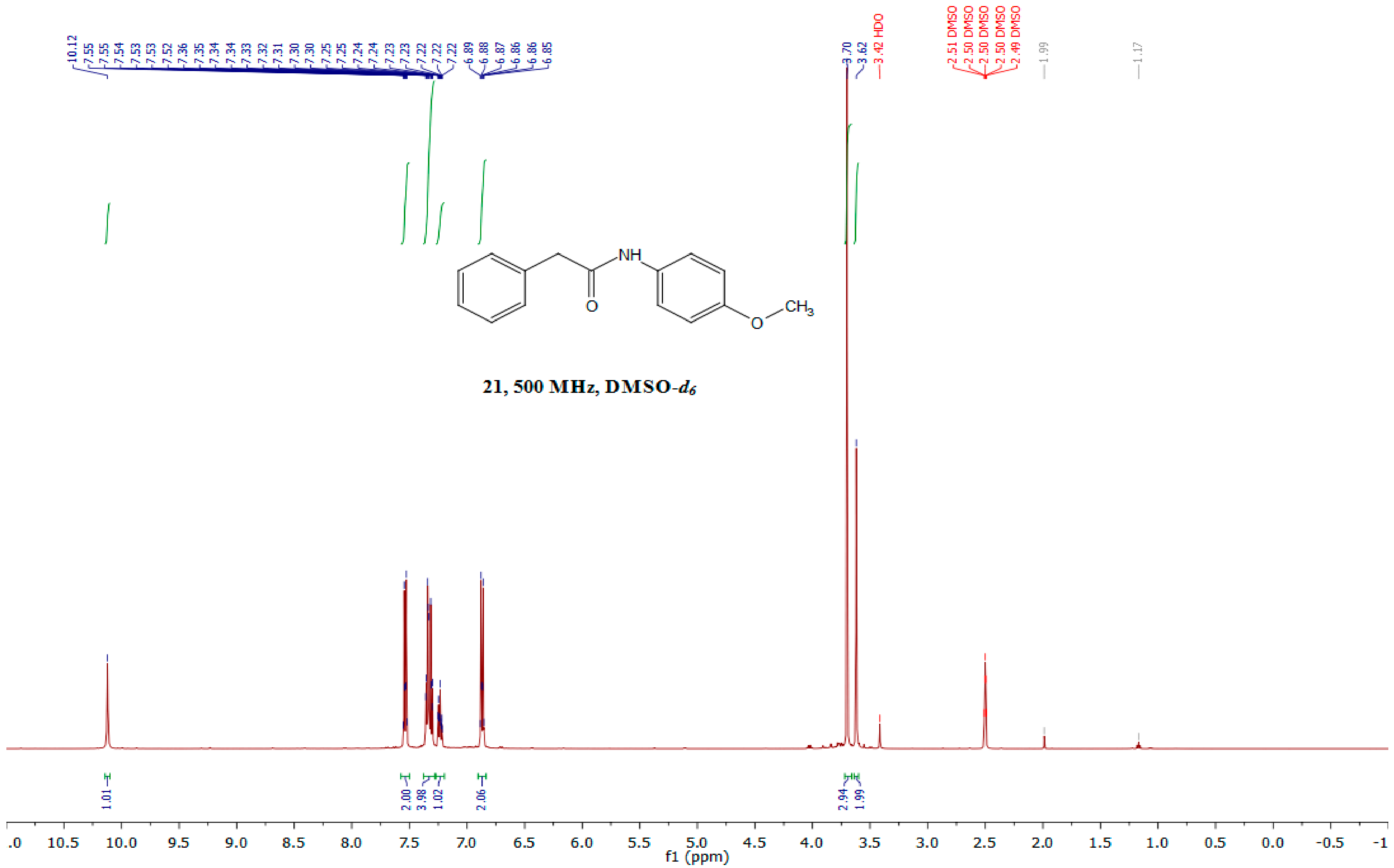
10.12
7.55
7.55
7.54
7.53
7.53
7.52
7.36
7.35
7.34
7.34
7.33
7.32
7.31
7.30
7.30
7.25
7.25
7.24
7.24
7.23
7.23
7.22
7.22
7.22
6.89
6.88
6.87
6.86
6.86
6.85
3.70
3.62
3.42 HDO
2.51 DMSO
2.50 DMSO
2.50 DMSO
2.50 DMSO
2.49 DMSO
1.99
1.17
NH
O
O
CH3
21, 500 MHz, DMSO-d6
1.01
2.00
3.98
1.02
2.06
2.94
1.99
f1 (ppm)

Figure S44.

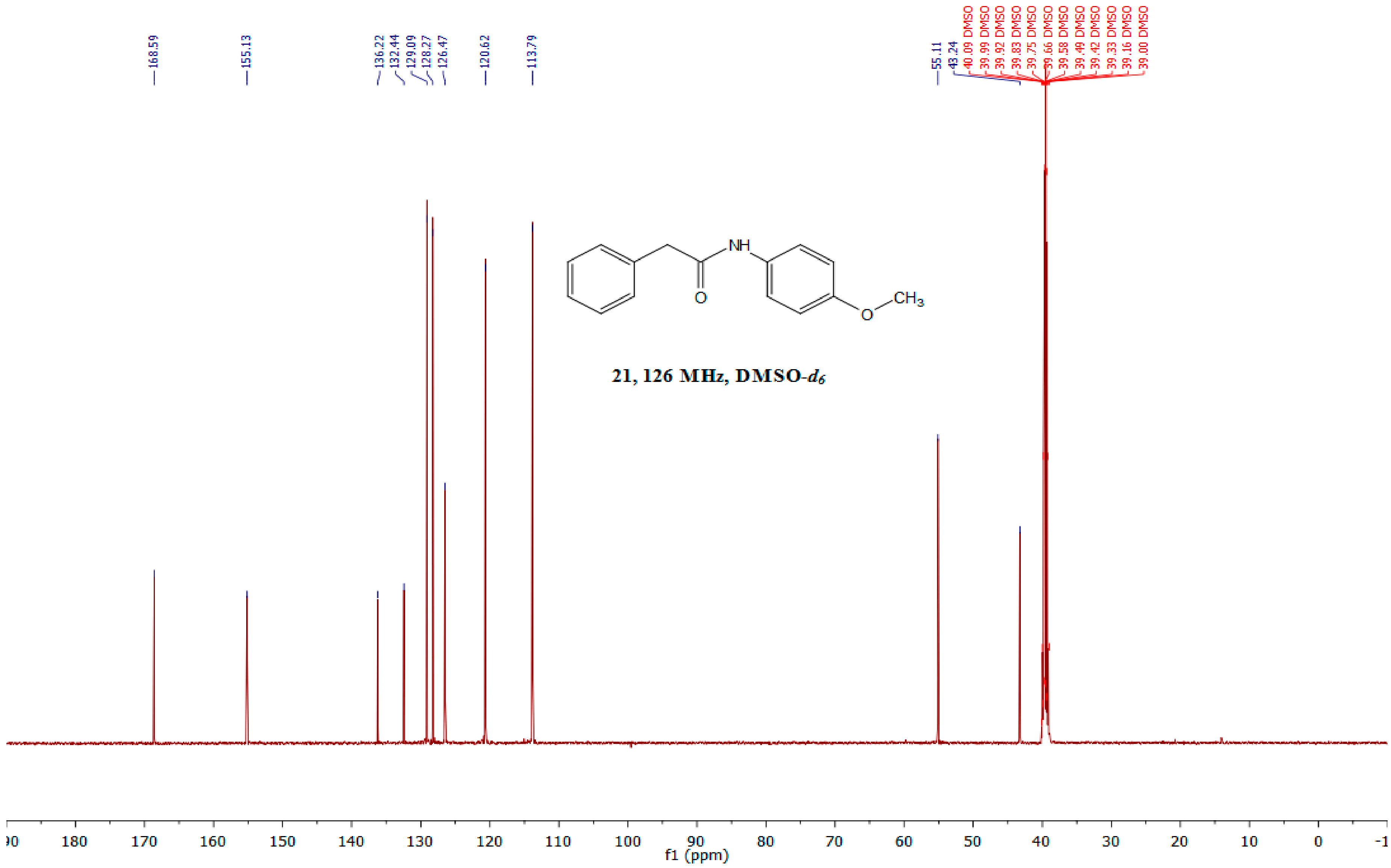
168.59
155.13
136.22
132.44
129.09
128.27
126.47
120.62
113.79
55.11
43.24
40.09 DMSO
39.99 DMSO
39.92 DMSO
39.83 DMSO
39.75 DMSO
39.66 DMSO
39.58 DMSO
39.49 DMSO
39.42 DMSO
39.33 DMSO
39.16 DMSO
39.00 DMSO
NH
O
O
CH3
21, 126 MHz, DMSO-d6
180
170
160
150
140
130
120
110
100
90
80
70
60
50
40
30
20
10
0
f1 (ppm)

Figure S45.

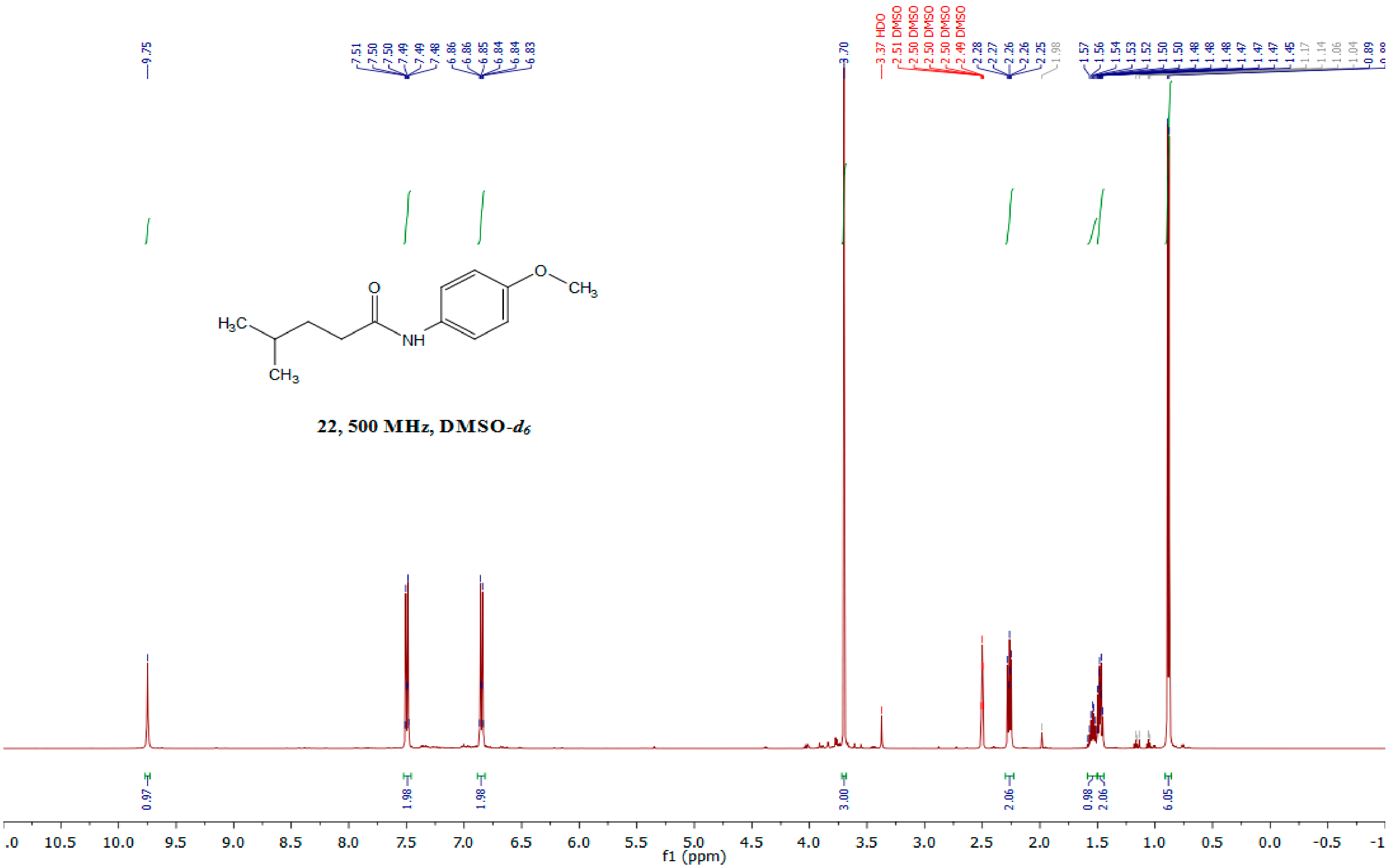

Figure S46.

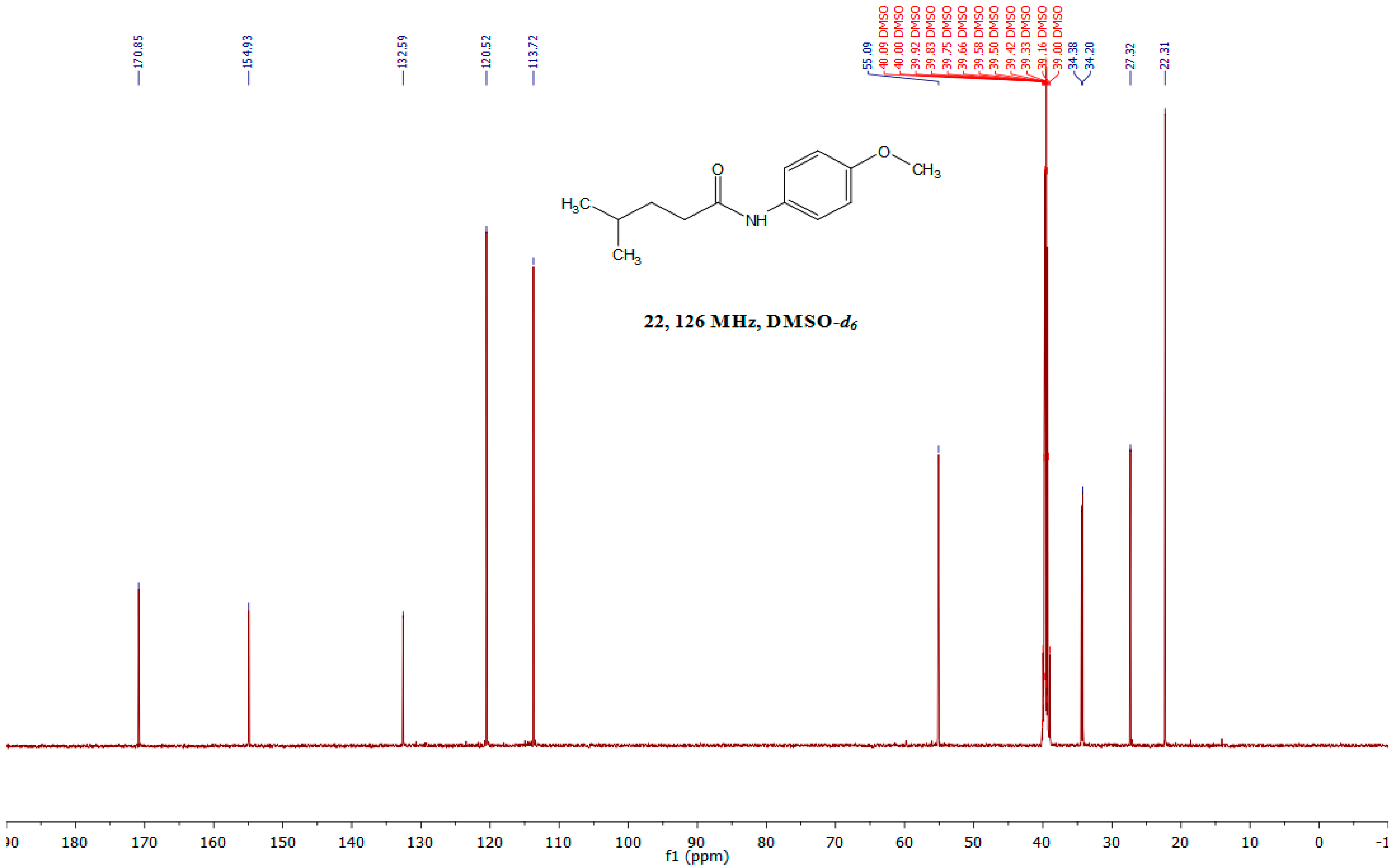

170.85
154.93
132.59
120.52
113.72
55.09
40.09 DMSO
40.00 DMSO
39.92 DMSO
39.83 DMSO
39.75 DMSO
39.66 DMSO
39.58 DMSO
39.50 DMSO
39.42 DMSO
39.33 DMSO
39.16 DMSO
39.00 DMSO
34.38
34.20
27.32
22.31
22, 126 MHz, DMSO-d_6
f1 (ppm)

Figure S47.

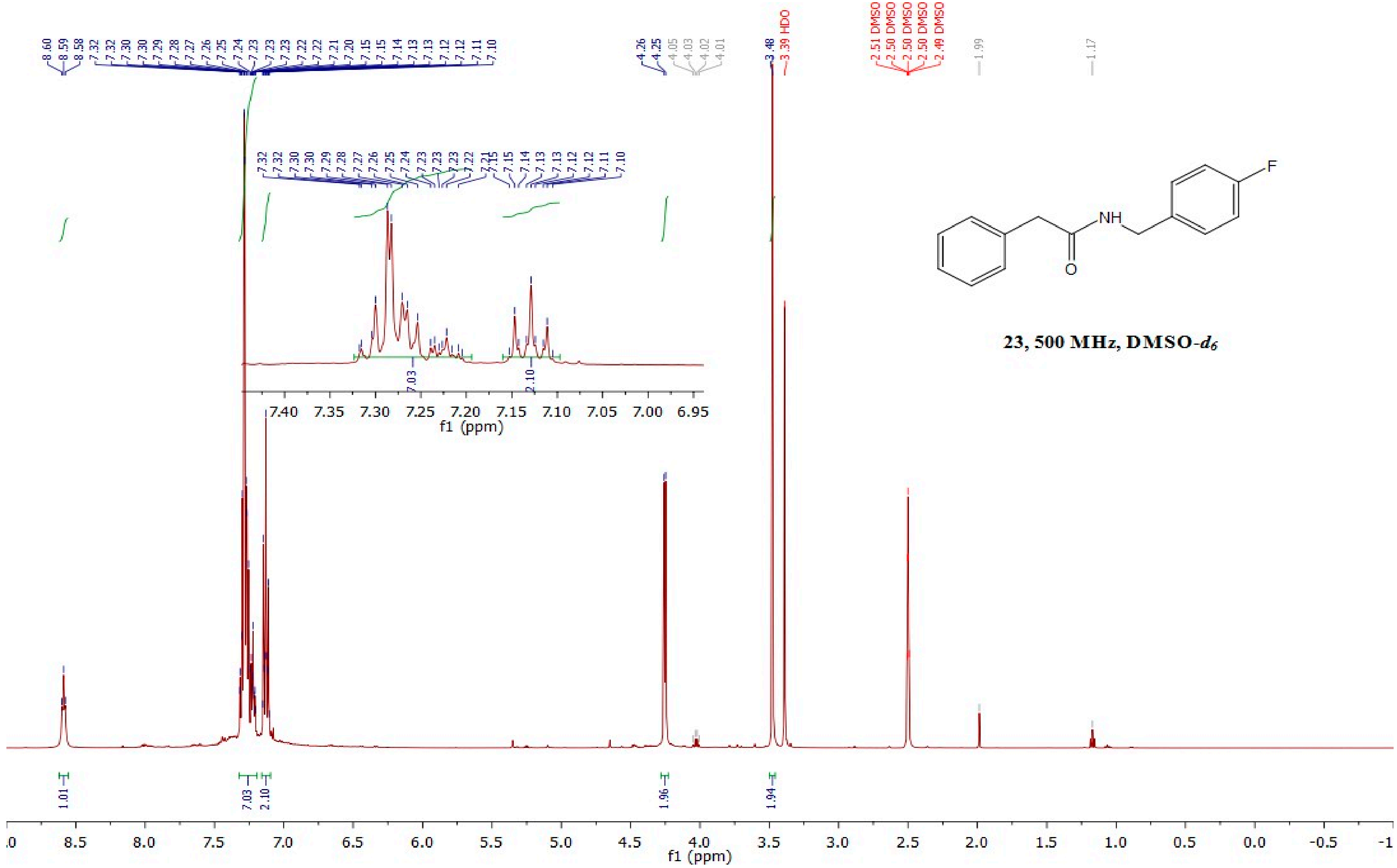
23, 500 MHz, DMSO-d_6
NH
F
O
f1 (ppm)
3.39 HDO
2.51 DMSO
2.50 DMSO
2.50 DMSO
2.50 DMSO
2.49 DMSO

Figure S48.

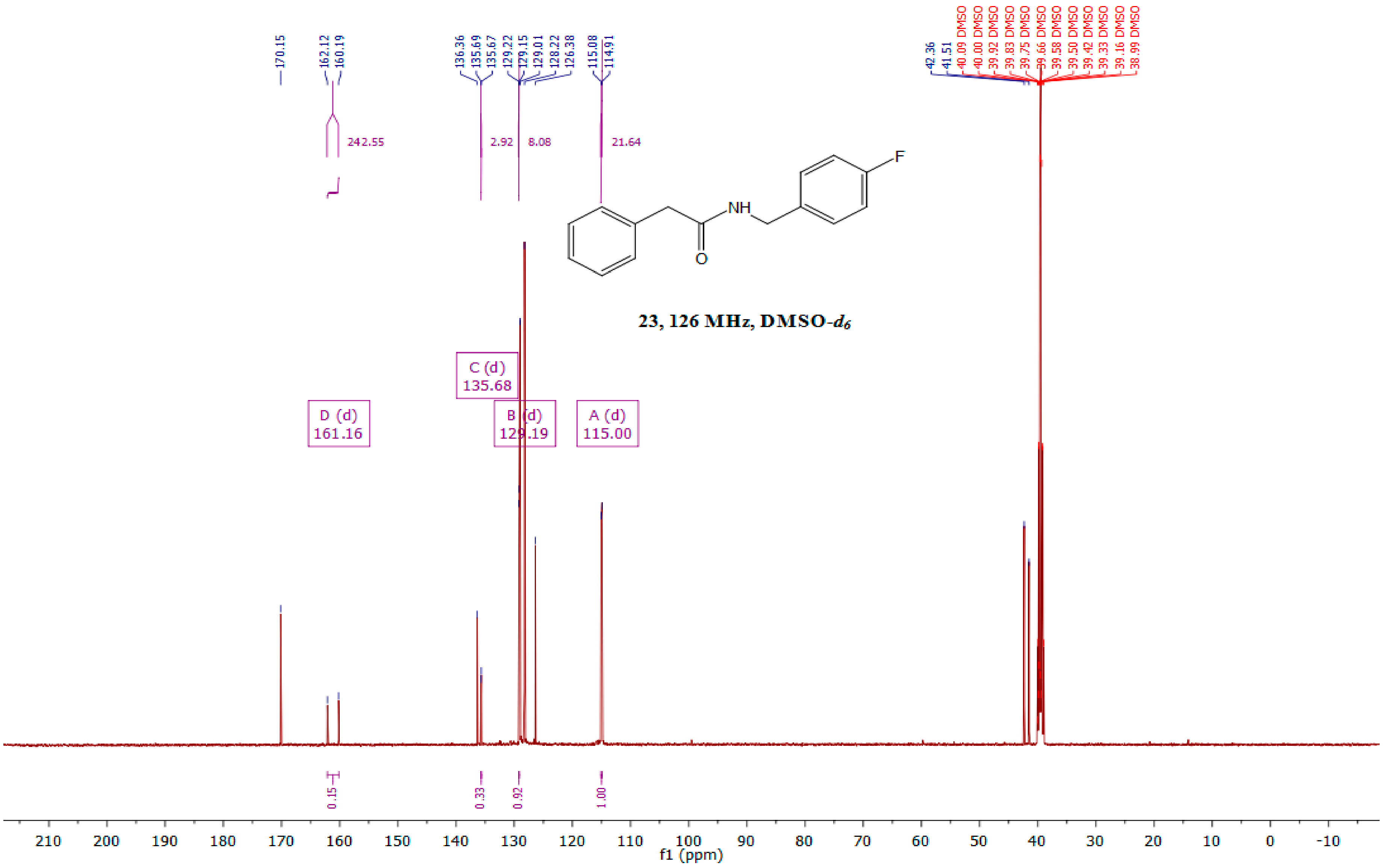

170.15
162.12
160.19
136.36
135.69
135.67
129.22
129.15
129.01
128.22
126.38
115.08
114.91
42.36
41.51
40.09 DMSO
40.00 DMSO
39.92 DMSO
39.83 DMSO
39.75 DMSO
39.66 DMSO
39.58 DMSO
39.50 DMSO
39.42 DMSO
39.33 DMSO
39.16 DMSO
38.99 DMSO
242.55
2.92
8.08
21.64
NH
O
F
23, 126 MHz, DMSO-d6
C (d)
135.68
D (d)
161.16
B (d)
129.19
A (d)
115.00
0.15
0.33
0.92
1.00
f1 (ppm)

Figure S49.

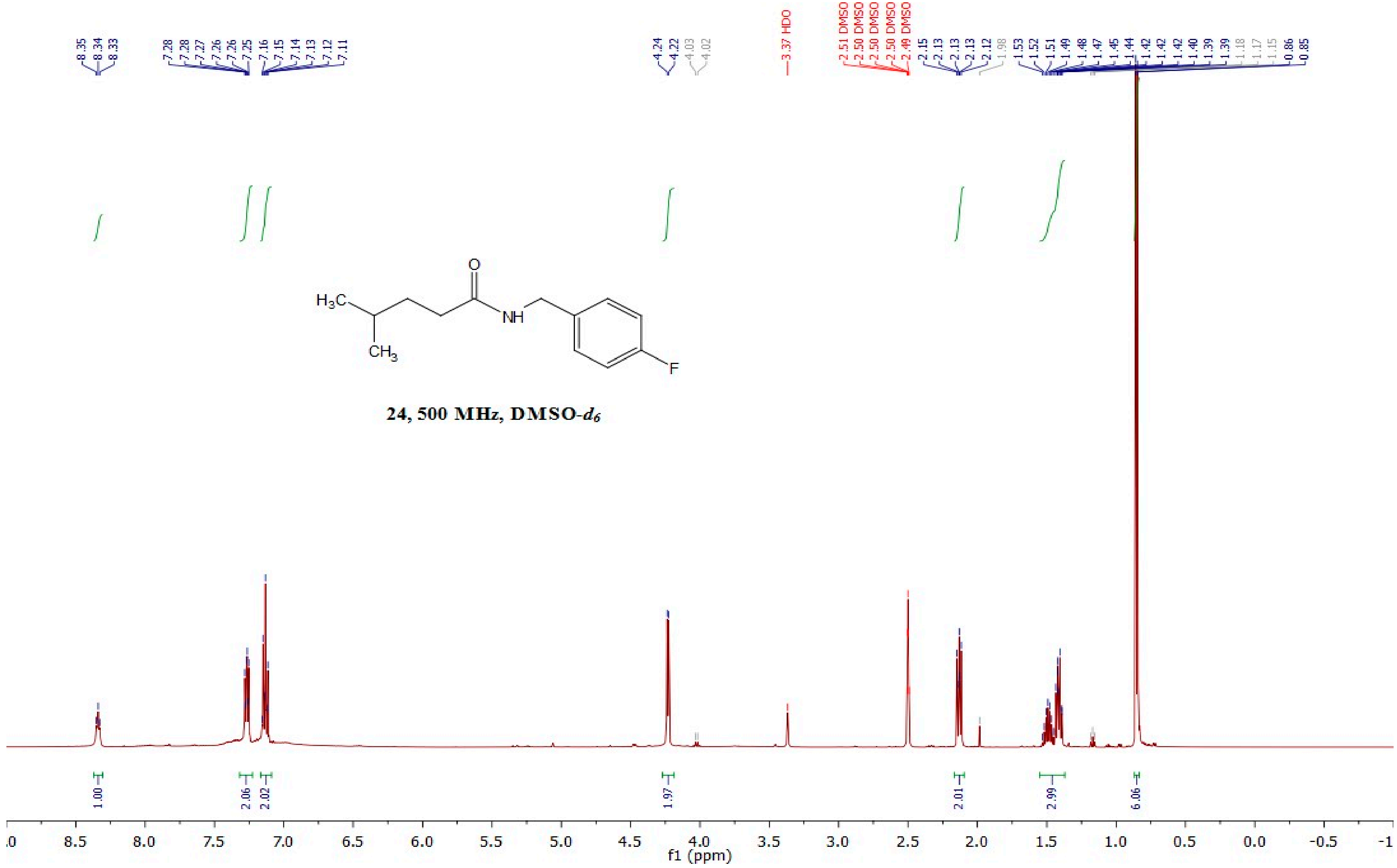

Figure S50.

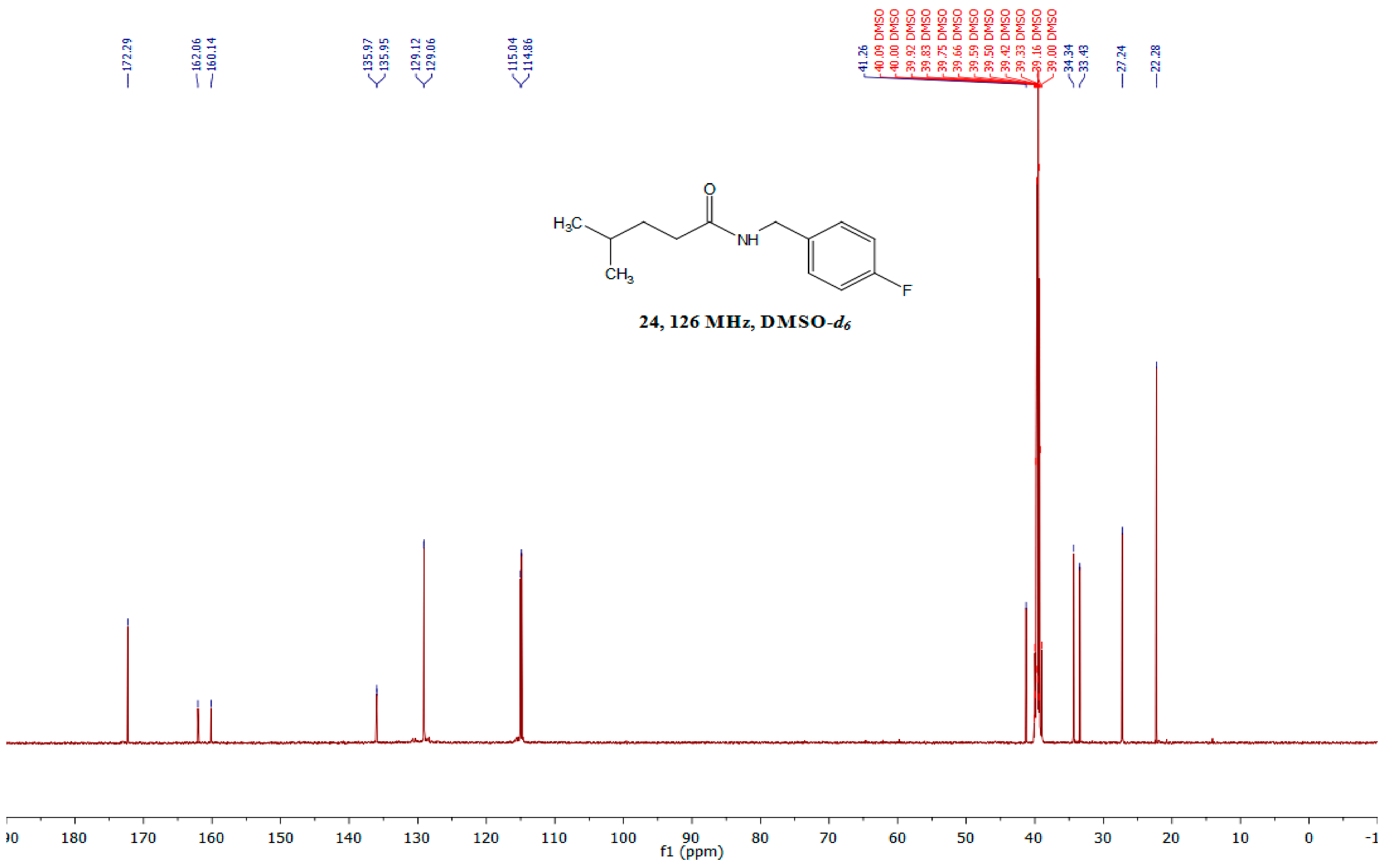
172.29
162.06
160.14
135.97
135.95
129.12
129.06
115.04
114.86
41.26
40.09 DMSO
40.00 DMSO
39.92 DMSO
39.83 DMSO
39.75 DMSO
39.66 DMSO
39.59 DMSO
39.50 DMSO
39.42 DMSO
39.33 DMSO
39.16 DMSO
39.00 DMSO
34.34
33.43
27.24
22.28
24, 126 MHz, DMSO-d_6
f1 (ppm)

Figure S51.

References

1. Y. Wang, D. Zhu, L. Tang, S. Wang, Z. Wang, *Angew. Chem. Int. Ed.*, 2011, **50**, 8917.
2. Y. Teo, F. Yong, I. K. Ithnin, S. T. Yio, Z. Lin, *Eur. J. Org. Chem.*, 2013, 515.
3. S. S. Kulkarni, X. Hu, R. Manetsch, *Chem. Commun.*, 2013, **49**, 1193.
4. N. A. Braun, M. Ousmer, J. D. Bray, D. Bouchu, K. Peters, E. M. Peters, M. A. Ciufolini, *J. Org. Chem.*, 2000, **65**, 4397-4408.
5. X. Cui, Y. Zhang, F. Shi, Y. Deng, *Chem. Eur. J.*, 2011, **17**, 1021.
6. D. Dubé, A. A. Scholte, *Tetrahedron Lett.*, 1999, **40**, 2295.
7. N. Wang, X. Zou, J. Ma, F. Li, Chem. Commun., 2014, **50**, 8303-8305.
8. S. N. Rao, D. C. Mohan, S. Adimurthy, *Org. Lett.*, 2013, **15**, 1496-1499.
9. Z. Zhang, Z. Yin, N. A. Meanwell, J. F. Kadow, T. Wang, *Org. Lett.*, 2003, **5**, 3399-3402.
10. K. U. Minchitha, H. N. Hareesh, N. Nagaraju, N. Kathyayini, *J. Nanosci. Nanotechnol.*, 2018, **18**, 426-433.
11. Z. Fu, J. Lee, B. Kang, S. H. Hong, *Org. Lett.*, 2012, **14**, 6028-6031.
